# Advance in peptide-based drug development: delivery platforms, therapeutics and vaccines

**DOI:** 10.1038/s41392-024-02107-5

**Published:** 2025-03-05

**Authors:** Wenjing Xiao, Wenjie Jiang, Zheng Chen, Yu Huang, Junyi Mao, Wei Zheng, Yonghe Hu, Jianyou Shi

**Affiliations:** 1 Department of Pharmacy, The General Hospital of Western Theater Command, Chengdu, 610083 China; 2 Department of Pharmacy, Personalized Drug Therapy Key Laboratory of Sichuan Province, Sichuan Academy of Medical Sciences & Sichuan Provincial People’s Hospital, School of Medicine, University of Electronic Science and Technology of China, Chengdu, 610072 China; 3 School of Life Science and Engineering, Southwest Jiaotong University, Chengdu, 610031 China; 4 School of Chinese Materia Medica, Tianjin University of Traditional Chinese Medicine, Tianjin, 301617 China; 5 Department of Integrative Medicine, Xinqiao Hospital, Army Medical University, Chongqing, 400037 China; 6 School of Medicine, Southwest Jiaotong University, Chengdu, 610031 China

**Keywords:** Drug discovery, Medicinal chemistry, Drug delivery, Business strategy in drug development

## Abstract

The successful approval of peptide-based drugs can be attributed to a collaborative effort across multiple disciplines. The integration of novel drug design and synthesis techniques, display library technology, delivery systems, bioengineering advancements, and artificial intelligence have significantly expedited the development of groundbreaking peptide-based drugs, effectively addressing the obstacles associated with their character, such as the rapid clearance and degradation, necessitating subcutaneous injection leading to increasing patient discomfort, and ultimately advancing translational research efforts. Peptides are presently employed in the management and diagnosis of a diverse array of medical conditions, such as diabetes mellitus, weight loss, oncology, and rare diseases, and are additionally garnering interest in facilitating targeted drug delivery platforms and the advancement of peptide-based vaccines. This paper provides an overview of the present market and clinical trial progress of peptide-based therapeutics, delivery platforms, and vaccines. It examines the key areas of research in peptide-based drug development through a literature analysis and emphasizes the structural modification principles of peptide-based drugs, as well as the recent advancements in screening, design, and delivery technologies. The accelerated advancement in the development of novel peptide-based therapeutics, including peptide-drug complexes, new peptide-based vaccines, and innovative peptide-based diagnostic reagents, has the potential to promote the era of precise customization of disease therapeutic schedule.

## Introduction

Since the introduction of insulin in 1922, peptide drugs have become a promising modality in human therapeutics.^1^ Peptides offer the potency of biologics yet retain drug-like properties for oral availability and tissue penetration. Their superior specificity in targeting interactions, tunable half-lives, and typically lower toxicity and immunogenicity give peptides advantages over other modalities.^2,3^ Manufacturing peptides also costs less than protein therapeutics.^4^ With precise rational design and advances enabling improved bioavailability, peptide drugs are poised to overcome limitations of traditional small molecules and biologics.^3^

Historically, early peptide drugs were primarily sourced from specific animals, including reptiles, amphibians, arachnids, gastropods, and venomous mammals.^5^ However, the rarity of these animals and the challenges associated with extracting complex compounds from them hindered peptide drug development for several decades.^6–9^ It wasn’t until the 1950s, with the emergence of peptide synthesis technologies, that peptides experienced accelerated advancement. Pioneering breakthroughs, such as Vincent Du Vigneaud’s synthesis of oxytocin and vasopressin^10–12^ and Robert Merrifield’s solid-phase peptide synthesis,^13^ paved the way for large-scale peptide production and the approvals of pioneering peptide drugs like goserelin for cancer in 1989^14^ and enfuvirtide for HIV in 2003.

Today, with nearly 100 approved peptide drugs worldwide and ongoing transitions from preclinical to clinical trials, the peptide therapeutics market continues to grow.^15^ Significant advancements include the approval of semaglutide (Rybelsus^®^, Novo Nordisk A/S) as the first oral glucagon-like peptide-1 receptor agonist (GLP-1RA) for managing type 2 diabetes mellitus (T2DM) and weight loss.^16,17^ Sales data from 2024 highlights the market dominance of semaglutide formulations, with semaglutide injections (Ozempic^®^) led peptide drug sales, totaling $138.90 hundred million USD. Other semaglutide formulations followed suit, with injectable Trulicity^®^ at $71.30 hundred million USD and oral Rybelsus® at $27.20 hundred million USD (Fig. 8a), reflecting the growing demand for peptide therapeutics.

Nowadays, research efforts in peptide development continue to advance rapidly. In November 2023, Eli Lilly introduced tirzepatide (Mounjaro^®^/Zepbound^®^), the pioneering dual glucose-dependent insulinotropic polypeptide (GIP) and GLP-1 RA for weight management and glycemic control. It demonstrated superior performance in the SURPASS phase III trials over single receptor agonists like dulaglutide and semaglutide.^18^ Moreover, promising candidates are emerging, such as retaglutide for treating T2DM, fatty liver disease, and obesity by targeting the glucagon receptor (GCGR), gastric inhibitory polypeptide receptor (GIPR), and glucagon-like peptide-1 receptor (GLP-1R).^19^ Additionally, diagnostic applications like the first peptide radiopharmaceutical [^68^Ga]Ga-DOTA-TOC for diagnosing somatostatin receptor-positive neuroendocrine tumors (NETs) underscore the versatility of peptide-based technologies.^20^ To provide a clear presentation of the boom in peptide drug research, we have updated the data on marketed peptides and clinical trials from Wang et al.’s recent study^2^ (Table 3).

Despite the advancements, challenges remain, particularly concerning the rapid clearance and degradation of peptide drugs, necessitating subcutaneous injection and increasing patient discomfort. However, ongoing developments in structural modifications and delivery systems hold promise for enabling oral peptide formulations with enhanced stability, bioavailability, and patient compliance.^21^ Notably, cell-targeting peptide (CTP)-based platforms and peptide-drug conjugates (PDCs) show particular promise in overcoming challenges associated with traditional small molecule therapies, enhancing efficiency, and reducing adverse effects,^22–27^ with multiple platforms now in clinical trials (Tables 5 and 6).

Furthermore, peptide innovation extends to the vaccine field, where peptide-based subunits offer heightened specificity, safety, and quality control compared to traditional whole-pathogen vaccines,^28^ transitioning vaccine development from the empirical whole-pathogen era to the defined subunit era. This transition has enabled the proliferation of preclinical trials for peptide vaccines. During the singular year span of 2023–2024, over 200 clinical trials involving peptide vaccines for infectious diseases and cancer prevention and treatment were documented on ClinicalTrials.gov. Here, we provide updated phase III trial data up to 2024 (Table 7).

**Fig. 1:**
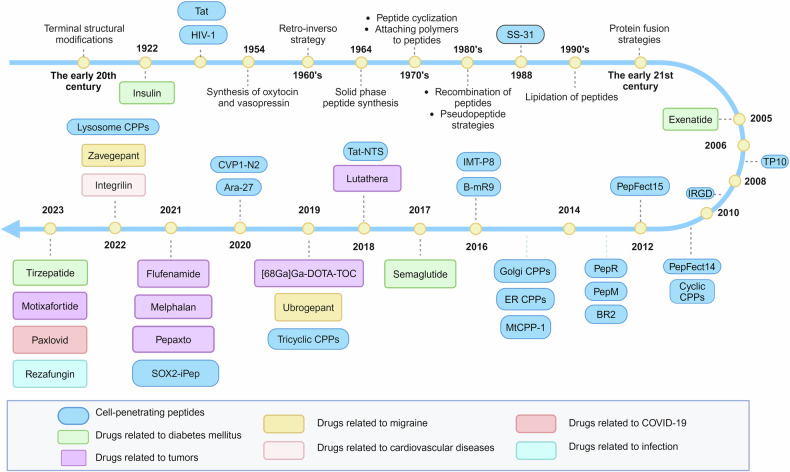
Visualization of important events in the development of peptide drugs. In this figure, important peptide drugs are classified according to the disease to which they correspond. In addition, the progress of optimization strategy of peptide drugs and Cell-penetrating peptides are also listed in this figure

This review aims to provide a comprehensive analysis of the current status of peptide-based drug development (Fig. 1), emphasizing recent therapeutic advances, delivery systems, and vaccine innovations. Additionally, we discuss future advancements and obstacles in peptide therapeutics, highlighting the ongoing evolution of this promising field.

## Advance of peptide-based drug and development trend

### Characteristics of peptide-based drugs

Peptides as therapeutic agents trace back to 1922, when Dr. Frederick Banting and colleagues first extracted insulin from animals and applied it in the treatment of type I diabetes.^29^ Since then, peptides as therapeutic agents have played a pivotal role in human physiology, serving as hormones, neurotransmitters, growth factors, antimicrobials, and vaccines, among other functions.^30–34^

**Fig. 2:**
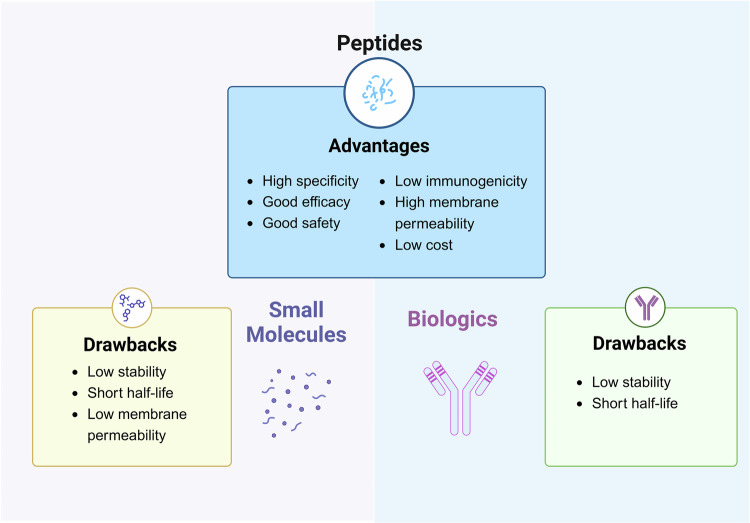
Advantages and limitations of peptide-based drugs. Peptides, small molecules, and biologics represent three distinct categories of therapeutic agents. Peptides possess certain advantages when compared to the other two classes. However, small molecules exhibit specific disadvantages relative to peptides, and biologics also present their own set of limitations. Figure 2 was created with biorender.com

Peptides represent a discrete family of pharmacological substances that lie between tiny molecules and proteins in molecular weight, yet display distinctive biological and physicochemical characteristics (Fig. 2). Therapeutic peptides are a kind of amino acid sequences that combine properties from large proteins or other biologics with small molecule medications.^3,35^ Typically, these sequences have fewer than 50 residues in their chain. Peptides have advantages over proteins and antibodies, including lesser immunogenicity and lower cost of manufacture. The attachment of peptides to specific receptors elicits subsequent physiological responses, similar to the mechanism of action observed in protein and antibody medications. Peptides, on the other hand, can penetrate tissues more deeply because of their smaller size. Furthermore, peptides typically have fewer side effects because they are less immunogenic than therapeutic proteins and antibodies.^36,37^ Chemical synthesis is widely regarded as the 、the most advanced technology for the production of therapeutic peptides particularly following the advent of solid-phase peptide synthesis (SPPS).^38^ The primary advantages of SPPS are the facilitation of efficient separation of peptide products from impurities and byproducts.^39^ Synthetic therapeutic peptides are a great option due to their lower cost and higher quality control compared to peptides or proteins obtained through enzymatic processes or recombinant technology. Furthermore, therapeutic peptides usually have a length of 10–50 amino acids, whereas antibodies have a binding site of 75 kDa.^40–42^ This means that peptides have higher specific activity per unit mass (15–60 times higher), which lowers the cost per unit of active medicine. Peptides are also less expensive commercially since they are more stable and may be kept at room temperature.^43^

Therapeutic peptides offer several advantages compared to traditional small molecule pharmaceuticals. First of all, peptides typically represent the smallest functional components of proteins, thereby exhibiting heightened selectivity and specificity compared to small molecule drugs, consequently reducing the likelihood of off-target adverse reactions.^44^ Second, the degradation products of peptides in the body are amino acids, thereby diminishing the likelihood of systemic toxicity.^45^ Thirdly, peptides seldom ever accumulate in tissues because of their brief half-life.^46^ Due to its limited range (300–1000 A^2), small molecule drugs are difficult to effectively inhibit major biomolecular surface contacts, including protein-protein interactions (PPIs, contact area 1500–3000 A^2). Consequently, small molecule drugs encounter the challenge of effectively engaging key contact regions, thereby resulting in unintended off-target effects.^47,48^ Peptides possessing greater molecular dimensions and increased conformational flexibility relative to small molecule drugs may offer a solution to the challenge. Monoclonal antibodies are also a class of PPI inhibitors. Peptides exhibit greater cellular uptake and affinity for intracellular receptors compared to monoclonal antibodies, thereby enhancing their potential for biological activity. Our group conducted a review of peptide drug studies focused on targeting PPIs, including MDM2/p53, Keap1/Nrf2, and PD-1/PD-L1.^49^

Because of their instability in vivo and inadequate capacity to cross cell membranes, peptides present difficulties when being used in clinical settings. The main reason for this is that peptides have a lot of amino and carboxyl groups, which are difficult for them to pass lipid-based membrane structures since they frequently exhibit hydrophilicity, strong hydrogen bonding capacity, and low lipophilicity.^50,51^ In addition, due to their limited stability in the body, peptides are rapidly degraded by digestive enzymes in the gastrointestinal tract. As a result, they are removed from circulation within minutes. Large-scale protein hydrolysis and/or quick clearance in the liver, kidneys, or blood are the primary causes of this phenomena. It is worth noting that, with few exceptions (such as cyclosporine A), the bioavailability of most peptides following oral administration is less than 1%.^52^ This is primarily due to enzymatic degradation and pH-mediated hydrolysis in the gastrointestinal tract and liver, leading to low absorption rates and high first-pass effect.^53^ Consequently, commercially available peptides are primarily delivered subcutaneously, limiting the feasibility of more compliant oral delivery.^54^

The successful translation of peptide therapeutic candidates is dependent on high bioavailability and biodistribution, which include absorption and transport across biological membranes and cellular barriers.^55^ Solubility, lipophilicity, hydrogen bonding, chemical stability, and metabolic stability are all factors that influence these traits. Thus, peptide optimization is required, and chemical optimization procedures for therapeutic peptides are based on studies of structure-activity relationships (SAR) and/or quantitative structure-activity relationships (QSAR) of newly synthesized peptides. The goal is to boost bioavailability, minimize elimination and biodegradation, and improve selectivity or affinity for receptors or targets. Furthermore, technological improvements stimulate the creation of new delivery systems, which provide effective peptide translation.

### Bibliometric and visual analysis (2005–2024)

**Fig. 3:**
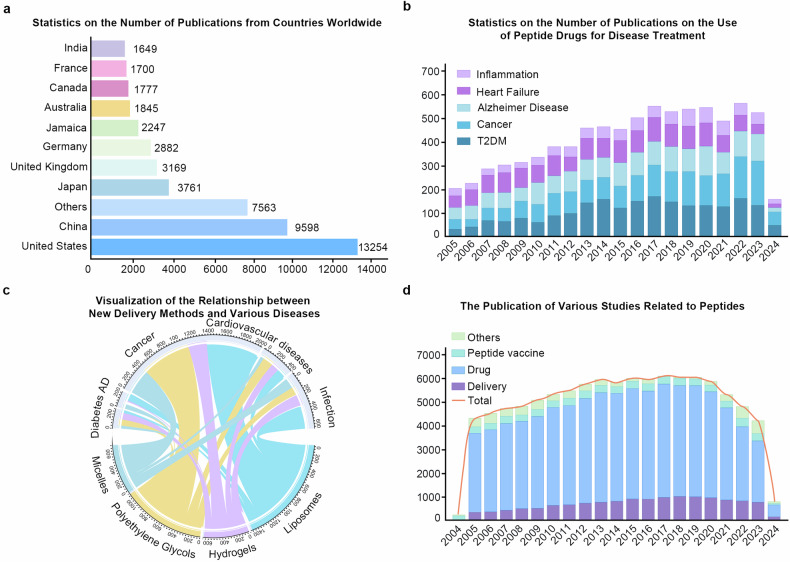
Analyses of literature on peptide drug published from 2005 to 2024. **a** Statistics of articles published by countries on drugs related to peptides. Among them, the United States had the highest number of publications, followed by China. **b** Statistics of the number of articles on peptide drugs for different diseases in the past 20 years. Over time, cancer has received increasing attention in peptide drugs. **c** New delivery methods for peptide drugs and visualization of the relationship between disease. As one of the most studied delivery methods, liposomes play an important role in the application of cardiovascular diseases. **d** Statistics of articles related to peptide drugs in the past 20 years. Among them, peptide vaccines have been widely valued by researchers

Here, we present an analysis based on a systematic and comprehensive literature search conducted on the PubMed database (Fig. 3). We used a series of key terms to formulate our search strategy, including “peptide”, “therapeutics”, “delivery systems”, “peptide vaccines”, and “drugs”, combined using the Boolean operators AND and OR. To refine our study, we utilized Medical Subject Headings (MeSH) terms and Boolean operators. Specifically, we searched for articles containing the term “peptide” either as a MeSH term or in the title/abstract, and also containing the terms “therapeutics”, “delivery”, “drug”, or “peptide vaccine”. We limited our search to articles published between 2005 and 2024. To further identify relevant articles, we analyzed the references of the selected papers and applied search filters to display publications within the specified timeframe only.

The final dataset comprised 87611 articles spanning 28 countries/regions. From these data, it’s evident that there is a sustained growth trend in the number of research articles focusing on peptides. The observed continued increase in the number of peptide-based articles reflects the increasing interest and research investment in peptides, particularly in 2019, when the number of articles published peaked, possibly owing to the milestone timing of the introduction of oral semaglutide.^56^ Over the period from 2005 to 2024, the United States contributed the highest number of publications, followed by China (Fig. 3a).

Key subject headings play a crucial role in summarizing the main ideas and core content of the literature.^57^ By visualizing the co-occurrence of key words, researchers can obtain a concise overview of research trends and potential future directions, thus providing valuable insights. Among the keywords identified between 2005 and 2024, the most common one is “drug delivery system”, followed by “insulin” and “natriuretic peptide”. Firstly, in order to overcome the characteristics of peptide drugs that are easily hydrolyzed by digestive enzymes in the human gastrointestinal tract,^58^ the development and update of peptide drug delivery systems have been a research hotspot for such drugs. Secondly, as two classic peptides with proven uses, insulin and natriuretic peptides are of great significance and have broad innovation prospects for the treatment of diabetes and cardiovascular diseases. Therefore, the development of peptide drug delivery methods focused on diabetes and cardiovascular diseases can visually represent the development of peptide drugs in the past 20 years.

In 2019, marketing approval was granted for oral semaglutide, the world’s first glucagon-like peptide-1 receptor agonist (GLP-1RA) approved for the treatment of type 2 diabetes, providing a promising new treatment option for people with diabetes. This milestone event undoubtedly brings hope to diabetes patients. In addition to their success in the field of diabetes treatment, peptides have also shown potential in cancer treatment. Based on the search results from 2020 to 2024, the frequency of the keyword ‘cancer’ has surpassed that of type 2 diabetes mellitus (T2DM) as the most relevant disease in this field (Fig. 3b). Therefore, peptides have been widely explored and applied in cancer therapy. On the one hand, peptides can directly kill tumour cells; on the other hand, as tumour-targeting peptides or peptide vaccines, they can enhance the therapeutic effect.

Based on the results of a search for peptide-based drug delivery systems, it is clear that oral, subcutaneous and intravenous administration have been the most commonly used modes of drug delivery over the past two decades. From the point of view of drug dosage form improvement, liposomes are the most popular, followed by hydrogels, polyethylene glycol and microgels. All these indicate that with the advancement of material science, the general direction of peptide drug delivery system research is to enable oral administration of peptide drugs by improving different drug carriers. Cross-tabulation of the search results showed that most of the novel drug delivery systems were developed for cancer diseases, probably because of the higher targeting and lower immunogenicity of these new drugs (Fig. 3c). In addition, the development of anti-infective and cardiovascular peptide drug delivery systems is also very popular, which is closely related to the high specificity and in vivo activity of peptides.

In summary, since the advent of insulin nearly a century ago, nearly a hundred peptide drugs have been approved for the treatment of a variety of diseases, including fatal diseases such as cancer and human immunodeficiency virus infection. The development of peptide-based drugs themselves has received increasing attention compared to the development of delivery modes and vaccines (Fig. 3d). Nowadays, more research is devoted to optimizing and improving these drugs to overcome the defects caused by their physicochemical properties and other factors. As an innovative treatment option, peptides are widely used in a variety of diseases, bringing hope and light to patients. In addition, peptides as a drug delivery and treatment strategy have different value in different drug delivery systems. Although published and ongoing peptide-based therapeutic research has generated encouraging data, it is still a difficult task to translate these results into clinical success due to species differences, production technology, cost and other factors.

### Peptide-based drug market & clinical trial

**Fig. 4:**
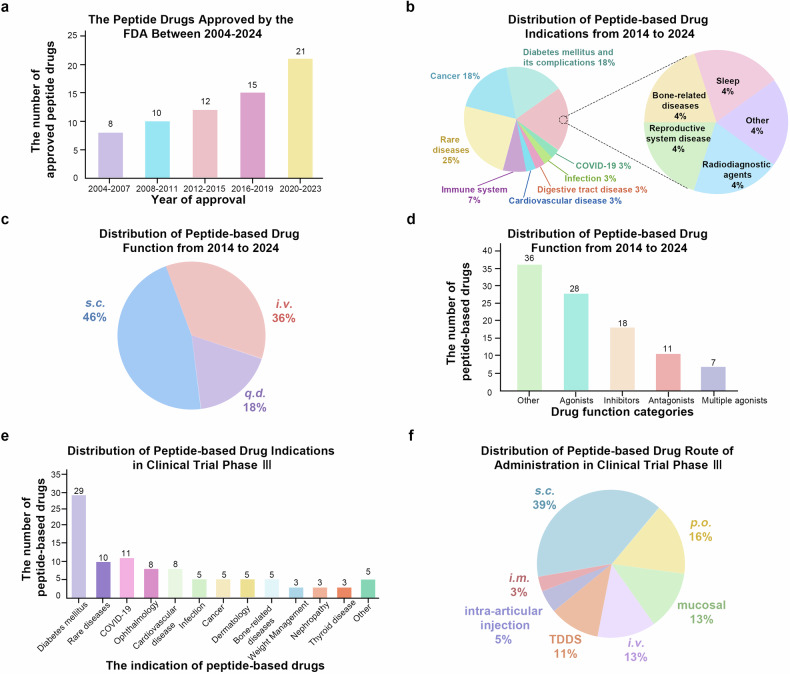
Peptide drugs market and clinical trials analysis. **a** Distribution of peptide-based drugs approval times from 2004 to 2024. Over the past two decades, the number of peptide drugs approved by the FDA has varied significantly across different time periods, with the highest number of approvals occurring between 2020 and 2023. **b** Distribution of peptide-based drugs indication from 2014 to 2024. The largest proportion targets rare diseases, followed by cancer and diabetes mellitus along with its complications. **c** Distribution of peptide-based drugs route of administration from 2014 to 2024. Subcutaneous injection remains the most prevalent method. **d** Distribution of peptide-based drugs function from 2014 to 2024. A diverse array of therapeutic roles, including metabolic regulation and hormone modulation. **e** Distribution of peptide-based drugs indication in clinical trial phase III. peptide drugs related to diabetes mellitus constitute the majority of indications. **f** Distribution of peptide-based drugs route of administration in clinical trial phase III. The variety of administration routes in Phase III clinical trials is extensive; however, subcutaneous injection continues to dominate as the preferred method of delivery

Over the past two decades, the number of approved peptide drugs has surpassed 60, with more drugs undergoing global approval processes (Fig. 4a). Based on the currently approved peptide drugs, the majority belong to the agonist category, and the most commonly targeted indications are associated with endocrinology, metabolism, and oncology (Fig. 4b–d). According to a research report by The Business Research Company, sales in the peptide market are projected to grow from $41.44 billion in 2023 to $45.66 billion in 2024, at a compound annual growth rate (CAGR) of 10.2%. The therapeutic peptide market is expected to experience rapid growth in the coming years. By 2028, this figure is forecasted to increase to $68.83 billion, with a compound annual growth rate (CAGR) of 10.8%. In 2023, North America emerged as the largest region in the therapeutic peptide market, while the Asia-Pacific region is anticipated to demonstrate the fastest growth during the forecast period. One contributing factor to this growth may be the rising prevalence of chronic diseases, which is driving the demand for peptide therapies. From a US-based agency, in January 2023, in individuals aged 50 and above, the number of people with chronic diseases in 2020 is projected to double by 2050.^59^ Peptide therapies are instrumental in both preventing and treating certain chronic diseases, thus the anticipated increase in the prevalence of chronic diseases is expected to drive the development of the peptide therapy market during the forecast period. As evidenced by sales statistics for the first half of 2023 (Table 1), semaglutide continued to lead global sales of peptide drugs in the first half of 2023 ($90.217 billion). Following closely behind was the GLP-1 RA dulaglutide ($37.896 billion), with the top 8 drugs by sales all used for diabetes treatment. Carfilzomib, a proteasome inhibitor used for the treatment of relapsed or refractory multiple myeloma, ranked 9th. Romiplostim, used for the treatment of immune thrombocytopenia, ranked 10th. Moreover, statistical data on the U.S. pharmaceutical expenditure market in 2023 revealed that semaglutide ($38.6 billion) is the new top overall drug among the top 25 highest-spending medications. This underscores the indispensable role peptide drugs play in the pharmaceutical market.^60^

**Table 1:** Top 10 selling peptide-based drug in 2023 H1

No.	Name	Brand Names	Indication	Company
1	Semaglutide	Ozempic	Diabetes	Novo Nordisk
2	Dulaglutide	Trulitciy	Diabetes	Eli Lilly
3	Semaglutide	Wegovy	Weight	Novo Nordisk
4	Tirzepatide	Mounjaro	Diabetes	Eli Lilly
5	Semaglutide	Rybelsus	Diabetes	Novo Nordisk
6	Insulin	NovoRapid	Diabetes	Novo Nordisk
	Aspart			
7	Insulin	Humalog	Diabetes	Eli Lilly
	Lispro			
8	Liraglutide	Saxenda	Weight	Novo Nordisk
9	Insulin	Lantus	Diabetes	Sanofi
	Glargine			
10	Carfilzomib	Kyprolis	Multiple myeloma	AMGEN

In 2023, an analysis of the peptide market share revealed that the metabolic system dominated the market with a revenue share of 37.80%. This underscores the significant importance of peptide therapy in the treatment of diabetes that over 1.31 billion people will have diabetes.^59^ Additionally, an estimated 44.7% of adults are unaware of their diabetic condition.^61^ On the other hand, the global objective is to curb the increase in diabetes and obesity by 2025, indicating a continued growth in global demand for such drugs. With advancements in new theories, technologies, and materials, the development of peptide drugs has undergone a historic transformation in the post-21st century, becoming more efficient. This has resulted in an increase in the number of approvals and clinical trials of peptide drugs, not only for diabetes but also for various other indications. Literature searches from 2020 to 2024 suggest that tumor research has surpassed diabetes as a key area for peptide drug development (Fig. 3).

From 2014 to 2024, a total of 28 drugs will be approved for marketing, indications for ranking the top three include rare diseases (7/29). Diabetes mellitus and its complications (5/29) and cancer (5/29). The functions of these drugs are mainly distributed as multiple agonists (2/29), agonists (8/29), inhibitors (5/29) and antagonists (3/29) (Table 2, Fig. 4a–d). Currently, 38 peptide drugs are in phase III clinical trials and are expected to enter the market. In addition to traditional diabetes and cancer treatments, the direction of peptide-based drugs discovery is clustered in COVID-19 (4/42), ophthalmology (3/42), weight management (1/42) and so on (Table 3, Figure 4e). The analysis of the route of administration revealed an interesting phenomenon. Routes of administration of approved peptide-based drugs are predominantly *s.c*. (13/29), remainder is *i.v*. (10/29) and *p.o*. (5/29). Significant increase in routes of drug administration in drug in phase III clinics. TDDS (4/42) as well as transmucosal drug delivery (5/42) were used to deliver peptide-based drugs (Figure 4f). The development of new materials and technologies facilitates the selection of more suitable routes of administration for peptide-based drugs. Due to their high specificity and ability to mimic or manipulate natural interactions between biomolecules, peptide drugs have become an important source of innovation in drug discovery, especially as new drug targets continue to emerge. In the future, advances in drug design and synthesis technologies, as well as innovations in drug delivery systems, are expected to improve the stability and bioavailability of peptide drugs, prolong their half-life in the body, reduce dosing frequency and improve patient compliance. We strongly believe that peptide drugs will usher in a safer, more precise and more convenient medical experience.

**Table 2:** Peptide-based drug approved by FDA from 2014 to 2024

No.	Name	Approval time	Mechanism of Action Targets	Indications Route of administration	Company
1	Albiglutide Tanzeum^®^	2014	GLP-1 receptor agonist Glucagon-like peptide 1 receptor	T2DM *s.c*.	GLAXOSMITHKLINE LLC
2	Dalbavancin Dalvance^®^	2014	Bacterial cell wall synthesis inhibitor	Acute bacterial skin and skin structure infection (absssi) *i.v*.	Allergan
3	Oritavancin Orbactiv^®^	2014	Inhibit transglycosylation	Skin and skin-structure infections *i.v*.	Melinta Therapeutics
4	Vasopressin Vasostrict^®^	2014	Vasopressin V2 receptor agonist	Vasodilatory shock *i.v*.	Par Sterile Products
5	Dulaglutide Trulicity^®^	2014	GLP-1 receptor agonist Glucagon-like peptide 1 receptor	T2DM *s.c*.	ELI LILLY AND CO
6	Parathyroid Hormone Natpara^®^	2015	Parathyroid hormone 2 (PTH 2) receptor agonist	Hypoparathyroidism *s.c*.	NPS Pharms Inc.
7	Grazoprevir Zepatier^®^	2016	NS3/4 A viral protease inhibitor	Chronic hepatitis c genotype 1 *p.o*.	Merck Sharpe & Dohme
8	Gallium dotatate Ga-68 Netspot^®^	2016	Binds to somatostatin receptors	Localization of somatostatin receptor positive neuroendocrine tumors (NETs) in adult and pediatric patients *i.v*.	AAA USA Inc.
9	Lixisenatid Adlyxin^®^	2016	GLP-1 receptor agonist	T2DM *s.c*.	Sanofi-Aventis
10	Macimorelin Macrilen^®^	2017	Growth hormone secretagogue receptor type 1 agonist	Adult growth hormone deficiency *p.o*.	Novo Nordisk
11	Voxilaprevir Vosevi^®^	2017	NS3/4 A viral protease inhibitor	Hepatitis C infections *p.o*.	Gilead Sciences Inc.
12	Etelcalcetide PARSABIV^®^	2017	Calcimimetic agent Extracellular calcium-sensing receptor	Secondary hyperparathyroidism (HPT) *i.v*.	KAI PHARMS INC
13	Semaglutide OZEMPIC^®^	2017	GLP-1 receptor agonist Glucagon-like peptide 1 receptor	T2DM *s.c*.	NOVO
14	Abaloparatide Tymlos^®^	2017	osteoanabolic agent Parathyroid hormone/parathyroid hormone-related peptide receptor	Osteoporosis *s.c*.	RADIUS
15	Plecanatide Trulance^®^	2017	Guanylate cyclase-C agonist Guanylate cyclase soluble subunit alpha-2	Chronic idiopathic constipation *p.o*.	SALIX
16	Angiotensin II Giapreza^®^	2017	vasoconstrictor Type-1 angiotensin II receptor	Low blood pressure *i.v*.	LA JOLLA PHARMA
17	Ivosidenib Tibsovo^®^	2018	Isocitrate dehydrogenase-1 inhibitor	Acute myeloid leukemia (aml) *p.o*.	Servier
18	Lutetium Lu 177 dotatate Lutathera^®^	2018	Radioligand Therapeutic Agent Somatostatin receptor	Treatment of somatostatin receptor-positive gastroenteropancreatic neuroendocrine tumors (GEP-NETs) *i.v*.	AAA USA INC
19	Afamelanotide SCENESSE^®^	2019	Melanocortin 1 Receptor (MC1-R) Agonists Melanocyte-stimulating hormone receptor	Erythropoietic protoporphyria *s.c*.	CLIVUNEL INC
20	Bremelanotide Vyleesi^®^	2019	agonist of melanocortin receptors Melanocyte-stimulating hormone receptor/Adrenocorticotropic hormone receptor	Hypoactive sexual desire disorder *s.c*.	PALATIN TECHNOLOGIES
21	Gallium ga-68 gozetotide Gallium Ga-68 PSMA-11^®^	2019	Radioligand Therapeutic Agent Somatostatin receptor	Radiodiagnostic agents for growth inhibitor receptor-positive neuroendocrine tumors *i.v*.	UIHC PET IMAGING
22	Gallium Ga-68 gozetotide Gallium Ga-68 gozetotide^®^	2020	Binds to prostate-specific membrane antigen (PSMA)	Prostate-specific membrane antigen positive tumors *i.v*.	University of California, Los Angeles
23	Copper Dotatate Cu-64 Detectnet^®^	2020	Binds to somatostatin receptors with highest affinity for subtype 2 receptors (SSTR2)	Somatostatin receptor positive neuroendocrine tumours i.v.	Radiomedix
24	Setmelanotide Imcivree^®^	2020	Melanocortin 4 Receptor Agonists melanocortin 4 (MC4)	Rare genetic obesity *s.c*.	RHYTHM
25	Voclosporin Lupkynis^®^	2021	CaN inhibitors (calcineurin inhibitors) CaN calcineurin	In combination with a background immunosuppressive therapy regimen for the treatment of adult patients with active lupus nephritis *p.o*.	Aurinia
26	Melphalan Flufenamide Pepaxto^®^	2021	DNA inhibitors DNA	Multiple myeloma *i.v*.	Oncopeptides AB
27	Lonapegsomatropin-TCGD Skytrofa®	2021	Release somatotropin, growth hormone receptor agonist	Growth failure *s.c*.	Ascendis Pharma
28	Odevixibat Bylvay®	2021	Ileal sodium/bile acid transporter (IBAT) inhibitor	Cholestatic pruritus *p.o*.	Albireo
29	Dasiglucagon Zegalogue^®^	2021	GCGR agonists (glucagon receptor agonists) GCGR	The treatment of severe hypoglycemia in pediatric and adult patients with diabetes aged 6 years and above *s.c*.	Zealand Pharma
30	Pegcetacoplan Empaveli ^®^	2021	C3 inhibitor C3	Treatment of adult patients with paroxysmal nocturnal hemoglobinuria (PNH) *s.c*.	Apellis Pharms
31	Difelikefalin Korsuva^®^	2021	κ opioid receptor κ opioid	Treatment of moderate-to-severe pruritus associated with chronic kidney disease (CKD-aP) in adults undergoing hemodialysis (HD) *i.v*.	CaraTherapeutics
32	Vosoritide Voxzogo^®^	2021	NPRB agonist NPRB	Chondrodysplasia in children *s.c*.	Biomarin Pharm
33	Daridorexant Quviviq^®^	2022	OX1R antagonist (orexin receptor 1 antagonist) OX2R antagonist (orexin receptor 2 antagonist) OX1R &OX2R	The treatment of adult patients with insomnia, characterized by difficulties with sleep onset and/or sleep maintenance *p.o*.	IDORSIA
34	Lutetium 177Lu vipivotide tetraxetan Pluvicto^®^	2022	Radioligand Therapeutic Agent PSMA prostate cancer-specific membrane antigen	Metastatic denervation-resistant prostate cancer *i.v*.	AAA USA NOVARTIS
35	Tirzepatide Zepbound^®^	2022	GIP and GLP-1 dual agonists GIPR&GLP-1R	T2DM *s.c*.	Eli Lilly & Co.
36	Terlipressin Terlivaz^®^	2022	AVPR1A agonist AVPR1B agonist AVPR2 agonist AVPR1A&AVPR1B &AVPR2	Improvement of renal function in adult patients with hepatorenal syndrome (HRS, hepatorenal syndrome) with rapidly declining renal function *i.v*.	MALLINCKRODT IRELAND
37	Rezafungin Rezzayo^®^	2023	1,3-β-Glucan synthase inhibitor 1,3-beta-glucan synthase	Treatment of candidemia and invasive candidiasisin patients 18 years of age and older who have limited or no alternative options *i.v*.	Cidara Therapeutics
38	nirmatrelvir and ritonavir Paxlovid^®^	2023	Protease inhibitors: CYP3A inhibitors (cytochrome P450 family member 3A family inhibitors) SARS-CoV 3CL^pro^ inhibitor (SARS coronavirus 2-3C-like protease inhibitor) CYP3A & SARS-CoV-2 3CL^pro^	Treatment of COVID-19in adults who are at high risk for progression to severe COVID-19, including hospitalization or death *p.o*.	PFIZER
39	Trofinetide Daybue^®^	2023	IGF-1R agonist Insulin-like growth factor-I receptor (IGF-1R)	Treatment of Rett syndrome in adults and pediatric patients 2 years of age and olde *p.o*.	ACADIA PHARMS INC
40	Flotufolastat F 18 Posluma^®^	2023	PSMA Modulator (Prostate Cancer Specific Membrane Antigen Modulator), PET imaging (positron emission tomography enhancement) PSMA (Prostate Cancer Specific Membrane Antigen), visualized by binding to PSMA-expressing cells (e.g. prostate cancer cells).	Prostate cancer *i.v*.	Blue Earth Diagnostics
41	Motixafortide Aphexda^®^	2023	CXCR4 antagonist CXCR4	First innovative drug targeting stem cell mobilization in multiple myeloma *s.c*.	BioLineRx
42	Zilucoplan Zilbrysq^®^	2023	CXCR4 antagonist gMG targets C5 complement	Adult patients with generalized myasthenia gravis (gMG) who are positive for anti-acetylcholine receptor (AChR) antibodies *s.c*.	UCB INC

**Table 3:** Peptide-based drug in clinical phase III

R&D Code	Name	Target	Indications Route of administration	Clinical Trial ID
ACT-1	Granexin^738^	GJA1	Diabetic Foot Ulcers (DFUs) Topical-gelling agent	NCT02667327
CJC-1134-PC	Albenatide^739^	GLP-1R	T2DM *s.c*.	CTR20202681
-	Ambervin	IL-6	COVID-19 *i.m*.	NCT05656495
FE 203799	Apraglutide^740^	GLP-2 R	Short Bowel Syndrome (SBS) *s.c*.	NCT05018286
-	BRM-421^741^	BRM-421	Dry Eye Syndrome (DES) eye drops	NCT05695781
AM-833	Cagrilintide^742^	CGRP R	T2DM *s.c*.	NCT05669755
TD-1792	Cefilavancin^743^	Antibiotic	Bacterium infection *i.v*.	NA
DD-04107	DD-04107^248^	synaptotagmin-1	Neuropathic pain Topical lotion, s.c.	EUCTR2022-001374-60-ES
XW-003	Ecnoglutide^744^	GLP-R	Weight management *s.c*.	NCT05813795
XW-003	Ecnoglutide^744^	Thyroid Hormone Receptor	Type 2 diabetes *s.c*.	NCT05680155
AZP-3601	Eneboparatide^745^	PTH-1R	Hypoparathyroidism *s.c*.	NCT05778071
MK-0616	Elicitide chloride^218^	PCSK9	Arteriosclerosis *p.o*.	NCT06008756
FE-106483 VA-106483	Fedovapagon^746^	V2R	Nocturia *p.o*.	NCT02637960
ZP-1848	Glepaglutide^747^	GLP-R	Short bowel syndrome *s.c*.	EUCTR2020-005502-25-NL
NN1535	Insulin icodec^748^	INSR	T2DM *s.c*.	NCT05259033
JTA-004	JTA-004^749^	unknown	Osteoarthritis Intra-articular injection	EUCTR2019-000796-16-CZ
AZP-531	Livoletide^750^	ghrelin	Prader-willi syndrome *s.c*.	EUCTR2018-003062-13-NL
LP-17/LR-12/LR-17/TREM-1 inhibitors	Nangibotide^751^	TREM1	COVID-19, Virus identified *i.v*.	EUCTR2020-001504-42-ES
NA-1	Nerinetide^752^	PSD-95	Acute ischemic stroke *i.v*.	EUCTR2020-002360-30-NL
MSI-78	Pexiganan^753^	Antibiotic	Type2 diabetes Emulsions	NCT01594762
PL-5	Peceleganan^429^	Antibiotic	Diabetic foot ulcer Aerosol	CTR2300071255
BIM-28131 BIM-28163	Relamorelin^754^	Ghrelin Receptor/GHSR	Diabetic gastroparesis *s.c*.	EUCTR2017-002177-20-RO
P2K PENIEL 2K、PENIEL-2000	Remedisc^755^	TGF-β1	Degenerative disc disease injection	NCT05516992
LY 3437943	Retatrutide^756^	Glucagon R	T2DM *s.c*.	NCT05929079
rE-4	Exenatide-4^757^	GLP-1R	Type2 Diabetes *s.c*.	NCT03239119
PTG-300	Rusfertide^758^	Hepc	Anemia Polycythaemia vera *s.c*.	NCT06033586
SNP ACTH (1-39) SNP-ACTH	SNP-ACTH9 (1-39)^759^	ACTH receptor	Idiopathic membranous nephropathy Primary Membranous Nephropathy *s.c*.	NCT05696613
-	SR-0379^760^	Akt-1\Class I-PI3K\ mTOR	Skin ulcer Transdermal drug delivery	JRCT2031210266
CB-183315 CB-315	Surotomycin^418^	unknown	Infection Clostridium difficile infection *p.o*.	EUCTR2012-000252-34-AT
BI-456906	Survodutide^761^	Glucagon R	T2DM *s.c*.	NCT06066528
MIM-D3	Tavilermide^762^	Trk	Dry eye syndromes Eye drops	NCT05848128
RGN-259	Timbetasin^763^	thymosin beta 4	Dry eye syndrome Eye drops	NCT05555589
NA 831	Traneurocin^764^	GR	COVID-19 Virus identified *p.o*.	NCT04540185
AMG 386	Trebananib^765^	ANGPT1/ANGPT2	Ovarian cancer *i.v*.	NCT01281254
ANF-95-126 ANP 95126 ANP-95126	Ularitide^766^	NPR1	Decompensated heart failure *i.v*.	EUCTR2006-002403-13-FI
AT-406 DEBIO-1143	Xevinapant^767^	IAP	Head and Neck Cancer p.o.	NCT05386550
YKYY-017	YKYY-017^468^	SARS-COV-2 S protein	COVID-19 Virus identified Nebulized inhalation	CTR2300075467
PN-2123555 PN-235	JNJ-2113	IL-23R	Plaque Psoriasis *p.o*.	NCT06220604

## Advance in peptide-based delivery platforms

The cell membrane serves as a crucial physiological barrier that impedes the passage of therapeutic molecules to their intended target site. CPPs represent a viable approach for enhancing the intracellular uptake of therapeutic molecules, thereby enabling the exertion of therapeutic effects. CPPs facilitate the delivery of various cargoes, including nanocarriers, drugs, and nucleic acids.^62–64^ CPP-based peptide delivery platforms have emerged as a significant focus of research, and we have summarized the advancements made in their clinical trials, indications primarily include cancer, cardiovascular disease, and imaging (Table 4). This chapter examines the advancements in CPP-engineered nanocarriers and research on CPP conjugate drugs.

**Table 4:** CPPs-based delivery platforms in clinical phase

No.	Name	CPPs	Cargo	Indications	Clinical Trial ID Status
1	P28^768^	P28	NA	Solid tumors	NCT00914914 Phase I
2	P28^768^	P28	Non-HDM2-mediated peptide ihibitor of P53	CNS malignancies	NCT01975116 Phase I
3	AM-111^769^	TAT	JBD20 (D-JNKI-1)	Hearing loss	NCT02809118 Phase III
4	KAI-9803^770^	TAT	δPKC inhibitor	Myocardial infarction	NCT00785954 Phase II
5	KAI-1678^771^	TAT	Εpkc inhibitor	Postherpetic neuralgia postherpetic neuralgia spinal cord injury postoperative	NCT01106716 Phase II
6	AVB-620^772^	ACPPs	Cy5 and Cy7	Tumor imaging	NCT02391194 Phase I
7	XG-102^773^	TAT	JBD20 (D-JNKI-1)	Postoperative ocular inflammation	NCT02235272 Phase III
8	DTS-108^774^	a highly charged oligopeptide of human origin	SN38	Tumour	NA Phase I
9	ALRN-6924^775,776^	a cell-penetrating stapled alpha-helical peptide structure undisclosed	Palbociclib	Solid tumour	NCT02264613 Phase II
10	ALRN-6924^775,776^	a cell-penetrating stapled alpha-helical peptide structure undisclosed	Cytarabine	Acute myeloid leukemia and advanced myelodysplastic syndrome	NCT0909972 Phase I
11	ALRN-6924^775,776^	a cell-penetrating stapled alpha-helical peptide structure undisclosed	Paclitaxel	Advanced, metastatic or unresectable solid tumors	NCT0725436 Phase I
12	PsorBan^777^	R7	cyclosporin A	Psoriasis	NA Phase IIb discontinued 2003
13	AZX100^778,779^	PTD4	HSP20 phosphopeptide	Scar prevention/ reduction	NCT00892723 Phase II
14	RT001^780,781^	MTSsss	Botulinum toxin A	Lateral canthal Line Crow’s feet Facial wrinkles	NCT01940991 Phase II
15	RT002^782^	TransMTS	botulinum toxin A	Glabellar lines	NCT02303002 Phase I/II
16	NA	TransMTS	Daxibotu-linumtoxin A	Cervical dystonia	NCT02706795 Phase II
17	AVI-4658^783^	NA	Duchenne muscular dystrophy	NA	NCT02255552 Phase III
18	AVI-5126^784,785^	(R-Ahx-R)_4_	Phosphorodia-midate morpholino oligomers (PMOs)	Cardiovascular disease Coronary artery bypass	NCT00451256 Phase II
19	AEM-28^786^	a high-affinity lipid-associating peptide (DWLKAFYDKVAEKLKEAF)	An arginine-rich apo E receptor bind-ing domain (residues 141-150 LRKLRKRL-LR)	Type II hyperlipoproteinemia	NCT02100839 Phase I/II
20	PEP-010^787,788^	NA	Interfering peptide	Metastatic solid tumor cancer	NCT04733027 Phase I

### Peptide-drug conjugates

#### Introduction of peptide-drug conjugates (PDCs)

In cancer treatment, the inability to distinguish tumor cells from normal cells has led to substantial side effects with current drugs. An innovative solution is Antibody-Drug Conjugates (ADCs) - monoclonal antibodies linked to cytotoxic drugs.^65^ ADCs enhance targeting and reduce side effects. However, ADCs have limitations like complex pharmacokinetics, side effects, and ineffective drug release.^66^ To address these issues, PDCs have emerged as a novel approach. Though PDCs and ADCs share conceptual similarities as targeted antitumor therapies, they differ markedly in structure and pharmacological behavior.^67^

Unlike bulky ADCs, PDCs feature short CTPs instead of antibodies, enabling specific binding to receptors overexpressed on cancer cells.^68,69^ Owing to their low molecular weight, typically just a few kilodaltons, PDCs demonstrate superior tumor penetration and lower immunogenicity compared to ADCs, which generally exceed 150 kilodaltons.^63^ While ADCs utilize hepatic metabolism, PDCs employ renal clearance. The conformational freedom of PDCs’ abbreviated peptide sequences also permits the introduction of non-natural amino acids. This facilitates cyclic peptide formation and chemical conjugation to other molecules, expanding PDCs’ targeting potential, stability, and versatility.^70,71^ With enhanced precision, durability, and adaptability compared to earlier generations of ADCs, PDCs have emerged as highly promising next-generation targeted cancer therapies.^63,67^

The composition of PDCs involves three components: 1) the cell-targeting peptide, 2) a linker, and 3) the cytotoxic payload.^63^ CTPs are selected peptides that can specifically target protein receptors overexpressed on tumor tissues, allowing drug delivery vehicles like PDCs to be directed to target cells.^69,72,73^ These peptides often have high binding affinity, in the nanomole range, to their receptors.^27^ Their binding is affected by their secondary structure, which must be retained when attaching a linker.^74^ Common cell-targeting examples bind integrins and CD13 receptors on tumors, like the RGD and NGR peptides.^75,76^ CTPs can be linear or cyclic/stapled, with cyclic versions having improved stability and binding, like a cyclic RGD peptide targeting glioblastomas.^63,75^ Some CTPs act as CPPs, transporting compounds into cells, with cationic ones having less specificity than anionic and may produce unwanted toxicity to normal cells at higher peptide concentrations.^77^ Among various CPPs, arginine-rich peptides are the most widely used. Recently, it is reported that the specific cell penetration, toxicity, and in vivo biocompatibility of CPPs can be balanced by designing peptides with optimal arginine residue lengths, enlightening us that perhaps CPPs contain other possibilities of PDCs.^78^ Besides, advances in technologies such as proteomics, phage display, and mRNA display have enabled the discovery and design of a growing repertoire of targeted peptides, propelling PDC development.^79,80^

Linkers are used to connect the cell-targeting peptide to the cytotoxic drug payload. Ideal linkers are stable in circulation to prevent premature drug release and toxicity.^74^ Cleavable linkers release drug via chemical (e.g. acid, glutathione) or enzyme triggers in the tumor environment.^81^ Non-cleavable linkers rely on carrier breakdown to release an intact drug-linker complex to kill cells.^82^ Cleavable linkers allow more controlled drug release but non-cleavable linkers can have increased stability.^27,83^ The choice of linker depends on the desired design and mechanism of action of the targeted therapeutic. For instance, oxime-linked anthracycline-peptide conjugates have demonstrated many advantages in PDCs development, including ease of direct synthesis; the conjugation reaction proceeds in excellent yields; and the autofluorescence of anthracyclines provides a basis for selecting appropriate ones.^84^ Besides, it is worth noting that stimuli-responsive linkers can also improve cell-targeting peptide targeting.^85^

Cytotoxic drug payloads used in PDCs often have limitations like poor pharmacokinetics when used alone. Attaching them to targeting peptides helps overcome issues and enhances their therapeutic window.^86^ Desired drug payloads have high potency with IC_50_ values in the nanomolar range, stability in circulation, and a point for attaching a linker.^68^ Examples include doxorubicin (DOX), paclitaxel (PTX), and mertansine.^87^ Radionuclides like ^177^Lu-dotatate are also increasingly used as payloads, enabled by peptide targeting. Radionuclides are repurposed FDA-approved agents that allow imaging to precisely locate tumors and monitor progression by PET or SPECT.^88^ The high selectivity of peptides for receptors gives excellent tumor contrast with minimal off-target effects. Useful radionuclides include positron emitters like ^68^Ga and ^18^F for PET scanning,^89^ and gamma emitters like ^123^I and ^99m^Tc for SPECT.^90^ Bifunctional chelators like DOTA and DTPA link radioisotopes to targeting vectors.^91^ Overall, the versatility of PDCs allows targeted delivery of diverse cytotoxic and radionuclide payloads.

Up to now, several PDCs have entered clinical evaluation (Table 5), with two receiving regulatory approval—Lutathera (Lutetium Lu-177 Dotatate) in 2017 and Pepaxto (melphalan flufenamide) in 2021.^92,93^ Novartis’ Lutathera utilizes a somatostatin-targeting peptide and the radionuclide ^177^Lu to treat gastrointestinal and pancreatic neuroendocrine tumors.^94^ Oncopeptides developed Pepaxto, comprising alkylating agent-conjugated peptides targeting aminopeptidase for relapsed/refractory multiple myeloma, though it was later withdrawn from the U.S. market due to disappointing efficacy.^95^ In addition to the approved PDCs discussed above, two of the most advanced PDCs under development are SNG1005 and AEZS-108. SNG1005 is a brain-targeted PDC that has demonstrated promising clinical efficacy in treating brain metastases from breast cancer, and is currently in Phase 3 clinical trials.^96^ AEZS-108 is a PDC that is being evaluated in trials for the treatment of proliferative fibrotic prostate cancer.^96,97^

**Table 5:** Peptide-drug conjugates drug in clinical trial

No.	Name	CTPs	Drugs	Linker	Indications	Clinical Trial ID Status
1	ANG1005^789^ (paclitaxel trevatide)	Angiopep-2	Paclitaxel	Succinic- acid	Leptomeningeal metastases	NCT03613181 Phase III
2	GRN1005^790^	Angiopep-2	Paclitaxel	Succinic- acid	Breast cancer brain metastases; non-small cell lung cancer (nsclc) with brain metastases	NCT01679743 Phase II
3	BT1718^791^ (Bicycle Therapeutics)	MT1-MMP binder	Mertansine (DM1)	Disulfide	Advanced solid tumours;non-small cell lung cancer;non-small cell lung sarcoma	NCT03486730 Phase I/II
4	BT5528^792^	EphA2 binder	MMAE	Amide	Solid tumors EphA2-positive NSCLC	NCT04180371 Phase I
5	BT8009^118^ (Bicycle Therapeutics)	Nectin-4- binder	MMAE	Amide	Solid tumors	NCT04180371 Phase I/II
6	TH1902^793^	TH19P01	Docetaxel	Succinic acid	Solid tumors	NCT04561362 Phase I
7	TH1904^794^	TH19P01	Doxorubicin	Succinic acid	Solid tumors	NCT04706962 Phase I
8	G-202^795^ (mipsagargin)	DγEγEγEγE	Thapsigargin	Amide	Solid tumors	NCT02381236 Phase II
9	NGR015^796^	CNGRCG (1,5SS)/RGD	hTNF	Amide	Malignant pleural mesothelioma	NCT01098266 Phase III
10	Ttf-ngr^797^	GNGRAHA/NGR	Ttf	Amide	Malignant solid tumors lymphomas	NCT02902237 (2020) Phase I
11	PEN-221^798^	fCYwKTCC (2,7 SS)	DM-1	Disulfide	Neuroendocrine tumors carcinoma Small cell lung cancer	NCT02936323 (2021) Phase I/II
12	Zoptarelin doxorubicin^799^	D-Lys6-LHRH	Doxorubicin	Amide	Pre-treated advanced/metastatic recurrent endometrial cancer	NCT01767155 (2020) Phase III
13	CBP-1008^800^	CB-20BK	MMAE	Amide	Advanced solid tumor	NCT04740398 (2022) Phase I
14	CBP-1018^671^	LDC10B	MMAE	Amide	Lung tumor	NCT04928612 (2022) Phase I
15	SOR-C13^801^	folate	MMAE	Amide	Advanced malignant solid neoplasm	NCT03784677 (2021) Phase I
16	[18 F]AlF-NOTA-octreotide^802^	octreotide/RGD	18 F	NOTA	PET or GEP-NETs Neuroendocrine tumors	NCT04552847 (2020)/NCT03883776 (2020) Phase I/II/III
17	[18 F]Fluciclatide^800^	RGD	18 F	PEG	PET imaging	NCT00918281 (2014) Phase II
18	[18 F]RGD-K5^160^	cyclo (RGDfK)	18 F	NOTA	PET imaging	NCT03364270 (2020) Phase II
19	68Ga-NODAGA-E [cyclo (RGDyK)]2^803^	E [cyclo (RGDyK)]2	68 Ga	NODAGA	PET imaging	NCT03271281 (2021) Phase II
20	68Ga-NOTA-BBN-RGD^75^	cyclo (RGDyK) and BBN	68 Ga	NOTA	PET imaging	NCT02747290 (2016) Phase I
21	90Y-DOTATOC^804^	3Tyr-octreotate /DOTATOC	90Y	DOTA	PRRT	NCT03273712 (2019) Phase II
22	99mTc-3PRGD2^805^	3Tyr-octreotate/RGD	99mTc	3PRGD2	Breast cancer	NCT02723760 Phase I
23	111In-DTPA-octreotide^806^	3Tyr-octreotate	111In	DTPA	Brain and central nervous system Tumors PET imaging	NCT00002947 (2014) Phase I
24	CBX-12^807^ (Cybrexa Therapeutics) (alphalex-exatecan)	a pH-sensitive peptide	Exatecan		Advanced solidtumors	NCT05691517 Phase l/ll
25	OPD5^808^ (Oncopeptides AB)		Melflufen	an aminopeptidase-targeting linkage	Relapsed multiplemyeloma	NCT04918511 Phase I
26	TH1902^809^ (Theratechnologies)	TH19P01	Docetaxel/sudocetaxel	a cleavable ester	Triple-negativebreast cancer	NCT04706962 Phase I
27	phosphorodiamidate morpholino oligomer-cell-penetrating peptide^810^ (EDO-51、PGN-EDO23、PGN-EDO51)		Oligonucleot-ide		Duchenne dystrophy	NCT06079736 Phase II
28	bevonescein^811^ (ALM-488)	RGD	Fluorescein		Head and neck injury	NCT05377554 Phase III
29	EP-100^812^ (onvitrelin ucalontide)	CLIP-71	LHRH	none	Solid tumors	NCT01485848 Phase II
30	BGC-0228^96^	CD44	TOP1 inhibitor		Advanced solid tumor	CTR20220194 Phase l
31	SC-101^813^	Nectin-4 binder	microtubule inhibitors		Advanced solid tumor	NCT06220838 Phase l
32	AEZS-108/ZEN-008/AN-152^814^	[d-Lys6] LHRH	doxorubicin	a glutaric acid spacer	Endometrial Cancer	NCT01767155 Phase III

#### Current research status: unveiling diverse peptide-drug conjugates

As the next generation of smart drug conjugates, the impending wave of PDCs brings new hope for targeted therapy, especially in the field of anti-tumor.^27,67^ In the sections below, we detail the various PDCs targeting tumors that have been developed so far, categorized by the targets targeted by their cell-targeting peptide moieties.

##### Integrin-targeting PDCs

Integrin-Targeting PDCs have emerged as an innovative therapeutic strategy, especially those targeting the α_v_β_3_ integrin subtype.^98^ Recognizing the pivotal roles of integrins, particularly their widespread overexpression across numerous cancer types, researchers have tapped into their potential for targeted drug delivery using PDCs.^99^ RGD peptides have taken center stage owing to their remarkable binding specificity for α_v_β_3_ integrins.^100^ Strategically integrated into PDCs as guiding entities for cytotoxic payloads like DOX and camptothecin (CPT), RGD peptides enable enhanced therapeutic effects by precisely targeting cancer cells with elevated α_v_β_3_ expression.^75^ Further complementing PDCs design sophistication are innovative linkers, a cleverly designed example utilizes albumin metabolism to mediate apoptosis and trigger the expression of Caspase-3, which allows the PDC to remain persistently activated, enabling payload accumulation and subsequent bystander killing effects. As caspase-3 becomes continuously upregulated within the tumor, on-site cellular apoptosis is exacerbated. The sustained caspase-3 upregulation, mediated by albumin metabolism, enables continuous PDC activation and payload buildup, leads to enhanced bystander effects and further promotes in situ tumor cell apoptosis.^101^

Furthermore, α_v_β_6_ integrin-targeting PDCs, specific to pancreatic ductal adenocarcinomas, hold promise in treating pancreatic ductal adenocarcinomas (PDAC).^102^ Taken SG3299 as an example, formed by conjugated the DNA-binding pyrrolobenzodiazepine (PBD) payload SG3249 (tesirine) was to an α_v_β_6_-specific 20mer peptide from foot-and-mouth disease virus (FMDV) VP1 capsid protein, this α_v_β_6_-targeted PDC SG3299 demonstrated significantly greater toxicity (up to 78-fold) against α_v_β_6_-expressing cells compared to the non-targeted PDC SG3511 at equimolar doses. In in vivo xenograft studies, SG3299 eliminated established (100 mm^3^) Capan-1 PDAC human xenografts and significantly prolonged mouse survival,^103^ exemplifying the precision and potential therapeutic impact of α_v_β_6_-directed PDCs for pancreatic cancer.

##### EGFR-targeting PDCs

Epidermal Growth Factor Receptor (EGFR) is a pivotal transmembrane receptor governing cancer cell signaling, represents an attractive molecular target for cutting-edge precision therapeutics.^104^ The innovative development of EGFR-targeting PDCs is strategically designed to leverage the overexpression of the EGFR in diverse cancer types. To enhance cellular uptake and facilitate unique photoactivated cytotoxic effects, both linear and cyclic EGFR-binding peptides are incorporated to selectively targeting EGFR-dysregulated cancers for more effective treatment.^67^ For example, a linear EGFR-binding peptide (YHWYGYTPEVI) conjugated with phthalocyanine, achieving heightened uptake and photoactivated cytotoxicity against epidermoid carcinoma cells.^105^ Besides, a cyclic EGFR-binding peptide (CMYIEALDKYAC) elicited preferential accumulation in EGFR-overexpressing malignancies, which in turn produced significantly higher photocytotoxicity and effectively inhibited tumor growth after photodynamic therapy.^106^ Recently, to develop targeted therapies for cancers with overexpression of EGFR due to kinase domain mutations like L858R/T790M that promote cancer cell growth, studies have explored novel PDCs of WNWKV and LARFFS binding to wildtype and mutant EGFR. In lung cancer cells expressing wildtype or double mutant EGFR, WNWKV conjugates showed greater anticancer potency than LARFFS conjugates, while unconjugated LARFFS displayed poor binding. The enhanced antiproliferative effects of WNWKV conjugates highlight their potential for targeting EGFR mutants like L858R/T790M, providing guidance for developing therapies against EGFR receptors.^107^

##### HER2-Targeting PDCs

Capitalizing on HER2 receptor overexpression in cancers like breast and lung, HER2-targeting PDCs have also emerged as an impactful focus.^108^ For example, a Cyclo-GCGPep1 peptide was modeled after the trastuzumab-HER2 interface and conjugated it with CPT, creating a PDC with potent antiproliferative effects on HER2^+^ cells. Additionally, a novel HER2 PDC was developed by conjugating a homodimer HER2-targeting peptide (composed of two identical binding ligands connected via a linker) with the cytotoxic agent DOX using an acid-cleavable hydrazone linker. The PDC exhibited significantly enhanced tumor-targeting capabilities and anticancer activities, particularly against HER2^+^ tumors. Meanwhile, the dimerization of the HER2-binding peptide ligand led to increased receptor affinity and cellular internalization compared to the monomeric counterpart.^109^ Similar examples include a novel PDC obtained by fusing the N-terminal sequence of the second mitochondria-derived activator of caspases (SMAC) with P1, followed by conjugation to a CPT molecule. Which exhibited outstanding in vitro antitumor activity against three HER2-positive cell lines, comparable to CPT alone.^110^ To harness the HER2 axis, innovative computer-designed bispecific fusion peptides with superior HER2^+^ binding has also been reported.^67^

##### D) TfR-targeting PDCs

Transferrin Receptor (TfR)-targeting PDCs present another avenue for selective drug delivery by targeting the overexpressed TfR in cancer cells.^111^ For instance, a recently reported PDC was designed and synthesized by using N-succinimidyl-3-maleimidopropionate (SMP) as a crosslinker to connect the TfR targeting peptide analogue BP9a (CAHLHNRS) and DOX. Which exploits BP9a mediated TfR recognition for selective delivery of doxorubicin to malignant cells and enhances therapeutic index and mitigate off-target toxicity to normal tissues.^112^ In addition, the development of a novel T7-SN-38 PDC using the affinity of HAIYPRH (T7) peptide to TfR overexpressed on blood-brain barrier and glioma cells has also been recently reported.^113^

##### E) SORT1-targeting PDCs

Recent advancements spotlight the SORT1 receptor as a promising target that with predominant overexpression observed across numerous malignant tumor types.^114^ By exploiting the SORT1 internalization function, a peptide (TH19P01) was developed. In vitro, although the TH19P01 peptide itself exerted no antiproliferative or apoptotic effects when tested on TNBC-derived MDA-MB-231 cells, the docetaxel-TH19P01 conjugate (TH1902) demonstrated potent antiproliferative and antimigratory activities.^115^

##### VEGFR-Targeting PDCs

VEGFR-targeting PDCs, adopting an antiangiogenic approach, couple VEGFR-targeting peptides with lytic peptides, showing potential toxicity to hepatoma cells.^116^ For instance, by conjugating a VEGFR-targeting peptide (QKRKRKKSRYKS) to a lytic peptide (KLUKLUKKLUKLUK), not only can it target and inhibit VEGFR on the cell surface to effectively suppress angiogenesis in tumor tissues, but it has also demonstrated superior in vivo antitumor efficacy compared to the traditional drug DOX in a VX2 rabbit tumor model with a favorable safety profile.^117^ Besides, this tailored PDC exhibited potential hepatoma cell toxicity, showcasing enhanced anticancer efficacy through specific VEGFR targeting.

##### Diverse targeting strategies

Furthermore, CTPs continue to diversify beyond conventional receptors, with emerging efforts directed against unconventional antigens like poliovirus receptor-related protein 4 (Nectin-4),^118^ somatostatin receptor 2 (SSTR2),^119^ gonadotropin releasing hormone receptor (GnRHR), transient receptor potential vanilloid subfamily member 6 (TPRV6),^120^ glypican-3 (GPC3),^121^ E-selectin,^122^ melanocortin-1-receptor (MC1R),^123^ receptor tyrosine kinase-like orphan receptor 1 (RORl),^124^ Kita-Kyushu lung cancer antigen 1 (KK-LC-1),^125^ and Hsp90.^126^ For instance, two of the most advanced PDCs under development are SNG1005 and AEZS-108. SNG1005 is a brain-targeted PDC that has demonstrated promising clinical efficacy in treating brain metastases from breast cancer, and is currently in Phase 3 clinical trials.^96^ AEZS-108 is a PDC targeting GnRHR, which is being evaluated in trials for the treatment of proliferative fibrotic prostate cancer.^96,97^ Besides, an ingenious design is an intelligent ultrasound theranostic peptide-porphyrin conjugate (P18-P) that self-assembles into supramolecular structures through cathepsin B (CTSB)-triggered aggregation. After intravenous injection, the multifunctional probe achieves deep tissue penetration due to the penetration sequence of P18-P. More importantly, CTSB-triggered self-assembly greatly extended the retention time, amplifying photoacoustic imaging signals for sensitive CTSB detection, and enhanced sonodynamic therapy activity for oxygen generation, eliciting a specific CTSB-responsive ultrasound therapy.^127^ As representatives, these PDCs showcase the versatility of PDC research in addressing specific molecular markers and paving the way for novel therapeutic interventions across various cancer types.

#### Future prospects of peptide-drug conjugates

PDCs have emerged as a transformative and precise targeted cancer treatment that combines the selectivity of peptides with the cytotoxicity of chemotherapeutic agents, effectively addressing the limitations associated with traditional chemotherapy, expanding the therapeutic window, enhancing efficacy, and redefining targeted therapy.^27,63,67,69,128^

Despite notable success in clinical use, challenges such as poor stability and a short half-life persist. Looking ahead, the future of PDCs involves the development of PDCs based on humanized antibodies,^129^ exploring stimuli-responsive drug release mechanisms,^130^ employing computer-aided drug design,^131^ exploring diverse receptors or antigens,^132^ integrating with immunotherapies and gene therapies to address stability, off-target effects and resistance issues,^133,134^ and advancing scalable manufacturing processes.^135^

### CPPs-engineered nanocarriers

The challenge of delivering molecularly targeted therapies to specific regions with precision is a common obstacle in the commercialization of new drugs. Peptides, with their high receptor affinity, low toxicity, design flexibility, and cost-effectiveness, have made peptide-based drug delivery systems a popular area of research.^136^ CPPs, also known as protein translocation domains (PTDs), membrane translocation sequences, or Trojan peptides, are a group of cationic peptides consisting of 5-30 residues.^137,138^ In 1988, Frankel purified the first CPP from a virus with the sequence YGRKKRRQRRR. This peptide exhibited efficient intracellular translocation and was named HIV Tat (transactivating) protein (TAT).^139^ TAT was used to create delivery systems for small molecule, nucleic acid and immunotherapy drugs.^140–143^ Due to technological advancements, a growing number of naturally derived or synthetically produced CPPs have been introduced into the research field.^137^ Currently, over 100 CPPs have been identified or synthesized and are structurally categorized as cationic, cyclic, amphipathic, or hydrophobic. CPPs are classified based on their source as follows: (1) naturally occurring protein-derived CPPs, such as penetratin (Pen) and Tat; (2) chimeric CPPs, such as transportan, which is a peptide composed of 14 amino acids from the venom of the yellowjacket wasp (Vespula lewisii) and 12 amino acids from the N-terminus of substance P; and (3) fully synthetic CPPs, such as oligoarginines and peptide nucleic acids (PNAs).^144,145^

**Fig. 5:**
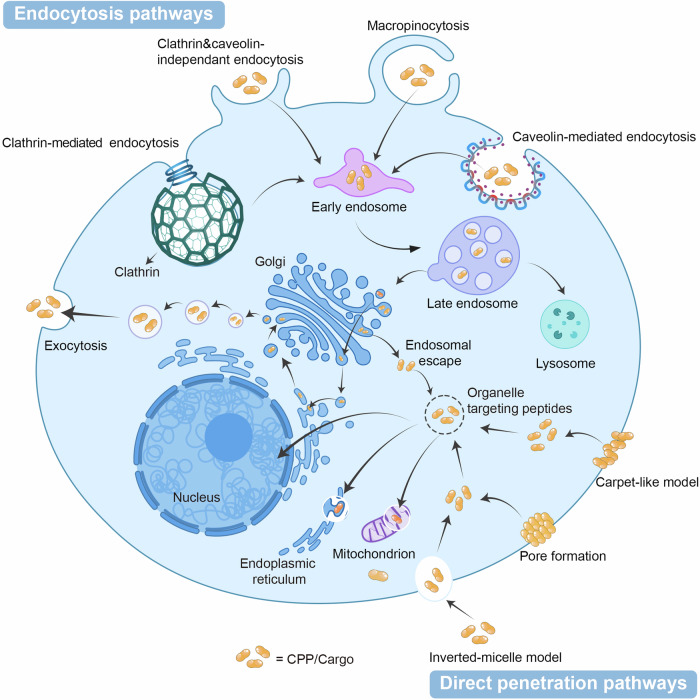
Overview of physiological incorporation of peptide-based delivery strategies (including targeting, transmembrane, escape, organelles). There are two major pathways for the incorporation of CPPs including endocytosis pathways and direct penetration pathways. Through endocytosis, CPPs induce membrane concavity, resulting in the formation of endosomes within the cell, while certain CPPs trigger the development of large, irregular macropinocytosomes. In contrast, direct penetration occurs through three distinct mechanisms: First, CPPs can translocate through substantial holes in the lipid bilayer, where the cell membrane forms an extensive pore channel. Alternatively, CPPs may cross the membrane via transient prepores, characterized by smaller-diameter, short-lived openings in the cell membrane. Finally, CPPs can traverse the lipid bilayers through the formation of inverted micelles, offering yet another pathway for cellular entry

Just as the efficacy of drugs within the body depends on their ability to cross physiological and pathological barriers, such as the phospholipid bilayer, the key to the action of CPPs lies in their transport efficiency.^146^ CPPs can facilitate cargo internalization by directly conjugating with the C-terminus of proteins or peptides, creating nanocomplexes through hydrophobic/electrostatic interactions, or covalently linking with nucleic acid molecules.^147^ However, the specific mechanisms of internalization are still not well understood. Cellular parameters, cargo characteristics, and CPP physicochemical qualities all impact internalization efficiency.^148^ Internalization involves two steps: overcoming the membrane barrier and exiting the endosomes. Two membrane translocation processes are thought to be energy-independent direct translocation and energy-dependent endocytosis. The interaction between cationic CPPs and negatively charged membrane structures, such as glycosaminoglycans, can lead to the formation of pores, inverted micelles, and a ‘carpet model’ during direct translocation.^149–151^ CPPs, which typically contain arginine and lysine residues, carry a positive charge at physiological pH, enabling electrostatic interactions with negatively charged cell membranes or cargo. Among them, arginine-containing CPPs such as TAT and octa-arginine (R8) have gained a lot of interest.^152^ Endocytosis, an energy-dependent process, can be achieved through various mechanisms, including clathrin-dependent endocytosis, lipid raft-mediated endocytosis, and caveolin-mediated endocytosis. Kaplan et al. reported that TAT is internalized via lipid raft-mediated endocytosis,^153^ which is influenced by cell membrane features, microenvironmental factors, and cargo characteristics.^154,155^ Following cellular uptake, cargo must escape the endosome to exert therapeutic effects, as seen with TAT.^156^ The internalization and the endosomal release mediated by CPPs is influenced by multiple mechanisms and may vary in different pathological and physiological environments, leading to ongoing debates regarding its specific processes (Fig. 5).

**Fig. 6:**
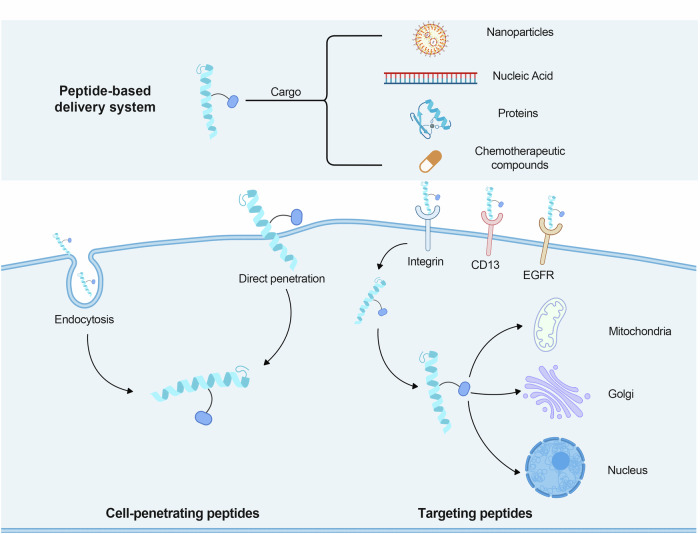
Overview of Targeting and Delivery Strategies for Peptide-based Delivery Systems. CPPs enable the efficient translocation of cargo molecules (e.g., drugs, therapeutic proteins, or nucleic acids) across cellular membranes, facilitating their intracellular delivery and therapeutic action within target cells. The another is Targeting peptides. These peptides can specifically recognize various disease-associated targets expressed on the surface of target cells or in the diseased tissue environment. Only a few typical examples of such targets are depicted, serving as illustrations for the diverse range of potential targets that can be exploited by targeting peptides for disease-specific applications. Figure 6 was created with biorender.com

To date, FDA has not approved a single CPP or CPP/drug combination, likely due to the inherent challenge of CPPs solely facilitating the accumulation of transmembrane cargo at specific therapeutic target sites. Homing/targeting peptides have become a major avenue of research in peptide-based drug delivery systems to address the need for tumor targeting together with the CPPs (Fig. 6). Unlike naturally occurring CPPs, homing/targeting peptides are often identified through display library screening,^157,158^ with further advances expected to yield more potential homing/targeting peptides. Tumor-homing peptides (HTPs) offer several benefits, including biocompatibility, low cytotoxicity to non-tumor cells, low immunogenicity, and high permeability due to their small size, which form the peptide-based drug delivery system. Additionally, they can be easily modified and redesigned as needed.^159^ Delivery systems that have been altered to include homing/targeting peptides have demonstrated impressive outcomes in immunotherapy, radiation, chemotherapy, and photo/photodynamic therapy.^84,160–166^ Several targeting peptides have been developed to bind to receptors that are highly expressed on the surface of tumor cells, including integrins, HER2, and EGFR. Examples of these peptides include the HER2-targeting peptide AHNP,^167^ the integrin-targeting peptide iRGD,^168^ and the EGFR-targeting peptide GE11.^169^ Recent research has also identified a novel CPPs called T3BP. The potential exists for it to bind to the type III transforming growth factor-β receptor (TGFBR3) and provide a novel treatment approach for tumors such as breast, prostate, and colon cancers.^170^ See the excellent description of homing peptides and representative CPPs by Eisaku Kondo’s team.^171^ We believe that with the assistance of new technologies, homing peptides represent a promising new tool in biomedicine.

Our paper provides an update on the development of research into targeting specific intracellular organelle peptides. Drug delivery to particular subcellular organelles, such as the Golgi, endoplasmic reticulum,^172^ mitochondrial,^173^ lysosomal,^174^ and nuclear transport,^175^ is facilitated by the peptides, which are important in the delivery of drugs, genes, and other relevant fields. In order to transfer VEGF siRNA and ASO into colorectal cancer cells, for example, a study described the construction of multifunctional CPP and nuclear localization signal (NLS) cyclic peptide CSP2 (Cyclo [WWWWGGRRRRGC]).^176^ The authors previously published a study on a nanostructure based on a tryptophan and arginine-repeated cyclic peptide (Cyclo [WWWWGGRRRGG]).Another study employed a simple enzyme replacement therapy using poly-arginine and poly-histidine peptides as carriers, successfully facilitating the delivery of lysosomal enzyme α-galactosidase A to lysosomes, offering new insights into the treatment of Fabry disease.^174^

New technologies offer opportunities for cardiac targeting and overcoming the blood-brain barrier, which is a challenge for traditional CPPs. Cardiac-targeting peptides are an exciting area of research due to the global prevalence of cardiovascular disorders.^177^ Ideally, cardiac drugs should exhibit cardiac selectivity. The Zahidt team used the M13 bacteriophage display library to screen and discover a potential cardiac-targeting peptides with high cardiomyocyte targeting.^178^ At the same time, a comprehensive report on the discovery and implementation of cardiac-targeting peptides has been created,^179^ which is useful in this industry. The blood-brain barrier (BBB) is a vital natural barrier in the body and is linked to several neurological illnesses. Overcoming the blood-brain barrier to achieve brain-targeted molecular delivery is often a primary challenge faced by many researchers in drug design. In recent years, Nose-to-Brain (N-to-B) delivery has emerged as a novel method for drug delivery system. It offers a non-invasive alternative route for the delivery of macromolecules and serves as a convenient approach for rapid targeting of the central nervous system, bypassing the blood-brain barrier and minimizing systemic exposure. Mentzer and colleagues developed a CGRP inhibitor peptide (34 Pro, 35 Phe) CGRP 27–37, which was then prepared as chitosan microparticles for intranasal delivery in dry powder form. This method prevented peptide degradation and provided some relief for migraines.^180^ Another significant approach involves using nanocarriers conjugated with peptides for brain targeting. Nanocarriers can recognize transcytosis receptors on the blood-brain barrier to achieve effective drug delivery.^181^ For example, modified PLGA-NPs exhibited enhanced brain uptake and provided better MRI contrast for diagnostic purposes.^182^ A hybrid metal nanocarrier system consisting of gold and superparamagnetic iron oxide nanoparticles, coated with carboxymethyl cellulose, has been developed. This system can induce oxidative stress and magnetic hyperthermia upon exposure to alternating magnetic fields when functionalized with integrin-binding peptide (iRDG), offering potential for targeted therapy of brain cancer.^183^

## Advance of peptide-based therapeutics

### Enhancing peptide stability and bioavailability through structural modifications

#### Introduction to structural modification

**Fig. 7:**
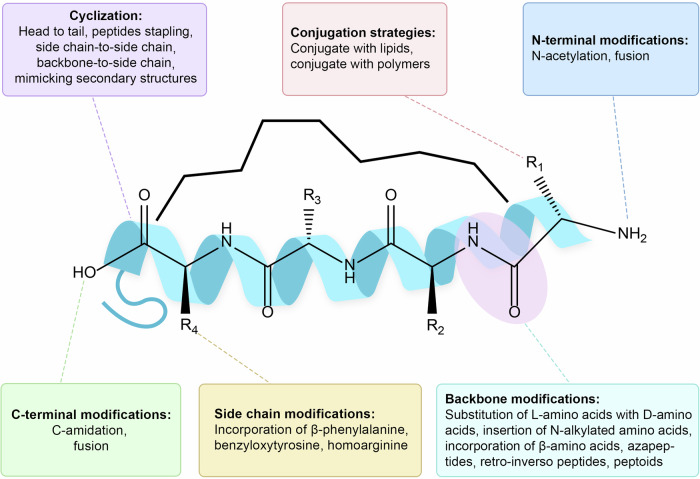
Strategies to improve the physicochemical properties of peptide drugs, including cyclization, conjugation strategies, N-terminal modifications, C-terminal modifications, side chain modifications and backbone modifications

Peptide therapeutics, owing to their natural amino acid-based composition, exhibit inherent advantages. However, despite these advantages, peptides face substantial challenges, primarily related to their structural properties.^184^ Generally, peptides have two major limitations as therapeutics: poor membrane permeability and instability in vivo.^2^ Their size and amino acid composition hinder crossing cell membranes to reach intracellular targets.^185^ Moreover, the lack of secondary and tertiary structures makes peptides susceptible to enzymatic degradation. Additionally, their amide bonds are also prone to hydrolysis. These two factors result in short half-lives and rapid elimination in vivo for peptides.^184^ These limitations, coupled with the high manufacturing costs associated with peptide drug discovery, create hurdles in their widespread application. To overcome these limitations, structural modifications have emerged as a key strategy. A variety of modification methods (Fig. 7) have been widely applied to enhance stability against enzymatic cleavage, improve bioavailability by overcoming biological barriers, and fine-tune specificity for selective interactions with target molecules.^184^

#### Structural modifications for stability and improved bioavailability

Overcoming the instability against proteolysis and limited bioavailability associated with peptide therapeutics are among the most pressing issues in the field of structural modifications. Various structural modification methods have been applied, and here we provide a brief overview of these approaches and their applications.

##### A) Backbone modifications

Backbone modifications are the earliest structural modification method in the field that targets the masking or removal of amide bonds in the peptide backbone. When it comes to this approach, the most commonly employed tactics include substituting D-amino acids for L-amino acids,^186,187^ inserting N-alkylated amino acids,^188^ and incorporating β-amino acids or α/β-substituted α‑amino acids.^189,190^ Each strategy is implemented through different principles to address proteolytic stability issues that often plague native peptides.^191^ Substituting metabolically labile L-amino acids with their D-amino acid counterparts enhances resistance to enzymatic degradation, significantly extending the peptide’s half-life.^186^ The insertion of N-alkylated amino acids and incorporation of β-amino acids or α/β-substituted α‑amino acids strategically fortify the peptide structure, leading to improved stability.^188^ In the process of synthesizing lanthipeptides, the backbone modifications are elegantly presented, for instance, d-amino acids are successful introduced into lanthipeptides via two enzymatic reactions catalyzed by the dehydratase domain of lanthipeptide synthase,^192^ showcasing the potency of backbone modifications in overcoming challenges associated with proteolytic instability in peptide therapeutics.

Replacing one or more peptide bonds with an isosteric or isoelectronic substitute are also strategies that modify the peptide’s backbones. The isosters that are most frequently used include azapeptides, retro-inverso peptides, and peptoids,^193–195^ bringing forth diverse modifications and offering unique functionalities to peptide drugs. Azapeptides have a similar structure to natural peptides, but with the key difference of having a nitrogen atom rather than a carbon atom bonded to the amino group, which makes azapeptides useful synthetic mimics of natural peptides. For instance, a powerful new covalent inhibitor of the SARS-CoV-2 main protease (M^pro^) using an azapeptide scaffold capped with a cysteine residue was reported recently, enabling targeted, irreversible labeling of the M^pro^ active site and makes it one of the most potent M^pro^ inhibitors reported so far.^196^ Retro-inverso peptides, with reversed N- and C-termini sequences and L- to D-amino acid substitutions, find applications as versatile immunomodulators, anti-inflammatory agents, and more.^197–199^ Peptoids, featuring N-alkylated glycines, provide flexibility and have applications in cancer, neurological and autoimmune disorders.^200^

##### B) Side chain modifications

Side chain modifications are another strategic maneuver in the enhancement of stability and bioavailability of peptide drugs. The principles underlying this approach involve the replacement of natural amino acids with their analogues during the synthesis of peptides. This substitution seeks to bring about advantages such as the augmentation of binding affinity and target selectivity.^201^ Notable applications of side chain modifications include the incorporation of analogues like β-phenylalanine,^202^ benzyloxytyrosine,^184^ and homoarginine.^203^ These modifications have found practical implementation in near-infrared (NIR) dye-peptide conjugates, for instance, it has been reported that the use of acetamidomethylcysteine to replace the cysteine residue in a near-infrared fluorescent dye conjugated with the type I collagen targeting peptide RRANAALKAGELYKCILY successfully disrupted the self-assembly of the peptide and thereby changed the performance of the molecular probe in aqueous solution, ultimately improving the contrast of arthritic joints against the background.^204^

##### C) Peptide cyclization

Among the six peptide drugs approved in 2023, three are cyclic peptide drugs (Rezafungin, Motixafortide and Zilucoplan), emphasizing that cyclic peptides have become an important modality in the development of peptide drugs.^205–207^ Delving into the peptide cyclization unfolds diverse approaches, most commonly used including head-to-tail, backbone-to-side chain, and side chain-to-side chain.^208^ The advantages stemming from peptide cyclization encompass heightened proteolytic stability,^209,210^ and the facilitation of secondary structure formation.^211,212^ These principles have been applied tangibly in the development of S-tert-butylation of the free thiol group of cysteine. Which was done in a rationally designed peptide using the evolutionarily conserved γ-core region (GXC-X3-9-C) of the antifungal protein from Aspergillus.^213^ S-tert-butylation not only improved stability, but also extended the peptide’s antifungal activity to the mold Aspergillus fumigatus.

A significant advantage of cyclic peptides lies in their pharmacokinetic properties. Specifically, their unique characteristics in stability, hydrophilic/lipophilic balance, cell permeability, etc. make cyclic peptides a key focus for the development of orally bioavailable peptide drugs.^214^ Among the currently marketed oral peptides, three are cyclic peptides - Cyclosporine A, Voclosporin, and Desmopressin. The favorable pharmacokinetics conferred by their cyclic structure contributes to their success as orally bioavailable peptide therapeutics.^215–217^ In addition to these marketed drugs, some recently reported cyclic peptide drugs also show potential. For instance, MK-0616 is an orally bioavailable, renally excreted cyclic peptide inhibitor of PCSK9. In clinical trials, MK-0616 has demonstrated dose-dependent reductions in LDL cholesterol, non-HDL cholesterol, and apolipoprotein B levels. Besides, it can lower Lp(a). MK-0616 is currently in Phase 3 clinical trials and shows promise for the treatment of cardiovascular diseases given its oral bioavailability, potent lipid-lowering effects, and renal clearance.^218^ Meanwhile, stapled peptides, which are also cyclic peptides in nature, skillfully “stapled” into α-helix and β-sheets shapes, exhibit proteolytic resistance and extended plasma half-life,^219^ exemplified by stapled peptides that resemble Helix 1 of the human ACE2 receptor, have shown varying degrees of efficacy in preventing SARS-CoV-2 infection.^220^ In addition, cyclized analogues adopted type I β-turn structures achieved by substituting select glycine residues with N-(2-thioethyl) glycine and stapling the peptides using bifunctional reagents was reported. Which yielded cyclic analogues with improved analgesic activity compared to the parent enkephalins after introducing benzyl substituents on the trithiocyanurate stapling reagents.^221^ Besides, stapling natural peptides by cross-linking two amino groups via different imidazolium linkers with various α-ketoaldehyde reagents has been reported recently,^222^ providing more possibilities for the design of stapled peptides.

Another frequent use of peptide cyclization is to stabilize various secondary structures like α-helixes and β-sheets, known as “mimicking secondary structures.”^2,223^ Mimicking secondary structures employing strategies like cross-linking or hydrogen bond surrogates to replicate α-helices and β-sheets.^2^ Key approaches for mimicking and stabilizing α-helices in peptides include lactam-based crosslinks, disulfide bonds, and biselectrophilic linkers. Lactam-based crosslinks form a lactam bridge by substituting lysine for the side chain of glutamic acid or aspartic acid, enhancing hydrogen bond formation and restricts conformational freedom of the peptide chain to mimic and stabilize alpha helical conformations.^224^ Disulfide bonds can form covalent connections between peptide chains when N-terminal serine residues are replaced with homocysteine or cysteine.^225^ Biselectrophilic linkers can react with two amino acid side chains on the peptide chains simultaneously to generate connections.^226,227^ To stabilize β-sheets, peptides are modified by introducing D-amino acids, a successful example is the recently reported use of mirror-image phage display technology to select D-amino acid peptide ligands for aggregation-prone proteins that play a role in neurodegenerative diseases, thereby avoiding the accumulation of endogenous proteins such as amyloid beta peptide (Aβ), achieving therapeutic purposes.^228^ As for β-sheet mimicking, there are also reports that have created β-sheet mimics via macrocyclization or amyloid.^229,230^ In conclusion, mimicking secondary structures enhances stability and specificity, enabling a dynamic shift in peptide therapeutic design, allows creating structures that closely mimic protein-protein interfaces, demonstrating the potential of secondary structure mimicry.

##### D) Conjugation strategies

Conjugating peptides to larger molecules are also a method that has become quite popular in recent years, which not only increase lipophilicity, but the enhanced steric hindrance also prevents filtration of the conjugate through the kidneys and prolongs circulation time. Units commonly used to conjugate include lipids and polymers. Lipid conjugation strategy entails tying peptides to lipids such as glycerides, steroids, and fatty acids.^231,232^ The principles behind this approach include forming stable ester or amide bonds, with fatty acids such as squalenoic acid and docosahexaenoic acid playing prominent roles.^233,234^ The advantages offered by lipid conjugation include reduced toxicity and improved bioavailability.^235^ An illustrative example involves the conjugation of the Poly (L-glutamic acid) (PGA), as the hydrophilic backbone, with the peptide antigen Ag and the molecular adjuvant imidazoquinoline (IMDQ) TLR7/8 agonist, respectively. The polyanionic properties of PGA were used to bind the electrostatic interaction of ionizable lipids condenses PGA-Ag and PGA-IMDQ into lipid nanoparticles.^236^

Conjugation with polymers is another useful tactic to increase the stability, prolong the in vivo half-life, and lessen the immunogenicity of peptides, with PEGylation being a widely used technique.^237^ This approach operates on the principles of extending half-life and mitigating immunogenicity, providing a shield against enzymatic degradation.^237^ For instance, FDA-approved PEGylated proteins Krystexxa and PEGASYS have elevated the standard for stability and in vivo efficacy of peptide therapeutics.^238^ Despite these gains, it has also been suggested that whereas conjugation shields targeting peptides from proteolysis to some extent, altering the peptide sequence to increase protease resistance can significantly increase homing and transport efficiency.^239^

##### E) Terminal modifications

As exopeptidases typically break down peptide sequences at either the N- or C-terminus, it is possible to increase a peptide’s resistance to protease hydrolysis by modifying its terminus. N-acetylation, C-amidation, and fusion with albumin are the main concepts when it comes to terminal alterations and fusion tactics.^188,240–242^ These modifications orchestrate an extended plasma half-life, amplifying the potential of peptide-based therapeutics. Exemplifying this, with site-specific albumin conjugation, a “clickable” non-natural amino acid named azide-l-phenylalanine (AzF) was added to three specific sites (V16, Y19, and F28) of the GLP-1 variant and then linked to HSA through a strain-promoted azide-alkyne cyclization reaction. The resulting three HSA-conjugated GLP-1 variants (GLP1_16HSA, GLP1_19HSA, and GLP1_28HSA) have serum half-lives comparable to HSA in vivo.^243^

#### Conclusion and future directions

The future of structural modification of peptide drugs is poised to witness several exciting advancements. First, precision targeting is gaining prominence, with researchers designing peptides that exhibit high specificity for their intended targets, enabling precision targeting of disease-related proteins or receptors.^98^ Additionally, we’re witnessing the emergence of multi-functional peptides that combine therapeutic effects with diagnostic capabilities or even serve as drug delivery vehicles.^244^ Another promising trend involves stapled peptides, which stabilize peptide structures through covalent bonds, are expected to see further advancements to enhance peptide stability and bioavailability.^184,219^ Besides, the exploration of novel peptidomimetics, synthetic molecules that mimic the properties of peptides, may help overcome limitations associated with natural peptides.^245,246^

However, the field faces several research challenges that require innovative strategies to address. Improving the oral bioavailability of peptides, which often struggle with enzymatic degradation and poor absorption, remains a critical priority.^247^ Prolonging the typically short half-lives of peptides without compromising their efficacy is another key challenge.^248^ Overcoming immunogenicity, where some peptides can trigger unwanted immune responses, is also crucial.^195^ As researchers explore more intricate peptide structures, the complexities of synthesis and characterization present additional hurdles.^184,249^

To address these challenges, various technologies and strategies are being explored. Backbone modifications alter the peptide backbone to enhance stability and resistance to enzymatic degradation. Side chain modifications, achieved by substituting specific amino acids with modified analogs, can improve properties like solubility and binding affinity, might find applications in tailored therapeutics for diverse diseases. Conjugation with polymers extends half-life and improves pharmacokinetics, may see refinements for specific drug delivery needs. Cyclization, creating cyclic peptides, enhances stability and reduces susceptibility to proteases. Terminal modifications and fusion strategies are likely to be refined, offering extended half-lives for peptide therapeutics. Lastly, novel delivery systems, such as nanoparticles or liposomes, enhance tissue penetration and overall bioavailability. By leveraging these innovative strategies, the future of peptide drug development holds great promise in precision, multifunctionality, and overcoming existing limitations.^250^

### Advance of peptide-based therapeutics in diabetes

Obesity is a chronic progressive disease that affects nearly 760 million adults worldwide. Obesity not only affects human health and quality of life, but also increases the risk of T2DM, and cardiovascular disease. Diabetes is a chronic metabolic disease that is prevalent globally, with 537 million people already living with diabetes as of 2021, and T2DM accounts for approximately 90% of all people with diabetes, a number that is expected to continue to increase in the coming decades.^251^ Diabetes imposes a huge burden on the health of individuals and society. In addition to obesity, complications such as diabetic cardiomyopathy, diabetic nephropathy, and diabetic foot can occur, increasing the rate of disability and death.^252^

T2DM is primarily caused by insulin resistance and insufficient insulin secretion, and usually develops in adults, especially middle-aged and older adults. Weight management can be used to control the onset and progression of the disease in both T2DM and obese patients. Weight management is a lifestyle-based treatment that combines a personalized low-calorie diet, physical activity, and behavioral counseling. This intervention is able to reduce patient weight moderately (5%-10%) and control the onset of T2DM, but further weight loss is needed for effective control and most patients treated with weight management have difficulty adhering to it.^253^ Although drugs such as exenatide and simethicone are now marketed for the treatment of diabetes, they are not yet sufficient, so the development of novel drugs remains important.^254,255^

#### History of peptide-based therapeutics in diabetes

Insulin, the prototypical pharmaceutical agent for the treatment of diabetes, was initially derived and purified from the pancreatic tissue of a canine in 1921 and subsequently validated in a 12-year-old adolescent in 1922. In 1923, Eli Lilly and Company’s insulin (Insulin^®^) was launched on the market; in the same year, the Nobel Prize in Medicine and Physiology was awarded to the discoverers of insulin, such as Banting and MacLeod. However, the initial production method was not only complicated, costly and low-yielding, but also susceptible to immune reactions and viral infections.^256^ With the advent of genetic engineering, researchers recombinantly obtained insulin in E. coli. Unlike extracted animal insulin, this type of insulin is attributed to human proteins, and the immune response is greatly controlled.^257^ In 1982, Insulin human^®^ was approved by the FDA as the first recombinant therapeutic protein.^258^ With the development of genetic and chemical engineering, the number of engineered insulins gradually increased and insulin modifications followed. 1996 saw the approval of Lispro^®^ (Humalog) as the first industrially engineered insulin. Detemir (Levemir^®^), which is a long-acting insulin, adds a fatty acid chain to the carboxyl terminus of the insulin β-chain.^259^ Monnier et al. successfully prolonged the duration of insulin’s action by acylating insulin at LysB29, which allowed this peptide hormone to bind with endogenous serum proteins. In recent years, researchers have partially electrostatically mediated under the skin by introducing mutations at the C-terminal end of the β-chain leading to the aggregation of mutant insulins, allowing them to be gradually secreted into the bloodstream.^260^ Many new insulin analogs have also been developed, such as the long-acting insulin analog Insulin glargine, which has a duration of action of up to 30 h, and the rapid-acting insulin analog insulin aspart, which has an immediate immediate onset of action after 10–15 min and peaks after 1–2 h (Table 6).^261^

**Table 6:** Pharmacokinetic data of commercially available insulins

	Insulin preparations	Onset of action	Peak time	Duration of action
Prandial insulin	Regular Insulin	30 min	1.5–2.5 h	5–8 h
	Insulin Aspart	10–15 min	60-90 min	4–5 h
	Insulin Lispro	10–15 min	1.0 h	4–5 h
Basal insulin	NPH	2.5–3.0 h	5–7 h	13–16 h
	PZI	3–4 h	8–10 h	≥ 20 h
	Insulin Glargine	1.1 h	No peak	≥ 24 h
	Insulin Detemir	3-4 h	No peak	24 h
	Insulin Degludec	1–2 h	No peak	24 h

In 1923, Charles Kimball and John Murlin found and identified glucagon. In the 1980s, Joel Habener^262^ described a new glucagon-related peptide encoded in the pre-glucagon cDNA of the pipefish. In the 1980s, Joel Habener described a new glucagon related peptide encoded in the pipefish pre-glucagon cDNA. Two glucagon related peptides were subsequently identified in rat, bovine, hamster and human glucagonogen. These two peptides are now referred to as GLP-1 and glucagon-like peptide-2 (GLP-2). GLP-2 is an enterotrophic hormone released by enteroendocrine cell (EEC) L cells,^263^ and its receptor is mainly distributed in the digestive tract, exerting enteroprotective effects through different pathways GLP-2 acutely prevents endotoxin-related increased intestinal paracellular permeability in rats. Currently, the field of diabetes is dominated by the study of GLP-1. GLP-1 has a wide range of pharmacological and therapeutic uses. In addition to its ability to reduce gastric emptying, inhibit food intake, or control metabolism, the activation of the GLP-1 receptor induces a protective effect, the regulation of the hypothalamic-pituitary-adrenal axis, heart and lungs. It also reduces the production of inflammatory cytokines, chemokines, and the infiltration of immune cells in tissues, resulting in a broad range of neuroprotective and anti-inflammatory effects. GLP-1 interacts with its receptors to stimulate insulin secretion from pancreatic β-cells and inhibit glucagon release from pancreatic α-cells, increasing satiety and delaying gastric emptying in a glucose-dependent manner. Endogenous GLP-1 is degraded and rapidly inactivated by dipeptidyl peptidase-4 (DPP-4). To prolong the stimulation of GLP-1 receptors, GLP-1 RAs need to be synthesized to prevent their degradation.

GLP-1 RAs are emerging drugs for glycemic control and have been widely used in the treatment of T2DM in recent years. Currently, GLP-1 RAs are mainly categorized into peptide and non-peptide. Based on the similarity of their amino acid sequences, peptide agonists are mainly categorized into GLP-1 and derivatives and exendin-4 and derivatives.^252^ In recent years, with the growing market size of the GLP-1 RA class of drugs, the U.S. glucose-lowering drug market has undergone a trend shift, evolving from insulin-based drugs as the star drugs before to GLP-1 drugs leading the way.

Since the FDA approved the first GLP-1 RA, Exenatide (Eli Lilly, Exenatide®), in 2005, six GLP-1 RAs have entered the clinic, including liraglutide (2010/2014/2016, Novo Nordisk A/S, Victoza^®^/Saxenda^®^/Xultophy^®^), lixisenatide (2016, Sanofi-Aventis, Lyxumia^®^/Adlyxin^®^/Soliqua^®^), albiglutide (2014, GSK Plc, Eperzan^®^/Tanzeum^®^), dulaglutide (2014, Eli Lilly, Trulicity^®^), semaglutide (2017, Novo Nordisk A/S, Ozempic^®^/Rybelsus^®^) and tirzepatide (2022/2023, Eli Lilly, Mounjaro^®^/Zepbound^®^), in addition to efpeglenatide and taspoglutide which are in clinical studies. When injected, these GLP-1 RAs help the pancreas release the right amount of insulin when blood glucose is high, effectively lowering glycated hemoglobin and average blood glucose levels and improving fasting glucose.

As the first GLP-1 RA that can be used orally, semaglutide is able to effectively control blood glucose levels and achieve appetite reduction and weight loss by slowing down gastric emptying through brain regions that regulate appetite and food intake.^264^ For patients with T2DM, the use of semaglutide has multiple implications. First, as a hypoglycemic drug, it can help patients effectively control their blood sugar levels and reduce the risk of stroke, heart attack, or death in patients with T2DM, as well as in patients with cardiovascular disease, and it is suitable for use in overweight/obese patients suffering from hyperglycemia, as well as, with a proper diet and exercise program. Secondly, semaglutide also has cardiovascular protective effects,^56^ which is certainly an important therapeutic option for diabetic patients with comorbid cardiovascular disease or at high risk of cardiovascular disease. In addition, Novo Nordisk has made three modifications to semaglutide: 1. alpha-aminoisobutyric acid has been used to replace alanine at position 8. 2. a C18 lipoic acid side chain has been linked to lysine at position 26, with glutamic acid as the linker. 3. arginine has been used to replace lysine at position 34. The above three modifications enable the ultra-long-lasting characteristics of semaglutide, which needs to be taken orally only once a week, which greatly improves the convenience of dosing and patient compliance.

In addition to the hypoglycemic effect, the weight loss effect of semaglutide has also attracted much attention. By slowing down gastric emptying, increasing satiety and acting on the hypothalamus to suppress appetite, semaglutide is able to effectively reduce dietary intake, thus achieving weight loss. Studies have shown that semaglutide can reduce body weight by 15 to 20%, which is far more effective than previously approved weight loss medications. For obese type 2 diabetic patients, semaglutide can not only help them control blood glucose, but also effectively reduce weight, which is of great significance to improve the metabolic status of diabetic patients.

Efpeglenatide, currently in development, is a long-acting GLP-1RA used to control blood glucose levels in patients with T2DM. Efpeglenatide consists of a modified exendin molecule and is coupled to a fragment of human immunoglobulin 4 by a special technology called long-acting peptide. This special coupling technology allows for a more flexible dosing frequency of efpeglenatide.^265^ Epernatide is also less desensitizing than other therapeutic agents, which means that epernatide has a longer-lasting therapeutic effect. However, eperinatide is still in the developmental stage, and its other related properties and characterization need to be developed in subsequent studies.

**Fig. 8:**
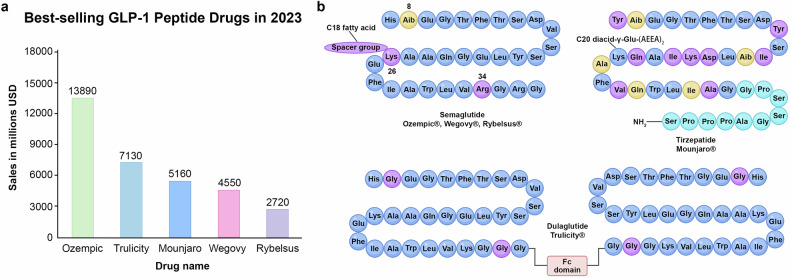
Sales and structures of the top five selling GLP-1 agonists. **a** The total income from sales of the top five selling GLP-1 agonists. **b** The protein composition, linkage order and characteristic structure of the top five selling GLP-1 agonists

Similar to GLP-1, GIP is an intestinally secreted peptide that promotes insulin biosynthesis and islet β-cell differentiation and reduces apoptosis. Clinical trials have shown that the GIP/GLP-1 dual-targeting combination has shown effective weight loss and glucose-lowering effects. Based on these two important targets, Lilly developed tirzepatide (Zepbound^®^), the first marketed dual-target agonist (GIP/GLP-1) obesity treatment injection, which provides better weight control than semaglutide and dulaglutide (Fig. 8b).^266^ The drug has previously been approved as an adjunctive therapy to diet and exercise to improve glycemic control in T2DM.^267,268^ Tirzepatide consists of 39 amino acids, 37 of which are naturally occurring (or coded) and two are non-naturally occurring. Non-coding amino isobutyric acid residues at positions 2 and 13. Besides, it is amidated at the C-terminus, which binds the C_20_ lipoic acid portion through a spacer region attached to Lys20.^269^ This side-chain structure has been cleverly designed to not only improve the stability and bioavailability of the molecule, but also to enhance its affinity for the target. For dual activity, it incorporates amino acid residues primarily from GLP-1 and GIP and uses some unique amino-acid residues. Studies have shown that tirzepatide improves pancreatic β-cell function and insulin sensitivity, and compared to traditional GLP-1 RA, these drugs have a longer duration of action, allowing for a reduction in the frequency of weekly dosing.^270^

Meanwhile, the research and development of triple-target agonists is also a current hot issue. It has been found that retatrutide, a 39 amino acid peptide, can resist cleavage by dipeptidyl peptidase IV, which is responsible for breaking down GLP-1 and GIP,^271^ and it also stimulates GLP-1, GIP and glucagon receptors, which is potentially useful for treating obesity and T2DM. However, studies have shown that retatrutide can increase heart rate by 6.7 beats per minute,^272^ which may be harmful and counteract some of the benefits of weight loss. Retatrutide is currently in the developmental testing phase and more detailed data is pending subsequent studies, but the availability of retatrutide still offers a new direction in the treatment of T2DM.

In addition, there are some potential drugs that may also have an effective effect on diabetes. C-peptide, as a bioactive peptide, can reflect the indicators of pancreatic β-cell function as well as bind to signaling molecules on the surface of the cell membrane and activate its own signaling pathway, exerting antioxidant, anti-apoptotic, and anti-inflammatory effects, or regulating cellular transcription through internalization. It has been found that the bioactivity of C-peptide can be used to prevent and treat the complications of diabetes and thus influence the comprehensive treatment of T2DM. Currently, the relationship between C-peptide and chronic complications of diabetes is complex and clinical trials have been unsatisfactory, requiring control of baseline C-peptide levels. Efpeglenatide, currently in development, is a long-acting GLP-1 RA for controlling blood glucose levels in patients with T2DM.^273^ Efpeglenatide consists of a modified exendin molecule and is coupled to a fragment of human immunoglobulin 4 by a special technology called long-acting peptide This special coupling technology allows for a more flexible dosing frequency of efpeglenatide. At the same time, eptifibatide is less desensitizing than other therapeutic agents, which means that eptifibatide has a longer-lasting therapeutic effect.

#### Progress of GLP-1 receptor agonist repositioning studies

**Fig. 9:**
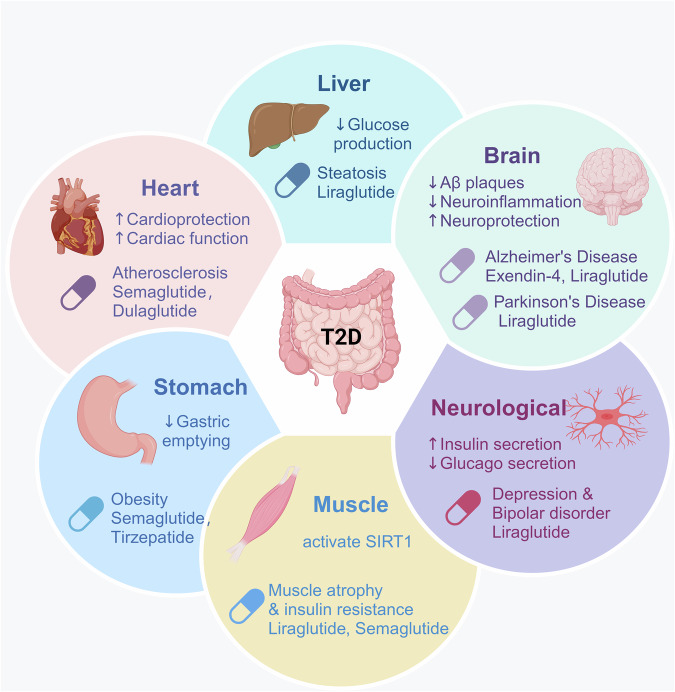
Prospect for peptide drugs targeting GLP-1 agonists in six other tissues and organs. Figure 9 was created with biorender.com

In recent years, GLP-1 has made many important advances in a number of research areas, including the cardiovascular system, central nervous system, obesity and metabolic syndrome, insulin secretion, muscle and liver (Fig. 9). Studies have found that GLP-1 RAs have multiple beneficial effects in the cardiovascular system, including improving cardiovascular function and inhibiting the development and rupture of atherosclerotic plaques, and thus GLP-1 RAs may have cardiovascular protective potential, slightly reducing the risk of death due to cardiovascular disease and any cause, and slightly reducing the risk of stroke compared to placebo. Recent studies have shown^274^ that GLP-1 may also regulate appetite and energy balance by affecting neuronal activity and synaptic transmission, which is critical for appetite and weight regulation. In addition, GLP-1 plays an important role in the pathogenesis of obesity and metabolic syndrome. It has been found that obese patients often have GLP-1 resistance, resulting in reduced GLP-1 bioactivity. Therefore, researchers are exploring ways to increase GLP-1 activity to improve the treatment of obesity and metabolic syndrome. Some scholars’ research shows that Liraglutide can reduce the occurrence of neuroinflammation to a certain extent while reducing Aβ plaques, and the above two are important causes of Alzheimer’s disease (AD), so GLP-1 RAs have great potential in the treatment of AD.^275^ Because Liraglutide has certain anti-inflammatory effect, can reduce the occurrence of neuroinflammation, some scholars believe that it will also play an irreplaceable role in the treatment of Parkinson’s disease. Some experimental studies have shown that Liraglutide can also activate SIRT1 to a certain extent, which is an important factor in regulating muscle cell metabolism, and can prevent muscle atrophy, so GLP-1 RAs provides new ideas for the treatment of muscular dystrophy and other diseases. In addition, Liraglutide has been verified to reduce the possibility of hepatic steatosis by participating in autophagy-lysosomes. The use of semaglutide prior to total hip arthroplasty has been shown to reduce postoperative prosthetic joint infections and readmissions. All of the above suggests that there is great potential for GLP-1 RAs in the treatment of a wide range of conditions.

### Advance of peptide-based therapeutics in cancer

Cancer is one of the primary factors affecting human health and leading to mortality. According to World Health Organization statistics, nearly 10 million deaths (almost one in six) are caused by cancer in 2020.^276^ Despite the breakthroughs in immunotherapy in recent years, the current clinical treatment of tumors is still dominated by chemotherapy, surgery and radiotherap. In this regard, surgical procedures are prone to trauma and bleeding, as well as posing risks of infection and weakened immunity.^277^ Radiotherapy is prone to a variety of complications, as well as high costs and long treatment times.^278^ Chemotherapy has significant side effects and fails to differentiate between normal and tumor cell. In addition, chemotherapy often leads to the development of drug resistance and is prone to relapse^279^; Although immunotherapy has significantly fewer side effects than chemotherapy,^280,281^ the effectiveness of treatment varies with each individual, and it also has the potential to trigger autoimmune myocarditis^282^ or induce cytokine storms.^283^ Therefore, new treatments or anti-tumor drugs are urgently needed to be researched and developed to meet the various needs. Anti-cancer peptides (ACPs) have gradually received attention from researchers in the field of tumor therapy because of their high specificity and safety advantages.

**Fig. 10:**
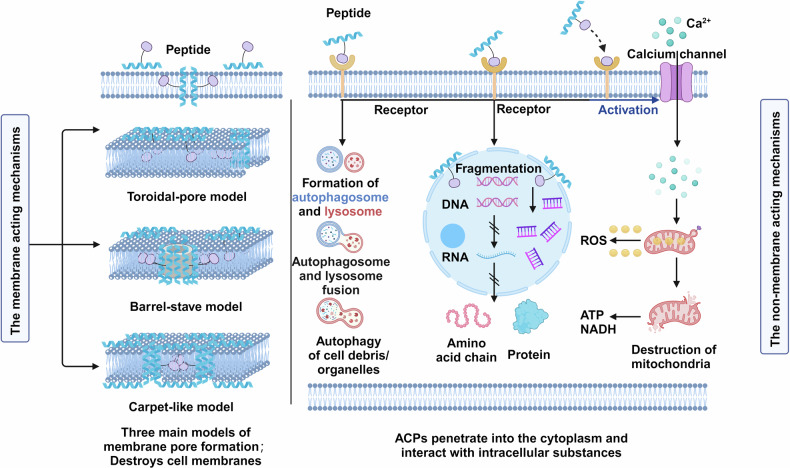
Models of anti-tumor mechanisms of ACPs. The direct mechanism of ACPs is performed through interacting with negatively charged membranes, resulting in increased membrane permeability, cell membrane lysis, or release of intracellular contents, which ultimately leads to cell death. There are three main models of membrane pore formation, namely barrel stave model, toroidal pore model, carpet model. After the antitumor-peptides enter the phospholipid membrane, its hydrophobic region binds to the inner hydrophobic region of the phospholipid bilayer, and the hydrophilic region is exposed to the outside. Another mechanism is that the ACPs penetrates into the cytoplasm and interacts with intracellular substances, such as inhibiting DNA, RNA and protein synthesis, causing autophagy, promoting calcium ion in-flow, causing cleavage of cellular organelles, and disrupting cellular structure by the outflow of contents

ACPs are a class of anti-tumor active peptides within antimicrobial peptides (AMPs), typically possessing a positive charge, high hydrophobicity, and strong penetrability.^284^ Then, it can be used as a hormone and inhibitor or as a peptide vaccine to activate anti-tumor immune responses.^285,286^ Besides, these peptides can be used for imaging, cancer diagnosis, and targeted drug delivery, even some specific peptides can be engineered to attack cancer cells and prevent tumors from worsening.^287^ Based on the types, quantities, and structures of amino acids, ACPs can be classified into four categories: α-helical, β-pleated sheets, random coil and loop structures.^288^ From the perspective of source, it mainly includes natural peptides, peptides obtained from combinatorial libraries (more details in section “Display library technology”), as well as synthetic or modified peptides.^284^ It has complex mechanisms of action, including inhibiting tumor angiogenesis, disrupting cell membranes, interfering with metabolism, targeting cytoplasmic components and mediating immune cell regulation, etc. An overview of the research on peptides in the direction of anti-tumor is presented in Fig. 10.

#### Peptides for anti-cancer

##### A) Natural anti-cancer peptides

Natural ACPs are widely found in animals, plants and microorganisms. For example, oncolytic peptides (e.g., anoplin) can selectively cleave tumor cell membranes and remain effective against drug-resistant tumors.^289^ Some studies have also proved that oncolytic peptides have potential role in activating anti-tumor immunity. The first drug named “oncolytic peptide”, LTX-315 (Ruxotemitide, Oncopore), is currently in phase II clinical trials and can induce complete ablation of a variety of tumors.^290,291^ Wang KR et al. isolated a cationic ACPs, Polybia-MPI, which was originally isolated from the venom of the social wasp Polybia paulista. Its primary sequence is IDWKKLLDAAKQIL-NH2.^292,293^ It targets non-polar lipid cell membranes, forming ion-permeable channels and leading to depolarization, irreversible cytolysis, and eventual cell death. The results showed that polybia-MPI exhibit antitumor activity by disrupting the cell membrane of cells, while it has lower cytotoxicity to erythrocyte and normal fibroblast.^292^ It is also unaffected by conventional multi-drug resistant mechanisms and has the potential to be used as a chemotherapeutic agent against multi-drug resistant tumors.^294^ In addition, anti-tumor peptides, such as Apicidin(cyclo(N-O-methyl-L-tryptophanyl-isoleucinyl-D-pipecolinyl-L-2-amino-8-oxodecanoyl)), can be obtained from fungal metabolites. It is a selective inhibitor of histone deacetylase (HDAC) and has been shown to have potent anti-angiogenic activity and reduce the level of hypoxia inducible factor-1α (HIF-1α) in human and mouse tumor cell lines.^295,296^ In the HCT-116 xenograft tumor model of human colon cancer, Apicidin inhibited tumor growth. Moreover, apicidin also showed anti-tumor activity in Ishikawa cell xenograft tumor model, which could inhibit the proliferation of eight tumor cell lines, including HeLa, MCF-7 and HBL-100.^297^

##### B) Synthesized or modified anti-cancer peptides

Natural peptides as potential drug candidates have the advantages of high selectivity, biocompatibility, diversity of targets of action, and low toxicity, however, natural peptides also have some disadvantages. The instability of some natural peptides can lead to their susceptibility to degradation by gastric acids and enzymes, which reduces their effectiveness in oral drug delivery. In addition, peptides derived from natural sources usually have high production costs, limiting their large-scale application. In order to overcome the limitations of natural peptides, the development of peptide synthesis and modification techniques has become crucial. Synthetic technologies can produce peptides with specific sequences and structures, improving their stability and biological activity. Additionally, modification techniques can change the physical and chemical properties of peptides and enhance their drug compatibility, bioavailability and targetability.^298^ The development of peptide synthesis and modification technologies can further expand the application of peptides as drugs, improve their therapeutic efficacy and reduce their side effects. Here we described typical and some new synthetic peptides. For more details on peptide modification techniques are described in section “Enhancing peptide stability and bioavailability through structural modifications”, where we have summarized them in more detail.

In the 1950s, since du Vigneaud et al.^11^ synthesized the first peptide, Pitocin® (oxytocin), the technology of peptide synthesis has developed rapidly. Peptide synthesis techniques include two categories: biosynthesis and chemical synthesis. Furthermore, Biosynthesis methods can be divided into natural extraction, enzymatic, fermentation and genetic recombination methods, etc. The chemical synthesis of peptides is further divided into SPPS^299^ and Liquid Phase Peptide Synthesis (LPPS).^300^ Both of them play important roles in the field of peptide synthesis.

As far as biosynthesis is concerned, the desired peptides are mainly obtained by microbial fermentation, enzyme catalysis and genetic recombination, etc. For example, the synthesis of formadicin, ramoplanin, vancomycin, and teicoplanin.^301^ Besides, the synthesis of the bis-intercalators family is highly dependent on biosynthetic methods. Bis-intercalators family are a class of *C*_*2*_-symmetric cyclic non-ribosomal peptides produced by actinomycetes, which can be inserted into DNA molecules through two unique chromophores in their structures, hence possessing good biological activities such as antimicrobial and antitumor activities. The complex molecular structure of the double-embedded family of non-ribosomal peptides makes chemical synthesis very challenging, while microbial fermentation is the main method for the production of this family of compounds. Thiocoraline,^302^ Triostin A,^303^ and Echinomycin^304^ are members of the bis-intercalators family, their synthesis is often via biosynthetic methods. Their key core skeletons, such as 3-hydroxyquinaldic acid, (3HQA), quinoxaline-2-carboxylic acid, (QXC) and 6-methoxy-3-hydroxyquinaldic acid, are synthesized in the presence of a range of enzymes (e.g. thioesterase) are synthesized. Finally, a series of fragments are assembled stepwise to obtain the corresponding antitumor peptides.^305^

On the other hand, with respect to chemical synthesis methods. LPPS is mainly composed of two strategies: stepwise synthesis and fragment combination. The advantages of LPPS include low cost, a wide choice of protecting groups, easy to scale up the synthesis, suitable for the synthesis of short peptides, and convenient purification.^300^ SPPS was proposed by Merrifield^299^ in 1963 and has been greatly developed to date. According to the different α-amino protecting groups, it can be divided into tert-butoxy-carbony (Boc) method and 9-fluorenyl-methoxycarbonyl (Fmoc) method.^38,306^ Compared with liquid-phase synthesis, solid-phase synthesis is convenient to operate, easy to realize automated processing, higher product yield and purity, which greatly promotes the development of peptide drugs.^307^ Nowadays, the synergies between solid-phase and liquid-phase synthesis methods play an important role in the field of peptide synthesis. In addition, as auxiliary technologies, microwave-assisted synthesis as well as advances in microchannel flow technology have made it possible to precisely control the reaction time (<1 s) and temperature, which greatly enhances the efficiency.^38,308,309^ For example, the synthesis of feglymycin using a linear method of microfluidic amide bond formation has enabled the preparation of biologically active oligopeptides with highly racemized amino acids, which are attractive drug candidates.^310^

Reniochalistatin E, the only tryptophan-containing cyclo-octapeptide in the reniochalistatins family. To date, it is the only peptide with antitumor activity (including RPMI-8226, MGC-803, HL-60, HepG2, and HeLa) among all the members of the reniochalistatins family, and it is particularly cytotoxic to myeloma RPMI-8226 and gastric MGC-803.^311,312^ It uses 2-chlorotriphenyl chloride (CTC) resin as the carrier and HCTU/DIPEA as the condensation system to synthesize the straight-chain peptide by Fmoc solid-phase synthesis and liquid-phase cyclization to obtain the cyclic peptide crude product. Then it was purified through preparative liquid chromatograph. Next, the product was characterized by HPLC, MS and NMR. Finally, the purity of the target product as well as the overall yield was significantly higher than that of the peptide obtained from this *reniochalina* stalagmitis.^313^ This method is simple and easy to operate, applicable to the synthesis of the series of cyclic peptides.

HYD-PEP06 is a peptide compound obtained via Solid-Phase synthesis by Yang et al.^314^ It is an RGD-modified endostatin-derived synthetic peptide consisting of 30 amino acids. Its N-terminal RGDRGD fragment can specifically bind to different integrins in endothelial cells, which has been shown to have anti-tumor effects in colorectal cancer, oral squamous cell carcinoma and hepatocellular carcinoma (HCC).^315,316^ Based on the “one drug and two targets” design, Yang et al. selected the anti-tumor active fragment of endostatin and carried out structural modification and optimization on this basis, so that it could not only inhibit vascular activity, but also further have de-integrin effect. The mechanism of HYD-PEP06 is to block PI3K/AKT to inhibit the epithelial-mesenchymal transformation of liver cancer cells. Moreover, it also can inhibit the glomus formation and migration of liver cancer tumor stem cells by blocking the Wnt/β-catenin signaling pathway, so as to delay the development of liver cancer. Research has shown that HYD-PEP06 can inhibit the growth of subcutaneous graft tumor remnants of hepatocellular carcinoma in nude mice and has significant therapeutic effects on lung metastasis of hepatocellular carcinoma.^314^ It is a promising drug for the treatment of hepatocellular carcinoma and colorectal cancer by regulating the action of tiny nucleotides, ion channels and tumor stem cells, thus comprehensively inhibiting tumor recurrence and metastasis. Currently, the drug has completed undergoing clinical phase I trials (CTR20213196), and is actively undergoing clinical phase II trials (CTR20220769).

Furthermore, there are a number of globally approved drugs that utilize chemical synthesis techniques, including Sandostatin^®^ (octreotide), Velcade^®^ (bortezomib), Aphexda^®^ (motixafortide).

Octreotide (Sandostatin^®^) is a typical octapeptide derivative synthesized using the Fmoc-SPPS method. The synthesis of octreotide acetate by solid-phase peptide synthesis and the adoption of the trans-salt process has the characteristics of simple operation, easy to operate, higher yield, etc., which are suitable for industrialized production.^317^ It is pharmacologically mimicking natural growth hormone. However, it is more potent than the natural hormone.^318^ Octreotide acts on the somatostatin receptors and causes vascular smooth muscle contraction by inhibiting the coupling of G proteins to phospholipase C. Like somatostatin, it induces an increase in calcium entry through L-type calcium channels, which leads to an increase in calcium-induced calcium release from the sarcoplasmic reticulum in smooth muscle cells through calcium-induced calcium release channels of the ryanodine receptor, then, it initiates the contractile cycle through activation of myosin light-chain kinase via interaction with calcium-calmodulin.^319^ Besides, octreotide can directly inhibit tumor angiogenesis by down-regulating growth hormone (GH) release.^318,320^ And its two formats, injection and slow-release microspheres, were approved by the FDA for the treatment of carcinoid tumors, vasoactive intestinal peptide tumors, in 1988 and 1998, respectively.^321^ Additionally, octreotide is a classic therapeutic peptide that is also used in chemotherapy-associated refractory or intractable diarrhea, graft-versus-host disease, and HIV-associated diarrhea due to cryptosporidiosis, but is not approved by the FDA.^322^ Similarly, there are drugs such as lanreotide and pasireotide that exert the same effect. Furthermore, octreotide has been successfully used in imaging (neuroendocrine, endocrine, breast, small cell lung and prostate cancers) and more recently in targeted radiation therapy.^323^ It is one of the more prominent examples.

Bortezomib (Velcade^®^), a reversible inhibitor of the 26S proteasome, was obtained by chemical synthesis. It can be synthesized by convergent approach, and the use of TBTU (a condensation reagent, O-Benzotriazol-1-yl-N,N,N’,N’-tetramethyluronium tetrafluoroborate) inhibits the racemization during fragment condensation. It was approved by the FDA as a borate peptide for the treatment of multiple myeloma in 2003. Then Approved for mantle cell lymphoma in 2006. This drug exerts its antitumor effects mainly by inhibiting key substances in nuclear factor κB (NF-κB) pathway involved in cell proliferation, apoptosis, and angiogenesis.^324^ However, it is associated with hematotoxicity and peripheral neuropathy, as well as exhibiting poor permeability and pharmacokinetic parameters in solid tumors. Additionally, its chemical stability and bioavailability are low. Recent studies have demonstrated that nanoparticle delivery can overcome these limitations, offering a solution to circumvent the challenges encountered by conventional cancer chemotherapy drug administration.^325^

Motixafortide (Aphexda^®^) is a synthetic cyclic peptide approved by the FDA in September 2023.^326^ It is utilized in combination with filgrastim (granulocyte colony-stimulating factor G-CSF) to facilitate the mobilization of hematopoietic stem cells into the peripheral blood for subsequent collection and autologous transplantation in patients diagnosed with multiple myeloma. Notably, motixafortide is the first innovative drug in nearly a decade to be approved by the FDA in the field of stem cell mobilization in multiple myeloma. It was shown that motixafortide blocks the interaction between CXCL12 and CXCR4 by binding to CXCR4 on hematopoietic stem cells (HSCs) and having a long receptor occupancy (>48 h).^206,327^ Peripheral blood and stored HSCs collected by apheresis were injected back into the patient (autologous transplantation) or into the recipient patient (allogeneic transplantation) to repopulate the bone marrow. Furthermore, motixafortide has been demonstrated to effect “cold” tumors in multiple modes of action, such as immune cell migration, tumor infiltration by immune effector T cells, reduction of immunosuppressive cells (e.g., Myeloid-derived suppressor cells, MDSCs) in the tumor microenvironment, etc. which can turn “cold” tumors (e.g., pancreatic cancer) “hot” (i.e., sensitizing them to immune checkpoint inhibitors and chemotherapy).^206,328,329^ And then, in a Phase II clinical trial, motixafortide in combination with cemiplimab and gemcitabine showed very significant results in the treatment of patients with pancreatic cancer.^206,326^

In the case of chemically modified peptides, for example, Polyethylene glycol (PEG) modification, Amino acid substitution modification and Cyclization modification and, etc. More details on the content of modified peptides are in section “Enhancing peptide stability and bioavailability through structural modifications”. Here we won’t describe it too much.

The occurrence and development of human tumors are related to the inhibition and intracellular degradation of p53 by negative regulatory proteins murine double minute 2 (MDM2) and murine double minute 4 (MDM4 or MDMX) of tumor suppressor p53. Therefore, antagonizing MDM2 and MDMX to activate and stabilize p53 is an important strategy for anti-tumor drug design.^330^ The peptide PMI (TSFAEYWNLLSP), which inhibits p53-MDM2 interaction with high solubility and specificity, was synthesized by phage display technology.^331,332^ However, the side chains of multiple residues of PMI are prone to interact with each other. Based on this, Lu et.al. screened and designed a peptide PMI-M3 (LTFLEYWAQLMQ) with low affinity for MDM2 and MDMX in pmol/L level through systematic mutation analysis and free energy addition principle.^175^ In addition, the researchers also obtained modified peptides of PMI-2K (KTSFAEYWNLLSPK) and M3-2K (KLTFLEYWAQLMQK) by adding lysine residues at both ends of PMI and PMI-M3 to improve their cellular uptake. Moreover, M3-2K can significantly improve the anti-tumor activity of p53-dependent in vitro and in vivo, and this p53-MDM2/MDMX interaction is expected to be further developed as a peptide inhibitor.

#### Peptides used in tumor diagnosis

Peptides can not only be used as drugs, but also used as molecular probe tools for molecular diagnosis and imaging of tumors. Peptides scintigraphy and peptide receptor radiotherapy have been developed based on the fact that tumor cells express one of the five growth inhibitory receptor subtypes. Currently, radioactive elements such as indium 111 (In), yttrium 90 (Y), gallium 68 (Ga), and technetium 99 m (Tc) can selectively bind to different somatostatin analogs via chelating groups.^333^ Here we focus on peptides developed in recent years for use in imaging and diagnostics.

In 2016, [^68^Ga]Ga-DOTA-TOC was approved by the EMA for the specific imaging of tumor cells expressing somatostatin receptors (SSTRs), a radiopharmaceutical that combines the radionuclide ^68^Ga with the somatostatin analog DOTA-TOC to function. Then, the FDA approved [^68^Ga]Ga-DOTA-TOC in 2019 as the first ^68^Ga radiopharmaceutical in the U.S. to use positron emission tomography (PET) to image the somatostatin receptor (SSTR)-positive gastroenteropancreatic neuroendocrine tumors. This radioactive probe will help locate tumors in adults and children with a rare disease, somatostatin receptor-positive neu-endocrine tumors (NETs).^334^

Similarly, Illuccix^*®*^, also called gallium (^68^Ga) gozetotide or gallium (^68^Ga) PSMA-11 was approved by the FDA in 2020 for PET imaging of prostate-specific membrane antigen (PSMA)-positive prostate cancer, in which this radiopharmaceutical combines the radionuclide ^68^Ga with a mimetic peptide, Glu-NH-CO-NH-Lys(Ahx)-HBED-CC, which allows for the PSMA-expressing tumor cells for specific imaging. This targeting approach can also be used to develop treatment plans and potentially to assess treatment response. Notably, it is the first drug to use radioactive ^68^Ga for PET imaging of PSMA-positive prostate cancer.^335^

In addition, Pluvicto^®^ (lutetium Lu 177 vipivotide tetraxetan, ^177^Lu-PSMA-617) is also available for the treatment of adult patients with prostate-specific membrane antigen (PSMA)-positive metastatic castration-resistant prostate cancer (mCRPC), who have received prior androgen receptor (AR) inhibitor and paclitaxel-based chemotherapy, which was approved by the FDA in March 2022.^92^ Similarly, Posluma^®^ (flotufolastat F-18), a high-affinity PSMA-targeted radio-diagnostic reagent based on a novel radio-hybridization technology, was approved by the FDA in May 2023 for testing in males with suspected metastatic prostate cancer for PET (Positron Emission Tomography) of PSMA prostate-specific membrane antigen (PSMA)-positive lesions.^336^

Generally, possessing the advantage of peptides in nature, anti-tumor peptides can inhibit the proliferation, migration and invasion of tumor cells and promote the apoptosis of tumor cells by preventing protein interaction, regulating the conformation of biomolecules, competing for receptor binding and destroying the cell membrane. In addition, anti-tumor peptides are able to be used as molecular probe tools for tumor molecular diagnosis and imaging in the field of tumor drug therapy. At the same time, peptides from different sources can be modified to make them have longer half-life, stronger resistance to enzymatic hydrolysis, relatively complete pharmacokinetic properties. With the in-depth understanding of tumor pathology and the development of new drug discovery technology, it is believed that more anti-tumor peptide drugs will be developed in the future.

### Advance of peptide-based therapeutics in cardiovascular disease

Cardiovascular disease (CVD) is the leading cause of death in the world, accounting for about 50% of all deaths.^337^ The World Heart Report, launched at the 2023 World Heart Summit, shows that CVD deaths jumped globally from 12.1 million in 1990 to 20.5 million in 2021, with four in five of those deaths occurring in low and middle-income countries.^338^ Diabetes mellitus, obesity, hypertension, hyperlipidemia, and other factors are high risk factors for cardiovascular disease, which manifests itself clinically as impaired cardiac function due to myocardial hypertrophy and fibrosis, and ultimately, death resulting from heart failure. A variety of ways have been used in preclinical studies for the treatment of cardiovascular disease, including small molecule drugs, protein drugs, and gene therapy to address the pathologic process.^339–343^ Peptides, due to their advantages, have become important therapeutic agents for cardiovascular diseases focusing on the symptoms of hypertension, vascular function and coronary artery disease, and acute coronary syndromes (ACS). Here, we briefly presented the overview of peptides in cardiovascular diseases.

#### Peptide drugs acting on G protein-coupled receptors

GLP-1 is an incretin secreting hormone secreted by intestinal L cells. GLP-1 receptor is a class B G protein-coupled receptor, which plays an important role in glucose homeostasis and the treatment of T2DM.^344^ GLP-1 exerts its effects through GLP-1 receptor binding. Studies have found that GLP-1 receptor is not only expressed in islet β cells, but also widely distributed in brain, lung, gastrointestinal tract, kidney, liver, heart and other organs in the body.^252,345^ Therefore, GLP-1 RAs may have effects on multiple organs, and the development of such drugs opens new ideas for the treatment of various clinical diseases. The first GLP-1 RAs is exenatide, which was approved by the FDA for the treatment of T2DM in 2005. Studies have shown that GLP-1 RAs is not only effective in glycemic control, but also beneficial in the prevention of cardiovascular disease and weight loss similar to sodium-glucose transporter 2 (SGLT2) inhibitors.^346^ For example, liraglutide, dulaglutide and semaglutide can significantly reduce the incidence of major cardiovascular events (MACE) in patients with T2DM. In addition, the study showed that the risk of MACE in T2DM patients with a history of cardiovascular disease was significantly reduced by 14%, while the risk of MACE in patients without a history of cardiovascular disease was significantly reduced by 6%.^345,347^ In addition, the use of GLP-1 RAs in nondiabetic patients has focused on a modest improvement in left ventricular function after 7 days of acute treatment with the GLP-1 RA in patients with ST-segment elevation myocardial infarction (STEMI).^348,349^ Similar results were seen in patients without STEMI, independent of diabetes status. This suggests that GLP-1 RAs has some preventive effect on cardiovascular events in nondiabetic populations, but more study data are needed to support this. Although the peptide GLP-1 RAs is not FDA-approved for the treatment of cardiovascular disease, the potential therapeutic pleiotropic effects of the peptide GLP-1 RAs in patients with cardiovascular disease may extend beyond the treatment of diabetes in the future.^350^

Human urotensin-II (UII) is the strongest vasoconstrictor found in mammals.^351^ and it acts through the activation of the UII receptor (UT), the orphan G protein-coupled receptor (GPR14), collectively referred to as the UII/UT system.^352^ Urantide is a peptide UT antagonist derived from Urotensin II(UII).^353^ Urantide contains a core peptide consisting of six amino acid residues (Cys-Phe-Trp-Lys-Tyr-Cys), which has certain biological activity. Therefore, urantide has a high affinity with the UTs of human, mouse, monkey and other animals. Studies have shown that urantide can reduce the content of oxygen free radicals and anti-lipid peroxidation in myocardial tissue by activating PI3K /Akt and PKC signal transduction pathways, further regulate the expression of Bcl-2-associated X (Bax) and B-cell lymphoma 2 (Bcl-2) proteins, and inhibit the apoptosis of myocardial cells in rats with myocardial ischemia/reperfusion.^198^ Attenuating cardiotoxicity induced by doxorubicin and protecting primary cardiomyocytes depended on the down-regulation of p38 in the MAPK pathway.^354^ The protective effect of urantide on DOX-induced myocardial injury was more obvious. In studies where allantoin was administered to rats with atherosclerosis, it was found that allantoin reduced myocardial injury and lowered, serum creatine kinase (CK) and lactate dehydrogenase (LDH) levels by blocking the UII/UT system and regulating the mitogen-activated protein kinase (MAPK) signaling pathway. Urantide also reduced the levels of UII and its receptor, p38, p-extracellular signal-regulated kinase (ERK) and p-c-Jun N-terminal kinase (JNK) in myocardial tissue to protect cardiovascular function.^354,355^ At present, the research of urantide in cardiovascular diseases is still in the stage of animal experiments.

#### Peptide drugs acting on natriuretic peptide receptors

Natriuretic peptides (NPs) family consists of atrial natriuretic peptide (ANP), brain natriuretic peptide (BNP), and C-type natriuretic peptide (CNP).^356^ play an important role in the prevention of plasma volume expansion and hypertension.^357^ Natriuretic peptide regulates cardiovascular homeostasis mainly through three membrane receptors: natriuretic peptide receptor A (NPR-A) and natriuretic peptide receptor B (NPR-B) are guanylyl cyclase-coupled receptors, and natriuretic peptide receptor C (NPR-C) is a non-guanylyl cyclase-receptor on the cell surface.^358^ NPs mainly act through NPR-A and/or NPR-B receptors, while NPR-C is mainly used to remove NPs.^359^ The cardiovascular effects of natriuretic peptides include a reduction in peripheral vascular resistance and cardiac preload.^360,361^ Nesiritide, a recombinant human brain natriuretic peptide (rh-BNP) that mimics brain natriuretic peptide (BNP) action, plays a role in patients with decompensated heart failure. Clinical studies with intravenous injections have shown that, Nesiritide has a potent, dose-related vasodilator effect that is rapid and long-lasting. It was approved by the FDA in 2001 for the treatment of acute decompensated heart failure in patients at rest or with mild dyspnea.^362,363^ However, nesiritide is not widely used due to, which has side effects of headache and decreased blood pressure, low specificity and safety, now withdrawn from the market.^362,364^

Carperitide, a cyclic, recombinant α-human atrial natriuretic peptide (hANP), was approved by the PMDA in 1995 for the treatment of acute congestive heart failure (ADHF).^365,366^ ANP often induces a biological response by binding to guanylate cyclase-coupled receptor NPR-A. The use of carperitide in patients with ADHF can reduce central filling pressure and plasma aldosterone concentration, increase cardiac output, diuresis, and improve hemodynamics in the acute phase of ADHF.^367^ Hypotension was considered to be the most common adverse effect of caperitide, followed by renal dysfunction, and the increased in-hospital mortality in ADHF patients was significantly associated with the use of caperitide. However, a low dose (0.025-0.050 μg/kg/min, 0.0125 μg/kg/min in some cases) of continuous intravenous infusion of carperitide can reduce side effects and in-hospital mortality, but this report still needs to be confirmed by a large number of samples and multi-center trials.^368^ In addition, carperitide can also increase coronary blood flow (CBF), reduce myocardial contraction and metabolic dysfunction, and limit infarct size. Among them, nitric oxide (NO) plays an important role in carperitide-induced ischemic heart vasodilatation and cardioprotection.^369^

#### Peptide drugs acting on angiotensin-converting enzyme (ACE)

Hypertension is a common chronic cardiovascular disease characterized by persistently elevated arterial blood pressure. It is a major risk factor for cardiovascular diseases such as atherosclerosis, coronary heart disease, stroke and myocardial infarction, besides, it is also a major cause of premature death worldwide. Angiotensin-converting enzyme inhibitors, angiotensin II receptor blockers, calcium channel blockers, β-adrenoceptor blockers and diuretics are commonly used antihypertensive drugs in the world.^370^ These antihypertensive drugs have good antihypertensive effects, but there are also many side effects. For example, some drugs that normally lower blood pressure may abnormally raise blood pressure, or increase blood pressure after stopping the drug, and cause symptoms such as dry cough, rash, edema, and acute renal impairment.^371,372^ Among the active peptides that have antihypertensive effects, most of them achieve their antihypertensive effects by inhibiting the activity of ACE. Active peptides with antihypertensive effects are similar to other peptide drugs in that they exert their therapeutic effects (lowering blood pressure) without any side effects on the body. Ace-inhibiting peptides are derived from a wide range of animal, plant and Marine organisms through the hydrolysis of hydrolases (e.g., pepsin, chymotrypsin and trypsin) and microbial enzymes (e.g., alkaline protease, thermolysin, conditase and proteinase K).^373^

LKPNM, a peptide derived from Katsuobushi, is an ACE inhibitor with antihypertensive activity, it has been used as a pharmaceutical ingredient in antihypertensive capsules.^374,375^ ACE-inhibiting peptides have also been identified from other species. For example, ACE-inhibiting peptides have been identified from other species, such as four peptide sequences (Gly-Gly-Pro-Ala-Gly-Pro-Ala-Val, Gly-Pro-Val-Ala, Pro-Pro, and Gly-Phe) isolated and extracted from salmon gelatinase hydrolysate (SC-1), which possess ACE and dipeptidyl peptidase IV (DPP-IV) inhibitory activity as well as oxygen radical uptake capacity. The peptides showed strong antihypertensive effects in a rat model of spontaneous hypertension, suggesting that the peptides could be used as pharmaceutical ingredients for the treatment of hypertension and related diseases.^376^ The results indicate that this polypeptide may be used as a drug preparation for the treatment of hypertension and related diseases.

Novel ACE-inhibiting peptides, VVLASLK, LTLK, LEPWR, ELPPK and LPTEK, were screened, identified and synthesized from cutlass, among which peptide LEPWR exhibited the best ACE-inhibiting ability. The antihypertensive effect of the ultrafiltration fraction was confirmed by using a spontaneously hypertensive rat (SHR) model. LEPWR antagonize ACE in a mixed competitive mode and forms six hydrogen bonds with ACE. The present study demonstrates that the hypotensive effects produced by the cutlery are attributed to these peptides.^377^ Identification of novel ACE inhibitory peptides from Pacific saury: In vivo antihypertensive effect and transport route. Dual inhibitory properties of mechanically boneless chicken residue (MDCR) hydrolysate on angiotensin-I converting enzyme (ACE-1) and dipeptidyl peptidase 4 (DPP4). Using food-grade protease to hydrolyzate MDCR, a potent peptide with dual inhibitory effects on ACE-1 and DPP4 was identified and then isolated to obtain IY (ACE-1-inhibitor) and VL (DPP4-inhibitor) peptides with dual effects on blood pressure and blood glucose regulation. Low Molecular Weight Peptide Fraction from Poultry Byproduct Hydrolysate Features Dual ACE-1 and DPP4 Inhibition.^378^

#### Peptide drugs acting on GPIIb /IIIa receptors

Eptifibatide, a ring heptapeptide derived from a protein in the venom of crotchtails, can be specifically recognized by GPIIb /IIIa receptors on the surface of platelets, blocking the binding of fibrinogen to GPIIb /IIIa receptors and inhibiting the final pathway of platelet aggregation. Its ring structure increases the bioavailability of the drug and its resistance to plasma proteases.^379,380^ It has been approved by the FDA for clinical use in Acute Coronary syndrome (ACS), including heart attacks and other emergencies such as sudden cessation of blood supply to the heart.^381,382^

Acute coronary syndromes are recognized by damage to the walls of the coronary arteries, resulting in the formation of intraluminal thrombi that block one or more coronary arteries, leading to unstable angina, non-ST-segment elevation myocardial infarction, and ST-segment elevation myocardial infarction.^9^ Antiplatelet therapy may be a therapeutic basis for the prevention and treatment of recurrent cardiovascular events in patients with acute coronary syndrome and undergoing percutaneous coronary intervention (PCI). In addition, it has been shown that intracoronary eptifibatide combined with PCI in the treatment of non-ST-segment elevation-acute coronary syndrome (NSTE-ACS) can effectively improve blood flow, increase myocardial perfusion to a certain extent, reduce perioperative platelet aggregation, which can improve cardiac function, with better results than PCI alone, besides, it has a better safety profile than that of abciximab.^383,384^ Furthermore, compared with the non-peptide small molecule GPIIb /IIIa receptor antagonists orbofiban, xemilofiban, sibrafiban and roxifiban (high side effects, such as thrombogenicity and high mortality),^385^ eptifibatide has little antigenicity.^386^ Eptifibatide has stronger binding ability to GPIIb /IIIa receptors, better safety, and faster action. The results of comparative experiments showed that intravenous administration of eptifibatide avoided the pre-systemic metabolism of liver and gastrointestinal enzymes, resulting in complete systemic availability. At a given dose, the pharmacokinetics of eptifibatide is linearly dose-dependent, and the antiplatelet activity of regular doses of eptifibatide is superior to that of tirofiban (GPIIb/IIIa inhibitor).^379^

#### Peptide drugs acting on tyrosine kinase receptors

Neuroglial protein (NRG)-1, also known as neural differentiation factor (NDF) or glial growth factor (GGF), is expressed in the cardiovascular system, nervous system, gut, kidney, and mammary gland.^387,388^ It is a ligand for tyrosine kinase receptors of ErbB3 and ErbB4 and structurally belongs to the epidermal growth factor (EGF) family. It directly binds to the ErbB4 receptor on cardiomyocytes, which activates the receptor and produces the corresponding bioactivities. The NRG-1/Erb B signaling system is not only involved in the regulation of cardiac embryonic development, but also closely related to the formation of cardiac structure, the maintenance of cardiac function, and the development of heart failure. Recombinant human neuromodulin (rhNRG)-1 peptide can attenuate myocardial injury in various animal models of cardiomyopathy. Additionally, it has therapeutic effects on heart failure. Phase II (CTR20192276) and phase III (NCT05949801) clinical trials of rhNRG-1 in heart failure are in progress.

### Advance of peptide-based therapeutics in infection

Anti-infection is an important area of therapeutic peptides. The infection is caused by pathogens such as bacteria, viruses, fungi, parasites, etc. Infectious diseases are one of the major challenges in current clinical medicine. Penicillin is the first anti-infective drug applied in the clinic in the world. After years of development, more and more antibiotics have sprung up and made significant contributions to the cause of resisting bacterial infections for all mankind, but the problem of drug resistance caused by the misuse of antibiotics has also gradually come to the fore. In 2019, it is estimated that nearly 5 million deaths were related to antimicrobial resistance, of which 1.27 million were directly caused by antimicrobial resistance. At the same time, “superbugs” are constantly expanding outwardly. As a result of drug resistance, antibiotics and other antimicrobial medicines become ineffective and infections become increasingly difficult or impossible to treat.^389,390^ The development of new antibiotics is insufficiently motivated globally, and there is an urgent need for new anti-infective drugs, which makes the research on antimicrobial peptides one of the current hotspots. More than 3000 antimicrobial peptides (AMPs) have been identified to date. Among them, gramicidin, daptomycin, colistin, vancomycin, oritavancin, dalbavancin and telavancin has been approved by the FDA for the treatment of bacterial infections.^391^

Antimicrobial peptides (AMPs), as a class of possible alternatives to antibiotics, are good candidates for overcoming antibiotic resistance due to their high antimicrobial activity, broad antimicrobial spectrum, variety, specificity, and the fact that the target strains are not prone to resistant mutations.^392^ It is a small molecule peptide that plays an important role in the host innate immunity. Most AMPs are short (10-50 amino acids), possess a positive charge (ranging from 2 to 11), and contain a large percentage of hydrophobic residues (usually 50%).^391,393,394^ Structurally, AMPs have α-helical, β-sheet, or extended/random-coil structures.^395–397^ Traditional antibiotics target an enzyme or a protein along a metabolic pathway, however, bacteria can produce new proteins through genetic mutations and thus become resistant to the drug, most antimicrobial peptides act by disrupting the membrane integrity of the target organisms and/or by transmigrating across microbial membranes to reach intracellular targets.^398^ Most antimicrobial peptides can kill microbial pathogens directly and the antimicrobial action of antimicrobial peptides tends to be very rapid, e.g., tick-defensin-derived Os(3-12)NH2 and computer-designed PaDBS1R1 peptides both kill microorganisms within 5-10 minutes of the exposure.^399,400^ Moreover, many of these antimicrobial peptides have a wide spectrum of antimicrobial effects, including activity against gram-positive and gram-negative microorganisms, fungi, unicellular protozoa and viruses. Also, some AMPs exhibit immunomodulatory activity, resulting in the indirect facilitation of pathogen clearance from the host.^400–403^

In terms of AMPs structure, the net positive charge of AMPs is capable of electrostatic interaction with negatively charged microbial membranes, and it has low selectivity to neutrally charged mammalian cell membranes. The hydrophobic residues enable them to penetrate cells and induce membrane cleavage. Meanwhile, the increased hydrophobicity of amino acid sequences can also reduce the selectivity and toxicity to mammalian cells.^404^ In fact, AMPs still contain some anions. However, its mechanism of action is difficult to determine, and it still has a significant role in anti-infection. In addition to their antimicrobial properties, AMPs play a pivotal role in intracellular processes such as angiogenesis, arteriogenesis, inflammatory responses, cell signaling, and wound healing, which makes them interesting candidates for research and development of the innovative drug.^405–407^

#### Antibacterial mechanisms

As mentioned above, most AMPs contain a net positive charge, a considerable proportion of amino acid hydrophobic residues, and contain α-helix, β-sheet and other secondary structures, which play an important role in antibacterial and anti-infection.^404^ It has great prospects in the field of anti-infection. At present, the mechanism of action of AMPs mainly includes two modes of action: Membrane action model and non-membrane action model.^408,409^ In membrane interaction models, disruption of microbial membranes by AMPs can occur through different mechanisms, including disruption of the lipid bilayer (barrel-stave model and toroidal-pore model), thinning of the membrane lipid bilayer, and subsequent membrane solubilization (carpet model, and aggregate model).^410^ On the other hand, some AMPs do not rely on a direct membrane breakdown mechanism but pass through the bacterial cytoplasmic membrane without necessarily disrupting it, then it interferes with fundamental processes such as DNA and protein synthesis, or inhibit other intracellular targets.^411,412^ Of these, binding of AMPs to bacterial cell membranes occurs through electrostatic interactions between cationic AMPs and anionic lipopolysaccharides (lipopolysaccharides from gram-negative bacteria) or (lipoblasts and peptidoglycans from gram-positive bacteria), and the subsequent invasion of AMPs into the cell exerts their respective interactions to achieve anti-infective properties.^413^ Nowadays, a variety of peptide antibiotics and non-antibiotic anti-infective drugs are under pre-clinical feasibility study or marketed for the treatment of infectious diseases.

#### Classical AMPs

Daptomycin, a cyclic lipopeptide antibiotic developed and marketed by Cubist Pharmaceutical Company in 1987, is a classic antimicrobial peptide drug. It has become the world’s first cyclic lipopeptide antibiotic and it is the first-line drug for the treatment of infections caused by drug-resistant Gram-positive bacteria. In 2003, daptomycin was approved for use in the United States for the treatment of complex and structural skin infections caused by gram-positive bacteria, as well as bacteremia and right-sided endocarditis caused by Staphylococcus aureus.^414^ Daptomycin mainly acts on the cell membrane of gram-positive bacteria. Within neutral pH, daptomycin has a negative charge and its antibacterial activity is dependent on calcium ions. When daptomycin is close to the bacterial cell membrane, it oligomerizes under the action of calcium ions and forms an “ion channel” like structure on the cell membrane, which causes intracellular ions to flow out, rapidly depolarizes the cell membrane, and blocks the synthesis of RNA, DNA and macromolecular proteins, and finally, bacterial death due to these factors.^415–417^ In addition, surotomycin, optimized by the fatty acid side chain of daptomycin, has been shown to have a rapid bactericidal effect on Clostridium difficile infection (CDI). It is currently in phase III clinical trials for the treatment of CDI.^418–420^

Dalbavancin (Xydalba^®^), a second-generation, semi-synthetic lipoglycopeptide antibiotic, has strong antibacterial activity against a variety of gram-positive bacteria (including methicillin-resistant Staphylococcus aureus MRSA and Streptococcus pyogenes) and some streptococci, with a long half-life and good tolerance.^421,422^ Xydalba^®^ was approved by the EMA in 2015 for the treatment of acute bacterial skin and skin structure infections (ABSSSI) caused by gram-positive bacteria, including methicillin-resistant Staphylococcus aureus (MRSA). Interestingly, dalbavancin, one of the star drugs in the field of antibiotics, was first discovered and developed by Vicuron company in the United States, and then passed through Pfizer and Durata Company before it was approved by the FDA in May 2014. Based on the positive results of two randomized, double-blind and multi-center clinical trials (SOLO I and SOLO II trials), FDA approved a new antibiotic, which is named Oritavancin in the same year.^423–425^ Oritavancin injection for the treatment of ABSSSIs caused by gram-positive bacteria (including methicillin-resistant Staphylococcus aureus, MRSA) in adult patients. Oritavancin and dalbavancin (approved by the FDA in 2014), as well as the vancomycin (approved by the FDA in 1954), are belong to the new second-generation, semi-synthetic glycopeptide antibiotics. Notably, it is the first and only antibiotic for the treatment of ABSSSIs with a single-dose regimen.^423^

Rezafungin (Rezzayo^®^) was approved by the FDA in March 2023 for the treatment of candidemia and invasive candidiasis in adults, the first therapeutic treatment approved for invasive candida infections in nearly a decade.^426^ Rezafungin is a novel echinocandin that acts by inhibiting β-1,3-glucose synthetase thereby disrupting the integrity of fungal cell wall.^427,428^ The approval of this drug further expands the application of AMPs in the field of anti-infectives. Besides, there are some promising AMPs under study.

The antimicrobial peptide peceleganan (PL-5) spray is a non-antibiotic anti-infective drug. As a new type of peptide broad-spectrum anti-infective drug, it has a unique bactericidal mechanism.^429^ It acts as a bacterial penicillin binding proteins family inhibitor (PBPs inhibitor), showing strong bactericidal advantages against gram-positive, Gram-negative and various antibiotic-resistant bacteria. It can be used for various wound infections such as burns, traumatic ulcers, venous ulcer infections and Wagner class II diabetic foot ulcers, etc. Currently, antimicrobial peptide PL-5 spray is in the U.S. Phase II clinical study (NCT06189638), which is expected to achieve better results.

Teixobactin, a novel peptide antibiotic obtained by screening bacteria from soil samples has a wide range of activity against multi-drug resistant gram-positive pathogens such as methicillin-resistant staphylococcus aureus, streptococcus pneumoniae, and vancomycin-resistant enterococci.^430^ Teixobactin targets the peptidoglycan precursor lipid II, which has the dual effect of inhibiting peptidoglycan synthesis and disrupting cell membranes, enabling an effective attack on bacterial cell membranes.^431–433^ Antimicrobial experimental studies in vitro have shown that teixobactin has a good inhibitory effect on gram-positive bacteria and is non-toxic to mammalian cells, as well as non-hemolytic and non-genotoxic, which make it a promising drug lead-compound.^434^

Recently, Li et al. reported that CT-K3K7, a scorpion antimicrobial peptide derivative, was able to inhibit the growth of Candida both in vitro and in vivo. CT-K3K7 can kill Candida by destroying cell membrane or nucleus and interacting with nucleic acid. It can also induce candida cell necrosis and inhibit its mycelium and biofilm formation through reactive oxygen species (ROS)-related pathways. In the mouse model of subcutaneous infection, CT-K3K7 significantly prevented skin abscess formation and reduced the number of recovered Candida cells in the infected area. Therefore, CT-K3K7 is expected to be a promising drug for the treatment of Candida skin infections. In addition, there are a number of AMPs derived from natural sources, as well as AMPs from engineered sources, that play an important role in the field of anti-infection. Such as the rhesus theta-defensin-1 (RTD-1),^435,436^ a natural macrocyclic AMP expressed in primate leukocytes. Its macrocyclic structure confers drug stability and resistance to cleavage by the large number of proteases found in the saliva of cystic fibrosis (CF) patients, furthermore, it is more effective than the usual natural AMPs. In addition, RTD-1 showed rapid in vitro bactericidal activity against mucoid, non-mucoid and multi-drug resistant clinical isolates of Pseudomonas aeruginosa, without cross-resistance to colistin. Nowadays, it is considered as a potential therapeutic agent for CF airway infections. WLBU2 is a cationic amphipathic peptide with membranolytic activity composed of Arginine, Valine and Tryptophan in different arrangements.^437^ Due to its broad-spectrum antimicrobial activity against ESKAPE (Enterococcus faecium, Staphylococcus aureus, Klebsiella pneumoniae, Acinetobacter baumannii, Pseudomonas aeruginosa and Enterobacter species) pathogens.^438–440^ the molecule is currently in Phase I clinical trials (NCT05137314) for the treatment of periprosthetic joint infections.

Overall, AMPs have been initially used in the field of biomedicine research with their unique advantages. AMPs not only have certain inhibitory effects on bacteria, fungi and anti-parasites, but also a certain inhibitory and killing effects on enveloped viruses and tumor cells. The broad-spectrum antibacterial activity of AMPs has been studied as an alternative to antibiotics. It is believed that AMPs will be more rapidly developed in the field of biomedicine in the near future.

### Advance of peptide-based therapeutics in anti-coronavirus

Currently, the coronaviruses(COVs) that infect humans (HCoVs) contain seven COVs.^441^ Within these, Severe Acute Respiratory Syndrome Coronavirus 2 (SARS-CoV-2), Middle East Respiratory Syndrome Coronavirus (MERS-CoV), and SARS Coronavirus (SARS-CoV) are highly pathogenic COVs that can cause serious illness and even death. In contrast, HCoV-229E, HCoV-OC43, HCoV-NL63 and HCoV-HKU1 are considered low pathogenic HCoVs as they usually cause mild disease in humans.^442^ Novel Coronavirus 2019 (COVID-19), which is caused by SARS-CoV-2 infection, has led to about 770 million diagnosed cases and more than 7 million deaths worldwide as of January 24, 2024 (From https:// covid19.who.int). For the prevention and treatment of COVID-19, there are three dominant antiviral options: vaccines, neutralizing antibodies, and small molecule drugs.^443^ Among them, vaccines can realize early prevention to a certain extent, neutralizing antibodies are expected to be able to directly block the invasion of the virus into the host cell, while small molecule drugs can block the replication of the virus in the host cell by targeting the core shared proteins, which play a therapeutic role in post-infection treatment and disease control.^444^ It is hoped that long-term effective and broad-spectrum small molecule drugs can be developed. With regard to vaccines and neutralizing antibodies, SARS-CoV-2 continues to spread globally with the mutation of the new COVs, and new variants are still emerging rapidly, posing a major challenge to current vaccine and therapeutic approaches. Thus, this calls for a sustained supply of highly effective, low-toxicity antiviral drugs to combat SARS-CoV-2 and its variants. Peptide drugs are gaining attention in the development of anti-SARS drugs due to its low immunogenicity, low cost, specificity and modularity enabling it to be tailored to the virus.

#### Development of anti- COVs candidate targets

In the process of viral invasion, viruses need to enter the target cells through the steps of receptor recognition and membrane fusion or endocytosis, in which the receptor-binding subunit of the viral surface protein (SP) plays a role in mediating the recognition between the virus and the cell receptor, and the transmembrane subunit of the viral SP plays a role in mediating the fusion of the membrane, which are the common antiviral targets in this process. In addition, there are receptors on the cell, host cell proteases needed to cleave the SP, and so on. After the virus enters the target cell, it releases its own DNA/RNA into the cell, which serves as a template to direct the synthesis of viral proteins. At the same time the viral genome undergoes simultaneous self-replication. During this process, certain viral proteases act to cleave viral precursor proteins, while RNA viruses require RNA-dependent RNA or DNA polymerases (RdRp and RdDp) for replication. The RdRp and RdDp are not present in the human body, so viral proteases as well as RdRp and RdDp are also important antiviral targets. Then, the newly synthesized viral genome and viral proteins assemble into new viral particles, which are released to the outside of the cell for dissemination through budding or cell trafficking pathways. Within the same viral genus, the sequences and structures of proteins that perform the same function are often highly similar, such as the spike (S) protein structures of SARS-CoV and SARS-CoV-2. These proteins often serve as common targets for the development of antiviral drugs.^445^

The protein structure of COVs includes structural proteins and Non-structural proteins (NSPs). The structural proteins are composed of the spike (S) protein, envelope (E) protein, membrane (M) protein and nucleocapsid (N) protein.^446^ Theoretically, all the COVs enzymes and proteins involved in viral replication and control of the host cell machinery are potential drug targets, including S protein, papain-like protease (PL^Pro^) and master protease (M^pro^/3 CL^pro^), and viral RNA-dependent RNA polymerase (RdRP). This chapter focuses on the use of peptides in COVID-19, with a brief overview of hot peptide targets in COVID-19.

##### A) The S protein, a key protein in COVs, and its role

The coronavirus spike S protein plays an important role in the invasion of COVs. Similar to other COVs, SARS-CoV-2 infection requires the fusion of the viral envelope and cell membrane, which is mediated by the viral spike (S) glycoprotein.^447^ It regulates viral entry into host cells and is also the major antigenic determinant, often the target of the host antibody response. The COVID-19 is caused by severe acute respiratory SARS-CoV-2 infection. S protein is an important protein that mediates virus entry into cells. The receptor binding domain (RBD) on the S1 subunit is responsible for the binding of the virus to the cell surface receptor.^448–451^ Many neutralizing antibodies target this region to inhibit RBD binding to the viral receptor, thereby blocking COVs infection. However, this region is not conserved and there is great variability among COVs, which makes most of the neutralizing antibodies targeting this region lack broad-spectrum and can only inhibit one or a few COVs.^446^ The heptad repeat 1/2 (HR1/HR2) region of the S2 subunit can interact with each other to form a six-helix structure, which mediates the fusion of the virus with cells and the entry into cells for replication. The formation of the homologous hexahelix can be blocked by the addition of exogenous peptides that can interact with the HR1 or HR2 region of the S2 subunit, thereby inhibiting the invasion of the virus into cells. In addition, the mechanism of membrane fusion between virus and target cells is very conserved in different COVs, and the six-helix bundle formation between the HR1 and HR2 domains plays a key role in driving membrane fusion, which also makes this region an important target for the development of broad-spectrum viral fusion/invasion inhibitors.

In addition, researchers found that the affinity of the S protein of SARS-CoV-2 to the human Angiotensin-converting enzyme 2 (ACE2) receptor was much higher than that of SARS.^448^ Based on the researchers’ elucidation of the electron microscopic structures of the full-length proteins of the ACE2-B0AT1 complex and the ACE2-RBD complex, which revealed how the S protein of 2019-nCoV interacts with the receptor ACE2 at the atomic level, the structural basis and functional characterization of the entry of SARS-CoV-2 into the target cells were further clarified. The above results show that 2019-nCoV enters human cells by targeting the receptor domain (RBD) of the viral transmembrane S protein to the receptor protein, ACE2. In other words, the interaction between S protein and ACE2 is the key way for the virus to enter cells. ACE2 is like the “door handle”, and SARS-CoV-2 opens the door to enter human cells by grasping the “door handle”. Therefore, blocking the interaction between ACE2 and 2019-nCoV S protein can be used as one of the potential effective ways to prevent and control COVID-19.

##### B) 3CL protease (3C-like protease, 3CL^PRO^, or M^pro^)-a key enzyme in COVs replication

COVs are enveloped viruses with a positive-stranded, single-stranded RNA genome of 26 to 32 kb in length. During SARS-CoV-2 infection of the host, the S protein on the outer surface of the viral particles is responsible for binding to the host receptor for attachment to the cell membrane, followed by fusion of the viral and host cell membranes and release of the viral genomic RNA into the cell. Next, there is fusion of the virus and host cell membranes and release of viral genomic RNA into the cell. Subsequently, two viral replicase polyproteins are translated into two polyproteins, pp1a and pp1ab, by controlling the production of two viral replicase polyproteins by host ribosomes.^452^ These can be further processed by viral-encoded proteases in 16 mature nonstructural proteins (NSPs). The main protease (M^pro^), or 3C-like protease (3CL^pro^), is an extremely important protease in the propagation of other coronaviruses, such as Middle East Respiratory Syndrome Coronavirus (MERS-CoV), which is responsible for the cleavage of protein precursors of the translated viral genome, resulting in a number of nonstructural proteins (NSPs), that assemble to form the viral replication-transcription enzyme complex NSPs. Finally, these non-structural proteins are assembled to form the viral replicase-transcriptase complex (RTC).^453^ Therefore, 3CL^pro^ inhibitors can be developed to inhibit the activity of 3CL^pro^ and interfere with the viral replication process. Moreover, since of 3CL^pro^ is highly conserved among different genera of COVs and has no homologous proteins in the human body, it implies that the design of inhibitors by using it as a drug target can achieve a better expectation of selectivity and safety, and it has a great potential to be explored, which has become the direction of research in the field of human in the anti-coronaviral field.

#### Peptides for coronaviruses (COVID-19 as an example)

Paxlovid^®^ is currently the only peptidomimetic drug approved by the FDA for severe symptoms caused by COVID-19. In December 2021, Emergency Use Authorization (EUA) of Paxlovid by the FDA for the treatment of SARS-CoV-2 in mild-to-moderate adults and pediatric and adult patients ≥12 years of age with a body mass of ≥40 kg, and in patient populations at higher risk of severe disease.^454,455^ Preliminary results of a phase III clinical trial showed that patients who were treated within 3 days of symptom onset had an 89% lower risk of hospitalization and death from any cause than those who received placebo.^456^ Until May 2023, the drug was formally approved by the FDA from Emergency Use Authorization (EUA).

In fact, Paxlovid^®^ is a COVID-19 combination product, a combination of the 3CL protease inhibitor PF-07321332 (also known as Nirmatrelvir) and low-dose Ritonavir, both peptide analogues.^455,457^ Development of PF-07321332 began with the SARS-CoV outbreak in 2002. During the SARS-CoV epidemic period, Pfizer’s researchers tried to design SARS-CoV 3CL protease inhibitors based on Rupintrivir (an irreversible inhibitor of human rhinovirus 3CL protease) as the starting point. After a series of optimization, PF-00835231 was obtained. However, the inhibitor has not yet been tested in animals, and it has been shelved due to the demise of the SARS-CoV epidemic. It was restarted in 2020 due to the outbreak of COVID-19. Ritonavir was developed to solve the problem that multiple sites of PF-07321332 are oxidized and metabolized by CYP3A4 (cytochrome P450 3A4 enzyme) in liver microsomes.^457,458^ Ritonavir, as a pharmacokinetic enhancer of Nirmatrelvir, slows down its breakdown in vivo and enhances its half-life and bioavailability by inhibiting the degradation of Nirmatrelvir by CYP3A4. so that it remains active in the body for a longer period of time and at higher concentrations to help fight the virus.^455,459^ This strategy is also borrowed from previous antiviral drug delivery strategies. Efficacy data from the phase II/III study of Paxlovid showed an 86% reduction in the risk of COVID-19-related hospitalization or death from any cause among patients who received Paxlovid^®^ within five days of symptom onset. Oral Paxlovid is superior to intravenous treatments such as Remdesivir (approved by the FDA in October 2022),^460^ as well as lower hospitalization and mortality rates than Molnupiravir (approved by the FDA in 2022), since then, Paxlovid^®^ (PF-07321332 and Ritonavir) was born and remains relevant for the treatment of COVID-19 and some of its variants. This antiviral therapy has also been derived from the oral anti-COVID-19 innovative drug Simnotrelvir/Ritonavir, which is safe and effective, and has shown remarkable efficacy and safety in clinical II/III trials.

EK1 polypeptide, a peptide inhibitor developed by Jiang et al., broadly inhibits multiple human COVs capable of infecting humans, which binds to the HR1 region of a variety of coronaviruses to form a hetero-hexamer. Thus, it competitively inhibits the formation of hexamers from the HR1/HR2 interaction of the virus itself, thereby inhibiting the fusion of the virus with the host cell and preventing the entry of the virus’ genes into the cell for replication. After the outbreak of COVID-19, they demonstrated that EK1 peptide can also bind to the HR1 of SARS-CoV-2 and efficiently inhibit the invasion and infection of SARS-CoV-2 into host cells.^461^ Currently, the EK1 nebulizer is about to enter a Phase IIa clinical trial. Besides, by modifying with palmitic acid or cholesterol, Jiang/Lu et al. have further developed a series of more efficient and broad-spectrum lipopeptide fusion/invasion inhibitors, such as EK1C4, EKL1C, and EK1-C16, which are equally effective in inhibiting the infection of various SARS-CoV-2 variants without being affected by variants.^462,463^

In addition to the HR1 region, the HR2 region is also an important target for the development of viral fusion/invasion inhibitors. However, the HR1-derived peptides contain too many hydrophobic amino acids to maintain a stable α-helix structure in aqueous solution. Therefore, they designed a protein inhibitor targeting the HR2 region of SARS-CoV-2, 5-helix (composed of two HR2 and three HR1-derived protein fragments), which can bind to the HR2 region of SARS-CoV-2 to form a heterologous six-helix bundle and inhibit the invasion of the virus into host cells. Like the EK1 peptide, nCoV-5-helix can also effectively inhibit the infection of various SARS-CoV-2 variants, including Omicron and Delta.^464^ Compared with common neutralizing antibody drugs, peptide-based inhibitors have the following obvious advantages: (1) Peptide-based inhibitors act on the conserved HR1/HR2 locus, which allows peptide-based inhibitors to demonstrate broader-spectrum COVs inhibitory activity than targeting RBD/NTD-neutralizing antibodies; (2) Peptide-based inhibitors have shorter amino acid sequences and smaller molecular weights, which can be directly synthesized by chemical synthesis, greatly reducing the time and production cost; Moreover, it can directly inhibit the replication of COVs in the patient’s lung by intranasal administration. (3) high safety and efficacy, basically equal to antibody drugs. The only drawback is that the half-life of peptide drugs is shorter than that of antibody drugs. (4) Peptide-based inhibitors are more stable and can be stored for a long time at room temperature, with lower storage and transportation costs. Therefore, the development of Pan-CoV fusion/invasion inhibitors can effectively prevent and treat SARS-CoV and its variants, SARS-CoV and MERS-CoV infections.

HY3000 was developed in the early stage of the COVID-19 outbreak. Based on the analysis of the membrane fusion mechanism of SARS-CoV-2, SARS-CoV,MERS-CoV.^465–467^ Gao et al. designed a polypeptide inhibitor P3 targeting SARS-CoV-2 HR1, which can inhibit theinfection of SARS-CoV-2,^467^ but peptide P3 has insufficient activity. In order to further improve the activity of P3 peptides and develop anti-SARS-CoV-2 polypeptide membrane fusion inhibitors, researchers designed many P3 derivatives based on their structure, finally, they found that the antiviral activity of P315 peptide was the best, which was about 8-fold higher than that of P3. Furthermore, through PEG and cholesterol modification, the P315V3 lipopeptide with about 1000-fold higher activity was obtained (HY3000).^468^ It can bind to the HR1 region of the S protein of SARS-CoV-2 and inhibit membrane fusion to prevent the virus from entering the cell interior. At the same time, the hydrophobic tail of HY3000 can be fixed to the surface of the cell membrane, forming a protective barrier at the cell surface. Besides, it can effectively inhibit novel COVs variants, including XBB.1.5. In addition, it can inhibit SARS-CoV, MERS-CoV and seasonal COVs HCoV-NL63, HCoV-229E and HCoV-OC43, HY3100 shows a broad spectrum of COVs. Based on the effective antiviral effect, HY3000 nasal spray was approved by NMPA for phase I clinical trial in August 2022. Now, the phase I clinical trial has been completed and the drug shows enough safety. Based on it, it is currently under clinical phase II trial in China. Furthermore, HY3000 was accepted by the US FDA clinical trial in January 2023.

As mentioned above, targeting the most conserved HR1 region of SARS-CoV-2 can be an important target for the development of broad-spectrum viral fusion/invasion inhibitors. By using the membrane fusion mechanism, researchers developed a peptide YKYY017 (from SARSCoV-2 HR2-deriving lipopeptides, IPB20)^468,469^ that targeted the conserved HR1 region and competitively bound to the viral HR1 region, then YKYY017 prevents the formation of the viral homologous six-helix bundle (6-HB) structure, and blocks the fusion process between the virus and host cells. It can inhibit two membrane fusion pathways, cell surface and endosomes, to exert antiviral effects. In vitro pharmacodynamic studies showed that YKYY017 had significant and broad-spectrum inhibitory effects on the original SARS-CoV-2 strain and its epidemic variants (Delta, Omicron BA.1, BA.2, BA.4 and BF.7). YKYY017 has a broad spectrum against cold-causing coronaviruses (HCoV-NL63, HCoV-229E, etc.), severe acute respiratory syndrome coronavirus (SARS-CoV), Middle East respiratory syndrome coronavirus (MERS-CoV), and bat-derived coronavirus RaTG13), pangolin-derived coronaviruses (PCoV-GD, PCoV-Gx), it has also shown significant inhibitory effects, reflecting the broad-spectrum anti-coronavirus activity. Toxicological studies showed that YKYY017 had no obvious toxicity and related adverse reactions, as well as a good therapeutic and preventive effects. It is currently in Phase II/III clinical trial in China (ChiCTR2300075467) and was approved for clinical investigation on May 12th, 2023. As a new generation of COVs membrane fusion inhibitor with broad spectrum, YKYY017 aerosol inhalation is expected to be well used in the fight against SARS-CoV-2 and its variants.

Recently, Dang et al. developed a new broad-spectrum SARS-CoV-2 fusion inhibitor, A1L35HR2m-Chol, which can effectively inhibit different SARS-CoV-2 mutant strains, as well as SARS-CoV and MERS-CoV.^470^ The researchers interconnected the ACE2-derived peptide A1 with the N-terminus of the HR2m peptide via a flexible junction (GGGGS)7 of the appropriate length to generate A1-(GGGGS)7-HR2m (A1L35HR2m), (HR2mL35A1) was then obtained by exchanging the positions of the A1 peptide and the HR2m peptide. In addition, two other fusion peptides were constructed by shortening the length of the junction to derive A1L5HR2m and A1HR2m. The researchers then evaluated the ability of A1L35HR2m, HR2mL35A1, A1L5HR2m, and A1LHR2m to inhibit SARS-CoV-2 by the SARS-CoV-2 pseudotyped virus infection assay. It was found that the addition of ACE2-derived peptide A1 to the N-terminus of HR2m peptide with a long flexible junction significantly increased the anti-SARS-CoV-2 activity.

This research revealed that the antiviral mechanism of A1L35HR2m may be similar to that of HR2-derived peptides, which can enhance the competitive interaction with the affinity of HR1 structural domains to block the fusion of viruses with target cell membranes. Meanwhile, adopting the S protein-mediated cell-cell fusion assay, it was found that A1L35HR2m-Chol effectively inhibited SARS-CoV-2 D614G S protein-mediated cell fusion between 293 T/EGFP/S and Caco-2 cells, which contributes to the enhancement of the viral activity; moreover, the researchers also performed site-specific modification of the C-terminus of A1L35HR2m to generate A1L35HR2m, which is an anti-viral peptide that can be used to prevent the virus from fusing with the target cell membrane. The results showed that A1L35HR2m-Chol exhibited broad and effective inhibitory activity against different SARS-CoV-2 VOC, SARS-CoV and MERS-CoV, and did not show in vitro or in vivo toxicity.As well, the level of A1L35HR2m-Chol dose had no effect on the hepatic and renal functions of the mice.

In general, peptides against acute respiratory distress syndrome (ARDS) caused by coronaviruses and related respiratory problems caused by SARS-COV-2 infection, most of them are target-based synthetic peptides. Researchers have been working on the development of peptide therapeutics for a variety of diseases, including Covid-19, due to their ease of synthesis, high target specificity, selectivity, and low toxicity, etc. In addition, a peptide-based Covid-19 vaccine is in clinical trials. Finally, a number of novel peptide drug delivery systems with high potential for other disease development. Peptide drugs are expected to be further developed in the future.

### Advance of peptide-based therapeutics in digestive diseases

The use of peptide drugs in gastrointestinal diseases is mainly focused on the control of acute upper gastrointestinal bleeding (such as somatostatin analogues and vasopressin analogues) and other intestinal diseases (Linzess^®^, Trulance^®^).Vapreotide (Sanvar^®^), a synthetic somatostatin analogue, was approved in Mexico in 2004.^471,472^ In addition, a phase III clinical trial found that the combination of Vapreotide and endoscopic therapy was more effective than endoscopic therapy alone in controlling acute bleeding in patients with cirrhosis and variceal bleeding.^473^ Terlipressin (Terlivaz^®^) is a synthetic vasopressin analogue developed by Ferring Pharmaceuticals, which can be used to treat gastrointestinal variceal bleeding. In terms of anti-gastrointestinal bleeding, the main focus is on bleeding caused by cirrhosis.^474^ It was approved by the FDA in 2022 to improve the rapid decline in renal function in adult patients with hepatorenal syndrome (HRS).^475^

In addition, peptides have been shown to be effective in irritable bowel syndrome (IBS). Irritable bowel syndrome (IBS) is a chronic functional gastrointestinal disease characterized by smooth muscle dysfunction, which is prone to relapse and has a high incidence worldwide. “IBS is characterized by persistent or intermittent episodes of abdominal pain, bloating, and abnormalities in bowel habits and stool form, without morphological or biochemical abnormalities.” Linaclotide (Linzess^®^) is a guanylate cyclase-C (GC-C) agonist, which is a 14-amino acid polypeptide.^476,477^ Linzess^®^ was approved by the FDA in December 2012 for the treatment of adult patients with constipation-predominant irritable bowel syndrome (IBS-C) and chronic idiopathic constipation (CIC). It is also the first new prescription drug approved for the treatment of adult patients with moderate to severe IBS-C in Europe. Linzess^®^ and its active metabolites have been shown to bind to guanylate cyclase-C (GC-C) receptors on the luminal surface of the small intestinal epithelium, leading to the activation of GC-C and the increase of intracellular and extracellular concentrations of cyclic guanosine monophosphide (cGMP). Intracellular cGMP increases the secretion of chloride and bicarbonate in the small intestine by activating the cystic fibrosis transmembrane conductance regulator (CFTR), which eventually leads to increased secretion of intestinal fluid and accelerated intestinal transport. Through GC-C activation, linaclotide reduced intestinal pain and increased gastrointestinal transit speed in animal models. Extracellular cGMP can reduce the activity of painful nerve fibers and alleviate visceral pain in animal models.^478,479^

In January 2017, the FDA approved Trulanc^®^ (plecanatide), a GC-C (guanylate cyclase-C) agonist with linaclotide, for CIC (Chronic Idiopathic Constipation).^479,480^ Plecanatide is as effective as linaclotide in the treatment of CIC but not as effective as linaclotide in the treatment of IBS-C.^481^ In terms of structure, Plecanatide and Linaclotide are both small molecule monopeptide, but with one less disulfide bond, which is easier to synthesize. However, Plecanatide has a higher dosage than linaclotide in the indication of IBS, which is expected to be further optimized in future studies.

Elsiglutide is an orally active and selective GLP-2 receptor agonist, which is an analogue of GLP-2. It can increase cell proliferation, reduce intestinal cell apoptosis, and regulate intestinal balance.^482,483^ In a rat model, Elsiglutide ameliorated Lapatinib (HY-50898) -induced diarrhea in rats. In addition, clinical phase II studies testing elsiglutide in colorectal cancer (CRC) patients receiving chemotherapy (CT) induced diarrhea with 5-fluorouracil (5-FU) have not yet yielded results.^484^ Therefore, peptide drugs also occupy a certain proportion in the field of digestive tract diseases.

### Advance of peptide-based therapeutics in Alzheimer’s disease

Alzheimer’s disease (AD) is a multifactorial neurodegenerative disease with insidious onset, clinically characterized by memory impairment, aphasia, loss of recognition, visuospatial skills impairment, behavioral changes and other comprehensive dementia manifestations and more serious neurological impairments, which seriously affects the patient’s quality of life, and in severe cases even death.^485^ It is estimated that by 2050, this number will reach 130 million. This will create a huge social burden.^486^ Worse still, the main challenge in the treatment of Alzheimer’s disease is that its pathogenesis has not been fully clarified, and the more recognized hypotheses of the pathogenesis include deposition of β-amyloid (Aβ)^487^ and intracellular Tau protein aggregates,^488^ neuroinflammation^489^ and lack of acetylcholine (Ach), oxidative stress,^490^ and bio-metal dyshomeostasis, etc.^491^ The Aβ deposition hypothesis suggests that the normal mechanism of Aβ removal from the brain tissue of AD patients is disrupted, leading to the accumulation of toxic Aβ in the brain tissue to form Aβ plaques,^492^ and when the accumulation reaches a certain level, it triggers a series of neurodegenerative processes, such as inflammation, oxidative stress, and the deposition of Tau proteins,^493^ which ultimately leads to a series of clinical conditions of AD. The establishment of the Tau protein deposition hypothesis is inextricably related to the Aβ hypothesis, which suggests that Tau protein as an important protein involved in stabilizing neuronal structures in the brain and regulating internal neuronal transport systems. Tau protein plays an important role in normal brain cell activity.^494^ However, due to the Aβ deposition mentioned in the above hypothesis, Tau protein accumulates and forms abnormal protein clumps (NFTs), which severely disrupts the normal function of neuronal activity and ultimately leads to a series of AD disorders in patients. In addition, Aβ accumulations in neurons can also activate glycogen synthase kinase 3-β (GSK-3β), which phosphorylates Tau proteins and ultimately forms NFT,^495^ making the clinical symptoms of AD appear gradually. At the same time, neuroinflammation exacerbates the pathological processes of Aβ accumulation, Tau accumulation and Tau phosphorylation,^496^ and the pathological processes of the three hypotheses produce a synergistic effect, which leads to a persistent vicious cycle, thus exacerbating the disease process of neuronal damage. In addition, the hypothesis of reduced acetylcholine levels is also recognized as an important cause of AD. In patients with AD, cholinergic neurons in the brain degenerate, resulting in reduced Ach levels,^497^ which in turn leads to a range of clinical symptoms of AD, including reduced learning and memory abilities. Currently approved therapies include cholinesterase inhibitors (rivastigmine, donepezil, tacrine and galantamine), and NMDA receptor antagonists, but even their combination provides only temporary relief of symptoms, not a cure.

The key clinicopathologic indicators of AD are amyloid plaques formed by amyloid accumulation in extracellular regions or hyperphosphorylated Tau proteins aggregated in intracellular regions, which form neural protofibrillary tangles (NFTs) in affected neurons. Amyloid plaques mainly contain amyloid β40/42 peptides, which further accumulate leading to neuroinflammation and mishandling of Tau protein.^498,499^ Based on this, a large number of domestic and international researchers have tried to develop drugs that can remove these two toxic proteins from the brain in the hope of treating AD or slowing down the process.^500^ Among them, Aβ is produced by hydrolyzing and processing amyloid precursor protein (APP) by β-secretase (BACE1) and γ-secretase, which means that inhibition of β- and γ-secretase cleavage can inhibit the production of Aβ protein. In this regard, many drugs targeting secretase have entered clinical trials,^501^ including CTS-21166 (CoMentis), PF-05297909 (Pfizer), LY2886721 (Lilly), and AZD3293 (AstraZeneca). The results of the clinical phase 1 trial showed that CTS-21166 reduced plasma levels of Aβ protein^502^ 665, and AZD3293 was also shown in the clinical phase 2/3 trial. AZD3293 also achieved the target efficacy in clinical phase 2/3 trial.^503^ However, the results of Phase II/III clinical trials for many other related new drug developments have been unsatisfactory.

Peptides can intervene in the process of Aβ aggregation in a variety of ways, and thus researchers continue to plow through the field. Driven by advances in drug design and synthesis techniques, a common strategy for peptides used in the treatment of AD is to design peptides with high affinity to bind to Aβ and prevent its aggregation. These peptides can alter the conformation of Aβ or inhibit its aggregation process by interacting with it. Phages can search for potential therapeutic peptides for various diseases based on the affinity between the produced peptide and the target molecule, so researchers have used it to search for peptides with the potential to inhibit Aβ aggregation. Kiessling et al.^504^ then identified several Aβ-affinity peptide ligands by phage display.^505^ Another strategy is to block amyloid or peptide production by designing peptides with the core sequence of the Aβ fibrillization to suppress protein or peptide aggregation. In 2015, Prof. Kapurniotu of the Technical University of Munich, Germany^506^ designed a series of peptide-based small molecule inhibitors based on the protein sequences of islet amyloid polypeptide (IAPP), a type of small molecule inhibitor that can inhibit the root causes of amyloid proteins (amyloid- beta peptide and IAPP) aggregation, thus reducing the cytotoxicity of amyloid fibrils, and providing a new idea for the treatment of Alzheimer’s disease in the clinic. Prof. Kapurniotu further deepened his research on this basis,^507^ and developed a new type of cyclic peptide-based small-molecule inhibitor based on the straight-chain peptide-based small-molecule inhibitors. The optimized cyclic peptide-based small molecule inhibitor not only has high efficiency and specificity in binding and recognizing amyloid proteins, but also shows a high degree of protein hydrolysis stability in human plasma, and can cross the human blood-brain barrier in cellular models, making it a potentially effective anti-amyloid drug for the treatment of AD. In addition, chiral biomolecules have the advantage of inherent stereoselectivity, whereby the aggregation of Aβ can be hindered by modifications of chiral amino acids or peptides. Amino acid chirality can determine the folding of the peptide backbone, hydrogen bonding interactions, and even the biological functions of proteins in vivo.^508^ Xue et al. borrowed graphene to structurally modify a peptide to make it pharmaceutically active and regulate Aβ40 aggregation in vitro.^509^

Intracellular neuroprogenitor fiber tangles containing phosphorylated Tau proteins are a hallmark protein of AD, and hyperphosphorylation of Tau proteins causes the deposition of this characteristic protein. Meanwhile, Tau function is regulated by a variety of post-translational modifications at more than 50 sites, and Tau in healthy neurons carries multiple phosphorylation motifs that are located predominantly in its microtubule assembly domain. It has also been shown that Aβ can initiate a deleterious cascade of responses involving Tau pathology and neurodegeneration, *i.e*., Tau proteins are mediators of Aβ cytotoxicity.^510^ Possible synergistic effects of Aβ and Tau proteins on microglia and astrocytes, whose interactions mediate cognitive dysfunction in AD patients. Researchers have proposed multiple mechanisms of action against Tau, including specific removal of pathological Tau species, reduction of Tau production, promotion of Tau physiological function through microtubule stabilization or inhibition of post-translational modifications.^511^ However, most of the Tau protein-targeting therapies tested to date have been immunotherapies that can target Tau proteins intracellularly and/or extracellularly, but targeting extracellularly alone is unlikely to be effective,^512^ and no Tau-targeting therapies have yet to show definitive clinical efficacy in preclinical or early-stage AD. From the perspective of the aberrant aggregation of toxic Tau proteins, Zheng jie et al. designed and synthesized a peptide chimera drug, DEPTAC, which can specifically reduce the phosphorylation level of Tau proteins and thus promote their degradation to avoid aggregation.^513^ This drug can selectively recruit protein phosphatase 2 A (PP2A) to the periphery of Tau proteins and thus induce their dephosphorylation.

Several studies have shown that neuroinflammation plays a prominent role in the pathogenesis of Alzheimer’s disease. There is a correlation between neuroinflammation and amyloid and Tau pathology. When innate immune cells are activated, programmed cell death can be induced through a variety of pathways, and cell death usually leads to the release of pro-inflammatory cytokines that propagate the innate immune response and can eliminate beta plaques and aggregated Tau proteins.^514^ However, chronic neuroinflammation caused by cell death is associated with neurodegenerative diseases and may worsen Alzheimer’s disease. The continued elucidation of cell death pathways and central innate immune sensor signaling pathways involved in regulating neuroinflammation and Aβ/Tau clearance will have a major impact on the field of AD research. Recent evidence suggests an association between AD and T2DM.^515^ Numerous reports have found that GLP-1 RAs improve cognitive behaviors and pathological features in AD patients and animals, which may be related to the improvement of glucose metabolism in the brain. GLP-1 RAs not only reduce Aβ deposition by inhibiting Aβ production and facilitating its clearance, but also reduces inflammatory mediator. GLP-1 RAs also exert neuroprotective effects by inhibiting oxidative stress and reducing neuronal apoptosis.^515^ In addition, GLP-RAs can improve the cognitive function of AD patients by enhancing learning memory and spatial orientation.^516^

Though strides have been made, peptides still face some challenges in the treatment of AD at present. First, AD is a complex disease whose pathogenesis and pathological processes have not been fully clarified, so further in-depth studies are needed to identify the best therapeutic targets for peptide drugs. Second, the stability, pharmacokinetics and bioavailability of peptide drugs also need to be addressed to ensure that the drugs can effectively exert therapeutic effects in vivo.

Looking ahead, with a deeper understanding of the pathogenesis of AD and the continuous advancement of drug development technology, peptide drugs will have a broader prospect in the treatment of AD. More peptide drugs targeting AD may enter clinical trials in the future and are expected to provide new and more effective treatment options for AD patients. Meanwhile, research on the combined application of peptide drugs with other therapeutic methods also needs to be strengthened, with a view to achieving better therapeutic effects.

### Advance of peptide-based therapeutics in rare diseases

Currently, there are approximately 7000 identified rare diseases. Although it is individually rare, it generally affects a significant portion of the general population (10% of the population). However, less than 6% of all rare diseases have approved treatment options, highlighting their huge unmet need for drug development.^517,518^ Rare diseases (RDs) were often chronic, leading to lifelong disability or early death; many RDs had pediatric onset, and about 30% of children with RDs died before age 5 years.^519^ 70% of rare diseases are hereditary, caused by germline and somatic gene mutations.^520^ A small number of rare diseases are also caused by environmental, infectious or immunologic factors (e.g., African trypanosomiasis),^521^ but these will not be discussed here.

Peptides also play an important role in the treatment of rare diseases. Due to the diversity and complexity of the etiology of rare diseases, conventional drugs have limited therapeutic effects on their treatment. However, some specific peptide drugs, such as synthetic analogs or biosynthetic peptides, may have specific therapeutic effects on certain rare diseases. For example, certain rare genetic diseases may result in abnormal hormone levels, while other peptide drugs are able to regulate these hormone levels, thereby alleviating the symptoms or slowing down the progression of the disease. Therefore, the research and application of peptide drugs in the treatment of rare diseases has also attracted much attention and is expected to provide more effective treatment options for these patients. Here we focus on more recent developments.

#### Rett syndrome

Rett syndrome, originally proposed by Andreas Rett in 1966, is a rare progressive neurodevelopmental disorder that results in severe intellectual disability, loss of mobility, and autism-like symptoms, other features include slowed head growth, seizures, autistic features, and respiratory abnormalities.^522,523^ Trofinetide (Daybue^®^) is a novel synthetic analog of the amino-terminal tripeptide of insulin-like growth factor I (IGF-1) that can be administered orally and is neuroprotective at minimal doses.^524,525^ It was approved by the FDA in March 2023 for the treatment of Rett syndrome for children 2 years of age and older, Notably, it is the first and only FDA-approved treatment for Rett syndrome.^526^ Studies have shown that trofinetide not only inhibits inflammatory cytokine production and overactivation of microglia and astrocytes, but also increases the amount of available IGF-1 that can bind to the IGF-1 receptor, which is beneficial for the treatment of core symptoms of Rett syndrome.^524^

#### Generalized myasthenia gravis (gMG)

Generalized myasthenia gravis (gMG) is a disorder of neuromuscular junction transmission caused by the destruction of the postsynaptic membrane of skeletal muscle by autoantibodies at the neuromuscular junction.^527,528^ The clinical manifestations of generalized myasthenia gravis (GMG) are fluctuating skeletal weakness and fatigue intolerance, which aggravate after activity and alleviate after rest. Local or general weakness is the main symptom. In severe cases, it involves respiratory muscles and causes respiratory failure, which is potentially fatal. Zilbrysq^®^ is a novel macrocyclic peptide of a C5 complement inhibitor that can be used to treat adult patients with generalized myasthenia gravis (gMG) with positive anti-acetylcholine receptor (AChR) antibodies.^526^ It is also the first gMG targeting C5 complement inhibitor that requires only a once-daily subcutaneous injection, providing a simpler dosing option for patients with generalized myasthenia gravis. Zilucoplan (Zilbrysq^®^), a cyclic peptide drug, was approved by the FDA in October 2023 for the treatment of AChR antibody-positive gMG that fails to respond to other immunosuppressive therapies.^529,530^ As a complement C5 inhibitor, zilucoplan inhibits the complement mediated neuromuscular junction injury by targeting the mechanism of action. Different from the monoclonal antibody C5 inhibitor, zilucoplan, as a peptide, can be used with intravenous immunoglobulin and plasma exchange at the same time without supplementary administration, which is a new type of peptide drug with extremely simple administration.

#### Short bowel syndrome (SBS)

Short bowel syndrome (SBS) is mostly caused by large area surgical resection of the small intestine or congenital bowel diseases. In this condition, the absorption capacity of small intestine is limited, which cannot meet the needs of normal growth and development of patients, and parenteral nutrition (PN) support is required.^531,532^ Clinically, the most common disorders leading to SBS in adults are Crohn’s disease, mesenteric ischemia, radiation enteritis, postoperative adhesions, and postoperative complications. The common causes of SBS in children include congenital diseases such as necrotizing enterocolitis, volvulus, gastroschisis and intestinal atresia.^533^ Nutritional therapy is a very important treatment for SBS, and parenteral nutrition is very important for the survival of infants and children with SBS. However, the related complications caused by long-term PN may endanger the life of SBS children, and more effective drugs are urgently needed to meet the clinical needs.^534,535^

GLP-2 is a specific growth factor with intestinal protective effect, which can enhance the transport of glucose and lipid in intestinal cells, enhance nutrient absorption and promote intestinal adaptation.^536^ Teduglutide is a GLP-2 analogue that was previously approved by EMV in 2012 for the treatment of short bowel syndrome and malabsorption. In February 2024, Teduglutide received NMPA approval for the treatment of short bowel syndrome in adults and children aged 1 year and older. The substitution of alanine at position 2 by glycine makes teduglutide resistant to degradation by dipeptidyl peptidase-4 (DPP-4). Thus, tidulutide has a longer half-life than GLP-2. Tidulutide can reduce the requirement of PN in SBS children, increase the tolerance of enteral nutrition (EN), help some children achieve intestinal autonomy, and has good safety and tolerance, which provides a new treatment option for SBS children suffering from PN.^537^ In addition, the duration of PN is positively correlated with the incidence of catheter-related infections. The present study proves that GLP-2 plays a role in intestinal adaptation. GLP-2 is a peptide secreted by intestinal L cells after foodintake.^538,539^ It can prevent the apoptosis of intestinal epithelial cells, thereby promoting the growth and recovery of intestinal tissue. This process of growth and repair is essential for children with SBS to achieve intestinal autonomy, which provides a new hope for reducing the dependence on PN.^540,541^

In addition, a number of peptide drugs have been approved for the treatment of other rare diseases. For example, Etelcalcetide (Parsabiv^®^), a calicomimetic agent, was approved by the FDA in February 2017 for the treatment of secondary hyperparathyroidism in adults with chronic kidney disease (CKD) undergoing hemodialysis.^542^ Glatiramer was approved in 1996 for the treatment of multiple sclerosis (MS), an immune-mediated inflammatory demyelinating disease of the central nervous system.^543,544^ The common clinical manifestations are recurrent vision loss, diplopia, limb sensory disturbance, limb movement disorder, ataxia, bladder or rectal dysfunction, etc.). In June 2023, glatiramer acetate (Copaxone^®^) was officially approved in China for the treatment of adult patients with relapsing multiple sclerosis (MS). In addition, glatiramer acetate (Copaxone^®^) can also be used for the treatment of Rett syndrome in a phase II clinical trial (NCT02153723).^545^ Other therapies include pegcetacoplan (a C3 complement inhibitor, approved by the FDA in May 2021) for paroxysmal nocturnal hemoglobinuria, and terlipressin (an AVPR1A agonist, approved by the FDA in September 2022) for hepatorenal syndrome (HRS).^546^

### Advance of peptide-based therapeutics in orthopedic metabolic diseases

The application of peptide drugs in orthopedics is mainly focused on the treatment of osteoporosis. Among them, osteoporotic fracture (OPF) is the most serious symptom of osteoporosis, which is difficult to heal, has a high disability rate and difficult to heal, and poses a serious threat to the health of middle-aged and elderly people. Osteoporosis can be treated in two ways. On one hand, it reduces bone loss by inhibiting bone resorption; on the other hand, it decreases bone loss by increasing the number or activity of osteoblasts. However, the use of anti-bone resorption drugs alone does not restore lost bone structure. Increasing the number or activity of osteoblasts may be a more attractive approach to enhancing bone formation and promoting bone regeneration.^547^

Nowadays, there are many drugs to promote bone formation, including monoclonal antibodies to sclerostin and recombinant human parathyroid hormone drugs, parathyroid hormone-related protein (PTHrP) analogs, such as Evenity^®^ (romosozumab), Forteo^®^ (teriparatide) and Tymlos^®^ (abaloparatide).^548–551^ Evenity^®^ has the potentialside effect of increasing the risk of heart attack, stroke or death due to cardiovascular disease. Parathyroid hormone drugs have drawbacks such as a two-year use limit and a potential risk of osteosarcoma. Therefore, new strategies and methods are urgently needed for the research and development of drugs for bone formation. YLL3 and YLL8 are “osteogenic specific” peptides discovered by Yao et al. through the OBOC (One Bead One Compound) library. YLL3 and YLL8 not only have high affinity for osteogenic progenitor cells, but also activate the phosphorylation of the pro-survival factor Akt. In vitro experiments confirmed that YLL3 and YLL8 can increase the differentiation and maturation of osteoblasts. Moreover, YLL3 and YLL8 can target endogenous osteogenic progenitor cells for bone regeneration therapy.^547^

In addition, Osteogenic Growth Peptide (OGP) is also very effective in preventing osteoporosis. OGP is a peptide composed of fourteen amino acid residues which is a promoter of systemic response to bone marrow injury. Osteogenic growth peptide and its C-terminal pentapeptide OGP (10-14) can up-regulate the mRNA expression of type I collagen, bone calcium and alkaline phosphatase in osteoblast-like cells.^552^ At the same time, it can significantly increase the content of collagen, osteocalcin, calcium and alkaline phosphatase activity of cells, promote bone formation and inhibit bone resorption, increase the number of osteoblasts, and reduce the number of osteoclasts, so as to prevent osteoporosis.

### Advance of peptide-based therapeutics in migraine disease

Migraine is a periodic neurological disorder in which patients often experience unilateral, throbbing recurring headaches accompanied by nausea, vomiting, light sensitivity, sound sensitivity, and other symptoms that last from hours to days. Migraine seriously reduces the quality of life and work of patients, and it even has a significant economic and social impact. The International Headache Society categorizes migraine into three types: migraine without aura, migraine with aura and chronic migraine.^553^ Migraine without aura is the most common type, accounting for 70 to 90 percent of cases.

Treatment of migraine includes medication and non-medication. For acute migraine, medication is the main treatment. Commonly used medications include painkillers, tricyclic antidepressants, calcium channel blockers, etc., which are used to relieve headaches and control the frequency of attacks. Despite the wide range of medications available, their efficacy is not satisfactory, and many others are difficult for patients to adhere to because of side effects. This is related to the fact that the exact cause of migraine is still not fully understood. In recent years, clinical models have identified key signaling molecules involved in migraine, including calcitonin gene-related peptide (CGRP), pituitary adenylate cyclase-activating peptide 38 (PACAP-38), and nitric oxide, whose exposure significantly increases the risk of migraine attacks. Among these molecules, calcitonin gene-related peptide (CGRP) is a vasodilatory neuropeptide that plays a crucial role in the pathophysiology of migraine and is a promising target for migraine therapy.^554^

Monoclonal antibodies targeting CGRP have demonstrated migraine prevention in multiple Phase II and Phase III clinical trials, and their long-term effects in key regions provide stable CGRP blockade beyond existing prevention methods. Monoclonal antibodies targeting calcitonin gene-related peptide (CGRP) and its receptor have opened a new era in migraine prevention.^555^

We refer to those that antagonize CGRP receptors as gepants, which have a strong affinity for CGRP receptors and can prevent other molecules from binding to them, blocking signaling. The advantage over other types of drugs is that they do not constrict blood vessels.^556^ Early studies of generation gepants relieved migraine symptoms, although their preventive effect on the disease has yet to be studied. However, development of the first generation of gepants has also been halted for various reasons, such as the discontinuation of ocegepant due to low oral bioavailability, and the banning of tecapant telcagepant and MK-3207 due to hepatotoxicity after frequent use.^557,558^

Despite these safety concerns in the initial studies of first-generation gepants, the efficacy of gepants has prompted further efforts to develop safe CGRP-blocking molecules. Three second-generation gepants continue to be in clinical development: Rimegepant, Ubrogepant, and Atogepant. Rimegepant passed the clinical phase 2b trial based on its superior efficacy in treating acute migraine. This was followed by a Phase 3 trial (NCT02867709, NCT02828020), which was randomized, double-blind and placebo-controlled, which provided preliminary evidence of the findings of Phase IIb trial.^559^ In addition, safety studies were conducted concurrently. In the Phase 2b/3 study of atogepant, the drug was also found to reduce the number of migraine days per month in patients compared to placebo.^560^

In December 2019, the U.S. Food and Drug Administration, approved the first gepant drug, ubrogepant (Ubrelvy^®^), for the acute treatment of migraine in adults. Aquipta^®^ (atogepant) was approved by the EMA in August 2023. It is also the first and only once-daily oral calcitonin gene-related peptide (CGRP) receptor antagonist (gepant) therapy in the European Union for the prophylactic treatment of chronic and episodic migraine. As an oral small molecule CGRP receptor antagonist, it is competitive, highly selective, and highly effective. In addition, atogepant not only prevents vasodilation, but also relieves or prevents migraine by preventing CGRP-induced neurogenic inflammation, injurious transmission, and various functions of CGRP. In a phase 3 double-blind trial, researchers randomly assigned adults with 4-14 migraine days per month in a 1:1:1:1 ratio to receive either a once-daily oral antimigraine agent (10 mg, 30 mg, or 60 mg) or a placebo for 12 weeks,^561^ and the data demonstrated that the combination of the agent taken orally once daily was effective in reducing the number of migraine days and headache days.^562^

Launched in September 2022, Nurtec ODT^®^ (rimegepant) is approved in several countries, including the US and the EU, for the acute treatment of migraine with or without aura in adults and the prophylactic treatment of episodic migraine in adults. Structurally, it contains a cyclohepta[b]pyridine core, and due to the poor water solubility of the pre-developed BMS-846372 ( < 2 μg/mL), the team fitted Rimegepant with a primary amine, which resulted in a better potency and greatly increased water solubility of this drug (50 μg/mL). In vitro, Rimegepant effectively antagonized both the CGRP receptor and the insulin 1 (AMY 1) receptor, but was about 30 times more effective at blocking the CGRP receptor.^563^ It is effective not only for acute migraine,^564,565^ but also for prophylactic treatment.^566^

Launched in March 2023, zavegepant is a third-generation small-molecule calcitonin gene-related peptide (CGRP) receptor antagonist developed by Pfizer Roots for the prevention and treatment of chronic and episodic migraine. It is the first nasal CGRP antagonist.

By comprehensively analyzing journals and patents, the researchers summarized the common structural features of CGRP receptor antagonists and synthesized the new compound. Although this molecule effectively inhibited CYP3A4, it had poor solubility. A SAR study of the side chain of benzothiophene identified 7-methylindazole as capable of enhancing activity and moderately inhibiting CYP3A4. By adding a fluorine atom at the C-8 position of the quinazolinone, the molecule BMS-694153 was synthesized, which showed a substantial increase in solubility, but low oral bioavailability. The researchers replaced the oxidation-sensitive benzylidene methylene group with an electron-deficient sp2 hypomethyl group. At the same time, a simple SAR of the piperidinopiperidine side chain to N-methylpiperidinyl-piperazine (with two protonatable nitrogens) yielded BMS-742413 (i.e., the marketed drug zavegepant), with a polar surface area of 116.18 Å2, which further reduced binding to serum proteins. In addition, the crystalline compound was surprisingly water-soluble, sufficient to support nasal administration of the drug ultimately developing zavegepant.

Several randomized, double-blind controlled trials have shown that zavegepant (Zavzpret^@^) is effective in the treatment of acute migraine with or without aura. For example, one clinical study found that a significantly higher percentage of patients in the zavegepant (Zavzpret^@^) group experienced relief from the worst symptoms of headache and MBS 2 hours after taking the drug compared to the placebo group. Specific data showed that headache relief was 23.6% in the zavegepant group compared to 14.9% in the placebo group, and MBS relief was 39.6% in the zavegepant group compared to 31.1% in the placebo group. In addition, 12.4% of patients in the zavegepant group continued to feel pain relief 48 hours after taking the drug, compared with only 8.7% in the placebo group. These studies provide ample evidence for the use of zavegepant as an effective anti-migraine therapy.^567^

## Advance of peptide-based vaccines

### Overview of peptide-based vaccines

Since the 18th century, from the eradication of smallpox to the current global COVID-19 pandemic, vaccines have played a critical role in protecting humans and livestock from infectious diseases.^568^

Through ongoing research into immune mechanisms and advancements in vaccine delivery technologies, there are currently six categories of vaccines in use or under development^569^: live attenuated vaccines, inactivated vaccines, subunit vaccines, toxoid vaccines, viral vector-based vaccines, and nucleic acid (DNA or RNA) vaccines. Traditional vaccines typically contain either live attenuated or inactivated bacteria.^570^ Live attenuated vaccines and inactivated vaccines directly replicate natural infection to elicit host immunity. However, they pose risks such as the reversion of vaccine strains, potential issues with protein expression, contamination during the expression process, difficulties in pathogen cultivation, and the risk of inducing autoimmunity and excessive inflammatory responses in humans.^571^ Additionally, diseases with complicated immune evasion mechanisms, such as malignancies and CMV infections, pose challenges for the production of standard attenuated live or inactivated vaccines.^572–575^

In the field of subunit vaccines, subunits encompass membranes, capsules, toxins, polysaccharides, proteins, or small peptides. Here, our discussion of peptide-based vaccines refers to subunit-based peptide vaccines.^576,577^ The peptides utilized in these vaccines may originate from tumor antigens or may be synthetic peptides designed to mimic them.^578^ Peptide-based strategies employ minimal epitopes, providing an opportunity to select pathogen-specific protective sequences that do not cross-react with human tissues.^3^ This makes peptide vaccines an attractive approach in vaccine development. Peptides have a defined chemical structure and simple construction, ensuring safety and reproducibility since they do not require folding into a tertiary structure. The ability to lyophilize and store peptide vaccines in solid form at room temperature further distinguishes them from other vaccine types. Additionally, peptide vaccines can be specifically modified to reduce the risk of adverse reactions and side effects.^579^ The key features of peptide vaccines are as follows^580–582^: (1) precise characterization, allowing for quality control comparable to small molecule drugs; (2) lack of pathogenic sequences, lowering the risk of allergies or autoimmune reactions; (3) customization and synthesis of specific epitopes as needed^583^; and (4) low production costs and controllable processes, facilitating commercialization. Certainly, like other vaccines, peptide vaccines also have some limitations, such as the potential for immune escape and low immunogenicity in single-peptide vaccines.^584^ However, the continuous advancements in new technologies and discoveries bring new hope for the development of improved and optimized clinically effective peptide vaccines in the future.

**Fig. 11:**
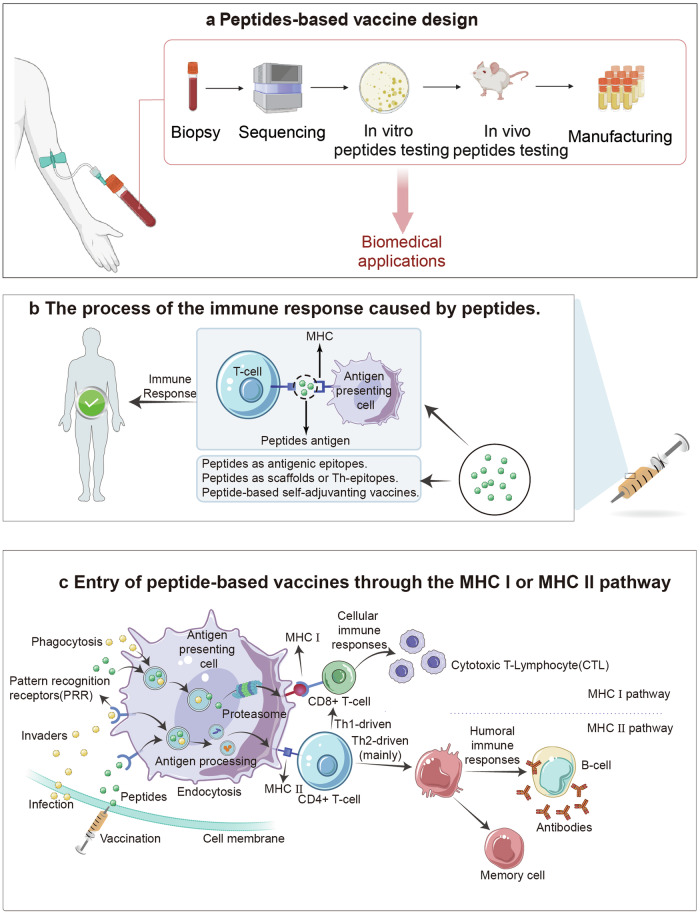
The design process of peptide-based vaccines and their mechanisms for generating immune responses. **a** Schematic diagram of peptide-based vaccine design; (**b**) The process of causing an immune response; (**c**) Entry of peptide-based vaccines through the MHC l or MHC ll pathway

Peptide-based vaccines have multiple design processes and corresponding mechanisms for generating an immune response (Fig. 11). The initiation of the immune response begins with dendritic cells (DCs) as the first step: DCs incorporated as part of the therapeutic vaccine take up exogenous antigens and differentiate into mature immunogenic DCs (Fig. 11a). The second step involves the activation of specific T-cell responses: in lymphoid organs, peptide-loaded DCs induce specific T-cell responses (Fig. 11b). Finally, these activated T cells migrate to the target site and exert their effects (Fig. 11c). Currently, no peptide-based vaccines have been approved for market use. Table 7 presents the clinical trial status of peptide vaccines. Of the 16 peptide vaccines in Phase III clinical trials, only 2 are for treating immune diseases (IR-902 (NeuroVax), CV-MG01 (Myasterix)). The remaining vaccines are anticancer vaccines targeting hormone refractory prostate cancer, melanoma/metastatic cancer, acute myeloid leukemia, among others. It is clear that cancer is a prominent research area for peptide vaccines. The selection of antigens, as well as the choice of adjuvants and delivery systems, are crucial for translating peptide vaccines from the laboratory to the clinic. This paper focuses on the research progress of tumor peptide vaccines and their delivery methods.

**Table 7:** Peptide-based vaccines in clinical trial phase III

No.	Clinical Trial ID	Name	Targets
1	NCT02057159	IR-902^815^ (NeuroVax)	Forkhead Box P3 (FOXP3)
2	JPRN-jRCT2080222153	ITK-1^816^	NA
3	NCT00094653	MDX-1379^817^	HLA-A2
4	NCT04229979	galinpepimut-S^818^	Wilms Tumor1 (WT1)
5	NCT05155254	IO-102/IO-103^819^	Indoleamine-Pyrrole 2,3 Dioxygenase (IDO)
6	NCT05232916	GLSI-100^820^	Granulocyte-Macrophage Colony-Stimulating Factor (GM-CSF)
7	JPRN-UMIN000016954	Asudemotide^821^ (S-588410)	NA
8	NCT03200821	G17DT Immunogen(Insegia)^822^	NA
9	NCT01479244	nelipepimut-s^823^ (NeuVax)	Human Epidermal Growth Factor Receptor 2 (HER2)
10	NCT02049151	tecemotide^824^	NA
11	JPRN-UMIN000007279	OCV-C01^825^	NA
12	NCT03165435	CV-MG01^826^ (Myasterix)	NA

### Peptide-based anti-tumor vaccines

Tumor cells or lysates contain a variety of endogenous components and malignant chemicals, making them unsuitable for standard paradigms such as attenuated or inactivated vaccines. Instead, tumor-specific antigens must be screened and identified before being biologically engineered into recombinant proteins or other specialized vaccinations. Peptide vaccines based on specific epitopes are a promising strategy in oncology.^582^ Tumor immunotherapy has become the fourth clinical therapeutic option for cancer, following surgery, chemotherapy, and radiation therapy.^585^ Tumor vaccines work by stimulating the body’s immune system to attack and remove tumor cells.^586^ In 2010, Sipuleucel-T, the first dendritic cell-based tumor vaccine, was approved by the FDA for the treatment of prostate cancer.^587^ Later, various tumor vaccines have been tested in clinical trials, such as dendritic cell (DC) vaccines, gene vaccines (RNA/DNA), and peptide-based vaccinations.^586,588^

Peptide-based tumor vaccines offer a safe and precise therapeutic strategy to cancer treatment. Within the body, they are captured, internalized, and processed by antigen-presenting cells (APCs), activating immune responses via both classical and non-classical HLA-mediated pathways. The first peptide vaccination for melanoma treatment began clinical trials in 1995,^589^ targeting the MAGE-1 antigen using a nine-peptide sequence (EADPTGHSY). When APCs collect antigens, HLA presents them to MAGE-1-positive T cells, triggering an immunological response against melanoma. Unfortunately, there have been no Phase III trials reported. Optimal antigen selection is critical for the success of peptide vaccines. Ideal antigens should have tumor specificity and be easily recognized by T cells to stimulate immune responses. Tumor antigens primarily fall into three categories: tumor-associated antigens (TAAs) (overexpressed in tumor cells, low expression in normal tissue), cancer/testis antigens (CTAs) (expressed in tumors and testes), and neoantigens such as tumor-specific antigens (TSAs).^590^ The first melanoma peptide targeted the cancer/testis antigen (CTA) is MAGE-1. A list of antigens that are currently in phase III clinical trials for peptide targeting is presented (Table 7), and to maintain objectivity and balance, we have excluded trials with undisclosed data from this list. The current Phase III clinical trial antigens mainly include HLA-A2 (single antigen, NCT00094653), Wilms Tumor1 (single antigen, NCT04229979), and Indoleamine-Pyrrole 2. Three clinical trials are currently ongoing: NCT05155254 for 3 Dioxygenase (multiple antigens), NCT05232916 for Granulocyte-Macrophage Colony-Stimulating Factor (single antigen), and NCT01479244 for Human Epidermal Growth Factor Receptor 2 (HER2) (single antigen). TAAs are also expressed in normal tissues. Under immune surveillance, the restriction of specific high-affinity T cell clones can lead to immune tolerance or provoke strong autoimmunity. This warrants discussion regarding the less-than-ideal outcomes in clinical trials, as highlighted in the article “Antitumor Peptide-Based Vaccine in the Limelight”. The emergence of TSAs brings new hope. TSAs are expressed exclusively in tumor cells and not in normal cells. They are not subject to immune tolerance restrictions, which can induce robust immune responses. TSAs originate from cancer-associated viruses, such as HPV and HBV, or mutated proteins in tumor cells. Clinical trials are underway for HPV vaccines alone or in combination with ICB.^591–594^ Tumor progression results from dysregulated mutations in oncogenes and tumor suppressor genes.^595–598^ These mutations, which include individual point mutations and gene rearrangements, as well as shared mutations across multiple tumors (such as EGFRvIII), provide opportunities for customizing TSAs.^593^ Additionally, post-translational modifications can enrich the TSA repertoire, such as phosphorylation, lactylation, and glycosylation.^599,600^ Compared to peptide vaccines targeting TAA, TSA vaccines offer superior immunogenicity, but their translation remains a challenge. First, antigen detection and determination are time-consuming and labor-intensive.^601^ Currently, protein elution from human leukocyte antigen combined with peptide identification by mass spectrometry is used. However, advances in whole exome sequencing and bioinformatics have been used to identify individual mutations, predict novel antigens and their peptide ligands, and assess peptide-HLA affinity.^602–605^ Since TSA arise from mutation-prone tumors, they are prone to secondary mutations during treatment and face the risk of antigen loss,^606^ which means that peptide vaccines may offer a promising solution. For example, a patient with metastatic castration-sensitive prostate cancer was treated with an HLA I and HLA II-matched peptide vaccine, resulting in robust and durable CD4+ and CD8 + T cell responses.^607^ Marquez-Manriquez and colleagues predicted and designed a twelve-peptide vaccine against six recurrent proteins in epithelial ovarian cancer (EOC) patients that stimulated T-cell responses and effectively prevented patient relapse,^608^ greatly increasing researchers’ confidence. Peptide vaccines can activate multiple T-cell responses to avoid immune evasion, while in combination with other direct cytotoxic strategies, they can promote additional antigen spreading, amplify sustained and robust immune responses, enhance therapeutic efficacy and prevent immune suppression.^609–611^

Clinical success of peptide-based tumor vaccines depends on adjuvants. Exceptional immunostimulants and ideal delivery strategies are critical to the ultimate clinical translation of peptide-based vaccines. Adjuvants and delivery systems intersect in vaccine development, serving the common functions of protecting peptide vaccines from degradation, assisting in peptide delivery to APCs, promoting their maturation to activate immune responses, and accelerating effector T cell homing to target sites. Traditional adjuvants such as incomplete Freund’s adjuvant (IFA) have limited ability to activate immune responses, with an objective response rate of only 2.6% (440 patients).^612^ Immunostimulants currently approved for clinical use include aluminum salts (such as aluminum hydroxide and aluminum phosphate), MF59 (an oil-in-water emulsion adjuvant), AS04 (an adjuvant containing aluminum salt and monophosphoryl lipid A), CpG 1018,^613–616^ and others. Research on novel immunostimulants continues, exploring avenues such as viral vectors, TLR4 agonists and various new materials.^588,617,618^ A continued deepening of the understanding of immune response mechanisms, supported by new technologies and materials, will facilitate the transition of peptide-based vaccines from the laboratory to the clinic.

## The cutting-edge technological landscape of peptide drugs

### Display library technology

Display library technology is a high-throughput biological technique that is primarily used to rapidly screen for peptides or antibodies that have affinity for specific targets or antigens. The basic principle of display library technology is to construct a large library containing many different peptide or antibody sequences. These sequences are typically designed randomly to cover the possible combination space. The encoding sequences (DNA or mRNA) for each peptide or antibody molecule are connected to their surface display. This library is then incubated with the target antigen or molecule of interest. Peptides or antibodies with affinity will bind specifically to the target. The bound peptides or antibodies can then be separated out, and the sequences of the binding molecules obtained by analyzing their connected encoding sequences. Based on the screening results, key sequences or motifs of the binding peptides or antibodies can be identified.^47,619,620^ Overall, display library technology can efficiently screen for peptides or antibodies with specific binding to a given target from a huge sequence library within a short time frame. This greatly shortens the traditional biological screening process and has important application value in drug development and biological detection reagents.

Currently reported peptide display library technologies mainly include phage display technology, bacterial display technologies, yeast display technologies, mammalian cell display technologies, and cell-free display technologies.^619^ These technologies all utilize biological macromolecules (proteins, DNA, RNA, etc.) to display peptide sequences on surfaces, rapidly obtaining peptides with affinity for specific targets, greatly facilitates identification of original peptides for a wider range of targets.

#### Phage display

Phage display technology is an important peptide screening platform, first developed by Smith et al. in 1985, and now widely used for the discovery of peptides related to various diseases.^621^ This technology utilizes phage libraries displaying random or designed peptide libraries on their surfaces, and screens through repetitive “biopanning” processes to obtain peptide sequences with high affinity for the target molecule.^622^ The “biopanning” process includes incubating the phage library with the target molecule, washing, eluting, and amplifying bound phages. After multiple rounds of panning, phage clones with higher binding affinity for the target can be enriched.^619,623^ Due to its unique advantages, phage display has become a preferred platform for discovering many disease-related targeting peptides, and plays an important role in cancer,^624–626^ inflammation,^627,628^ brain diseases,^629,630^ blood diseases,^631^ and other research fields.

As early as 2009, a bicyclic peptide-based phage display strategy was developed. Bicyclic peptides have a unique structure stabilized into bicyclic form by chemical scaffolds, avoiding reaction with phage cysteines.^632^ Up to now, a number of peptides and antibodies produced by in vitro phage display are either authorized or in advanced phases of clinical development.^633^ Nowadays, in vitro phage display techniques have advanced. For instance, Hampton et al. constructed bicyclic peptide phage display libraries using highly selective biparatopic reagents, and used them to screen new SARS-CoV-2 inhibitors.^634^ Subsequent studies showed bicyclic peptides can be used to construct high-capacity phage libraries to combine innovative inhibition mechanisms, providing new modalities for antiviral drugs.^635^ In addition, recent studies constructed bicyclic peptide libraries with stereochemical diversity, and identified a set of novel bicyclic peptides with submicromolar MYC binding affinities, validating their bioactivities in human cancer cells.^636^ Nevertheless, traditional in vitro methods fall short in increasingly demanding scenarios when the goal is to produce best-in-class compounds against challenging targets.^637^ To meet clinical demands, in vivo phage display, where screening is performed in living organisms, has become increasingly popular. Compared to in vitro screening, in vivo screening can select candidate peptides with desired specificities, pharmacokinetics, and stability in complex biological environments, greatly improving the possibility of screening high-affinity peptides under physiologically relevant conditions.^637^ For example, LIU et al. used phage display technology combined with a novel biological screening program to obtain a peptide CLP002 (WHRSYYTWNLNT) that specifically binds to the residues of PD-L1 and PD-1 interaction and blocks the interaction of PD-1/PD-L1 in tumor cells.^638^ Similarly, an EGFR specific binding peptide GE11 (YHWYGYTPQNVI) from a phage display peptide library was reported, which competitively inhibits the binding of endogenous growth factors.^639^ Besides, da Fonseca Alves et al. developed a new type of electrochemical biosensor using phage display technology by screening for specific recombinant peptides to successfully distinguish serum samples from breast cancer patients and benign breast disease patients.^640^ In addition, Kim et al. used the phage display peptide library Ph.D.-12 to screen out a targeting peptide PA2 that specifically binds to the outer membrane protein OprF of Pseudomonas aeruginosa. They constructed a hybrid peptide GNU7-PA2 with the antimicrobial peptide GNU7, which showed effective antimicrobial activity against P. aeruginosa without toxicity to the host.^641^ The specific peptides screened out by phage display technology can be used not only therapeutically but also for diagnosis. For instance, the MAK30 peptide can serve as a probe to detect bacterial resistance to bacitracin by binding to the innovative phage display APBA-dimer library APBA-dynamic covalent warheads. This rapid and accurate detection method helps timely assess bacterial resistance and provide more precise medication guidance in clinics.^628^

#### Bacterial display

Bacterial display is a technique that utilizes bacterial cell surfaces to present peptide libraries. Common carrier strains include Gram-negative bacteria such as E. coli and Pseudomonas.^642^ Specifically, the gene encoding the peptide sequence is fused to a gene encoding a bacterial surface protein (e.g. LamB, OmpA).^643^ The fusion protein expressing the peptide is then displayed on the bacterial surface. Screening against targets of interest can identify peptides with binding affinity.^644^ Bacterial display has broad applications in screening antibody epitopes, receptor peptides, enzyme substrates, etc. It can also be used for protein engineering, vaccine development, biosensing and other areas.^645^ Recently, the archaeal peptide display system (RAD display) has been used to screen neutralizing peptides against SARS-CoV-2 antigens, demonstrating important application prospects in the design of antigens for peptide-based vaccines.^646^ Compared with phage display, advantages of bacterial display include easier operation, faster speed and lower cost. However, as the carrier is a prokaryotic organism, the displayed peptides are limited to bacterial surface expression, restricting further functional verification. In addition, low display efficiency also limits its application.^647^ It is worth noting that there have been recent reports of using epPCR and single-cell sorting to rapidly enhance bacterial surface display.^648^

#### Yeast display

Yeast display technology utilizes the cell surface of yeast to present peptide libraries. The commonly used yeast includes Pichia pastoris, Saccharomyces cerevisiae, etc.^619^ The method is to fuse the gene encoding the peptide sequence to the gene encoding the yeast surface protein (e.g. Aga2p) and express the fusion protein. Then the library is screened against the target to obtain peptides with affinity.^649,650^ Saccharomyces cerevisiae display technology, the most frequently used yeast display technology, has been utilized to map antibody-binding sites, design ideal interfaces between proteins, and evolve novel enzymes.^651,652^ A promising method was recently reported for preparing an oral vaccine against H7N9 influenza in which the hemagglutinin (HA) of A/Anhui/1/2013 (AH-H7N9) was utilized as a model antigen and displayed on the surface of the Saccharomyces cerevisiae EBY 100.^653^

Human yeast display technology is another emerging platform using humanized yeasts to present human-derived peptides and proteins, holds great potential. Many human genes encoding proteins can successfully replace the corresponding genes in yeast without compromising growth, enabling this controllable system.^654^ With benefits like high transformation efficiency, large library capacity, and biosafety, human yeast display allows measuring human protein activities in a simplified organismal environment. Thus, it overcomes the limitations of immunogenicity associated with non-human display systems and facilitates discovering new human biology.^654,655^ An illustration of this field is a platform utilizes humanized yeast display technology to express human MHC molecules on the yeast surface, which enables rapid and accurate screening of MHC-restricted T cell epitope peptides from emerging pathogens. The efficient and accurate identification of DR4 ligands from the SARS-COV-2 spike protein as a model antigen demonstrates the high efficiency and accuracy of this method.^656^ In a recent update, a method called “peptide display” has been reported, in which a second-generation yeast display approach to assess peptide-MHC class II binding was developed. The method decouples peptide and MHC-II expression by displaying a peptide library on the yeast surface while expressing MHC-II proteins as soluble recombinant molecules, successfully eliminating the need for yeast-specific MHC optimization and increasing the scale of MHC-II alleles that can be utilized in yeast display screening.^657^ Generally, yeast display allows detection of protein folding and post-translational modifications. However, it is relatively low throughput. Human yeast display overcomes immune compatibility issues, while retaining advantages like eukaryotic folding. But the technology is still in its early stages.

#### Mammalian cell display

Mammalian cell display utilizes the cell surface of mammalian cells like HEK293, CHO, etc. to present peptide libraries.^658^ The method is to fuse the gene encoding the peptide with a transmembrane protein gene (e.g. PDGFR) and transfect mammalian cells to express the fusion protein on the cell surface.^659^.Mammalian cell display is especially suitable for designing humanized antibodies, studying protein-protein interactions, and developing vaccines.^660^ Taking HEK293 cell display as an example, it has been applied to select antibody fragments, identify novel B cell epitopes, and engineer proteins.^661^ Besides, a CHO cell line platform suitable for mammalian cell display generated by integrating a Bxb1 landing pad into a commercially available Flp-In CHO host has been recently reported. This platform has been validated for generating antibody display libraries containing up to tens of millions of variants and differentiating antibodies based on biophysical properties through mutagenesis and selection.^662^ In contrast, advantages of mammalian cell display include high diversity libraries and mammalian-derived peptides, but the transfection efficiency is lower.

#### Cell-free display

Cell-free display technologies do not rely on living cells as carriers; instead, the peptide or protein library is linked to a genetic molecule like mRNA or cDNA, which acts as both the genotype and phenotype. In vitro transcription and translation are used to synthesize the peptide and form the linkage with the genotype without using living cells. Common techniques include ribosome display, mRNA display, cDNA display, and CIS display.^663^ Ribosome display utilizes stabilized ribosome-mRNA-polypeptide complexes to display peptides. During reverse transcription, the mRNA is held on the ribosome to form a peptide-mRNA complex.^664^ The most recent report of this is the use of ribosome display using rolling circle amplification to create homologous multivalent libraries to screen for low-affinity protein binders.^665^ mRNA display works by covalently linking a peptide and its corresponding mRNA to form a peptide-mRNA complex that allows screening for peptides with specific properties by affinity selection. For example, by combining co- and post-translational library diversification strategies, a circulating library with reactive dehydroalanine (Dhas) was created to screen for valent cyclic peptide inhibitors in mRNA display.^666^ Similarly, cDNA display forms peptide-cDNA complexes by linking peptides and their corresponding cDNA during reverse transcription and PCR to screen peptides. Recently, a customized human fetal brain cDNA phage display library was reported, and four maternal antibodies against the UH-ASD antigen were successfully screened, providing a new tool for the diagnosis of autism spectrum disorder (ASD).^667^ Besides, there is also CIS display technology that enables directly connected DNA templates and the development of peptides, greatly simplifying and extending the selection process. The fundamental idea of this technology is that it makes use of the DNA replication initiation protein’s (RepA) cis-activity, which allows it to attach its own DNA template with specificity.^663^ An earlier application of this method was to screen VEGFR-2 binding agents,^668^ but there have been rare reports in recent years.

Overall, the primary constraint on all cell-based display technologies we mentioned above is library size, which influences the toxicity of displayed molecules to host cells as well as the effectiveness of DNA transformation. Amplification bias also affects cell display investigations, whereby little changes in growth rate can have a major effect on the total variety of the library and result in the loss of some affinity molecules when transformation.^619^ Cell-free display technologies have significant advantages in this regard, advantages of which include direct genotype-phenotype linkage, large library size, no transformation limitation, and ability to use unnatural amino acids.^619,663^ However, in vitro protein synthesis can be inefficient. There are also challenges in library construction, genotype-phenotype stability, and quantitative screening. But cell-free systems continue to be improved for peptide engineering applications.

#### Outlook

Moving forward, augmenting efficacy and overcoming size constraints will be key goals for phage and bacterial display systems. For yeast and mammalian cell display, increasing screening throughput is paramount. Optimizing genotype-phenotype stability and quantitative screening will drive innovation in cell-free display. As these technologies continue to progress, their capacity for engineering novel peptides and proteins will expand. By leveraging the strengths of each approach, display platforms hold great promise in generating new biological discoveries.

### Application of deep learning in peptide discovery and modification

Peptide research, encompassing the study of short chains of amino acids, holds immense therapeutic potential due to the ability of these biologically active molecules to precisely target specific sites within the body.^2,669^ However, the discovery and development of novel peptide drugs has historically been a challenging and time-consuming process.^670^

**Fig. 12:**
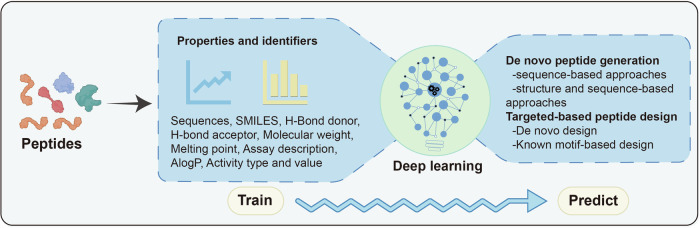
A flowchart depicting the application of deep learning in peptide drug design

The advent of techniques like deep learning (DL) has revolutionized this landscape by providing innovative tools to accelerate and enhance peptide drug design.^671–673^ By analyzing vast datasets, these models can rapidly identify promising peptide candidates and predict optimal amino acid sequences and structures for desired therapeutic properties (Fig. 12).^671,674^ The integration of DL with peptide research has led to a synergistic approach that transforms the drug discovery process through computational methodologies.^675^ These platforms utilizing convolutional neural networks, recurrent neural networks, attention mechanisms and other algorithms can guide peptide synthesis for increased efficiency,^676–678^ facilitate rational modifications to enhance bioavailability and therapeutic effects, and expedite the exploration of more possibilities with therapeutic peptides.^679,680^

#### Application of deep learning in peptide research

The biological activities of peptides are intrinsically tied to their structural properties, hence, elucidating peptide structures and designing novel peptides is therefore critical for understanding biological mechanisms and developing new therapeutics.^670,681^ Traditionally, techniques like molecular docking and molecular dynamics have been used to predict peptide structures by posing the problem as an energy minimization.^682^ However, these methods are computationally demanding, exhibit limited accuracy, and require experimental validation.^683^ To tackle these long-standing challenges, DL models can predict peptide structures and design novel peptides with higher throughput and accuracy compared to traditional techniques.^671,676^ Application of DL in the synthesis and design of peptides mainly includes de novo peptide generation and targeted-based peptide binder design.

##### De novo peptide generation

In de novo peptide generation, models like Recurrent Neural Networks (RNN),^684^ Long Short-Term Memory (LSTM) networks,^685^ Variational Autoencoders (VAE),^686^ and Generative Adversarial Networks (GAN)^687^ are trained on existing peptide data. They discern intricate patterns within sequences, with a focus on generating new peptide sequences during design. There are two main methods for de novo peptide generation: sequence-based approaches and structure and sequence-based approaches. In the field of sequence-based approaches, models such as RNN^684^ and LSTM^685^ networks are employed. These models treat peptide design as a translation task, using neural language models to generate novel amino acid sequences or map source peptides to target peptides. Structure and sequence-based approaches integrate sequence and structural information, which significantly enhances peptide design, imbuing peptides with specific structural characteristics that contribute to their functionality and offering more effective peptide generation. In this field, models like GANDALF and HelixGAN have been reported, adeptly merging textual sequence information with structural details.^688^ Advanced techniques like Wasserstein bidirectional GANs generate structurally plausible full-atom helical structures was also reported.

##### Targeted-based peptide binder design

Designing peptide binders that target specific proteins or biomolecules is another essential tactic for creating peptides capable of modulating key interactions. Usually known as targeted-based peptide binder design, this method also involves two primary strategies involved: de novo design and known motif-based design.^676^ De novo design predicts interaction patterns and affinities between peptides and target proteins, effectively deducing amino acid sequences compatible with desired functionality and structure to discover new peptides.^689^ Using AlphaFold to create de novo design models of novel macrocyclic peptides is a typical example of this method. In contrast, motif-based design relies on existing research data, incorporating key motifs from known peptide sequences or structures to bolster binding affinity.^690^ The most noteworthy progress in this area is the newly described strategy for creating peptides that bind to certain locations on target proteins’ surfaces devoid of any information.^691^

#### Future perspectives and conclusion

In summary, advances in computing power, data, and algorithms have set the stage for DL to revolutionize peptide research. Demonstrated successes in accelerating tasks like synthesis and bioactivity prediction underscore its capacity, while the growing integration of artificial intelligence into pharmaceutical workflows exemplifies accelerating adoption of these techniques.^692–694^ As demand rises for novel peptide therapeutics, the field faces both challenges and promising solutions.^695^ To step forward, integrating big data and advancements in DL is indispensable for accelerated drug design. In detail, constructing large benchmark datasets for training, along with independent validation sets, is critical to enhance reliability and aid experimentalists; incorporating structural or evolutionary information can also improve model robustness.^696,697^ Besides, exploring nuanced sequence representations and descriptors presents opportunities for developing architectures tailored for peptides, which could improve model accuracy.^676,698^ Looking forward, advances in computing are enabling techniques like deep learning to accelerate and empower peptide discovery. The synergy between biology and data science promises to overcome challenges and realize the full potential of peptide therapeutics.

### The current status and progress of oral peptide drug delivery technologies

Though peptide drugs are widely employed in the treatment of various diseases,^2^ the administration of peptide drugs has always faced enormous challenges, mainly in the following aspects: 1) Biological barriers such as pH changes in the gastrointestinal tract, degradation by proteolytic enzymes, obstruction by intestinal epithelial cells, and the first-pass effect, which make it difficult for peptide drugs to be administered orally.^699^ 2) Inherent physicochemical defects such as short half-lives and poor stability, which require peptide drugs to be injected multiple times a day to maintain therapeutic levels, causing inconvenience and suffering to patients.^700^ 3) Limitations of mucosal administration such as poor tissue permeability (hydrophilicity), enzymatic activity of mucosal macrophages, and rapid clearance in the nasal and respiratory tracts, resulting in very low bioavailability of peptide drugs administered via the mucosal route.^701^ 4) Risks of interactions such as immunogenicity, increased in vivo toxicity or reduced efficacy, which affect the safety and efficacy of peptide drugs. These challenges severely hamper the oral administration of peptide drugs.^702^ Therefore, parenteral administration is currently the most commonly used route of administration, with subcutaneous injection being the most widely used.

In order to overcome the challenges of administering peptide drugs, researchers have been exploring novel drug delivery systems and technologies while optimizing subcutaneous administration, and have achieved rapid developments especially in the field of oral delivery. These novel drug delivery systems and technologies can not only improve the in vivo stability and bioavailability of peptide drugs, but also achieve sustained or controlled release to significantly enhance their therapeutic effects. We will first review the current status of subcutaneous administration, and then focus on the latest research progress in the development of oral peptide drugs (Table 8).

**Table 8:** Approved oral peptide drugs and their oral delivery strategies

Peptide	Name	Company	Indication	Strategy	Approval
Cyclosporin A	Neoral®	Novartis Pharma AG	Immunosuppression after organ transplantation	Lipid-based microemulsion containing lipids, surfactant, and co-solvent	1997
	Sandimmune®				1990
Desmopressin acetate	DDAVP®	Ferring Pharmaceuticals SA	Diabetes insipidus	Chemical modification cyclization	1978
Voclosporin	Lupkynis®	Aurinia Pharmaceuticals Inc.	Systemic lupus erythematosus nephritis		2021
Octeotide	Mycapssa®	Chiasma Inc.	Acromegaly	Oily suspension	2020
Semaglutide	Rybelsus®	Novo Nordisk Pharma AG	T2DM	SNAC as absorption enhancer	2019

#### 6.3.1. Subcutaneous administration

As of 2021, five GLP-1 RAs and one insulin analog have been commercialized for subcutaneous delivery systems. Although these drugs have achieved significant effects in diabetes management, their dosages are limited by subcutaneous delivery, and injection site reactions are common adverse effects of GLP-1 RAs (especially Bydureon). Therefore, many researchers are devoted to developing and improving novel subcutaneous formulations to extend drug circulation time, increase drug safety, improve patient compliance, enable the possibility of self-administration at home, thereby reducing medical costs.^703^ In 2018, the Subcutaneous Drug Delivery and Development Consortium was established, pointing out several major challenges facing optimization of subcutaneous delivery of biotherapeutics, including the need for technological innovations to achieve delivery of high-dose/volume formulations, addressing the insufficient bioavailability of subcutaneous formulations, and eliminating concerns over the higher immunogenicity of the subcutaneous route compared to the intravenous route.^704^ Currently, research on subcutaneous delivery systems is mainly focused on polymeric micro/nanosystems, in situ hydrogels/fibers, ultrasound-triggered systems, etc.^705^ Studies have shown that polymeric micro/nanosystems based on FDA-approved poly (lactic-co-glycolic acid) (PLGA), poly (lactic acid) (PLA) or chitosan have commercialization advantages. Through structural modifications, they can not only construct sustained/controlled release systems to improve bioavailability, but also act as vaccine adjuvants to enhance efficacy.^706^

In situ hydrogels/fibers have environment-sensitive and molecule-responsive characteristics, and good biocompatibility, which are a promising alternative to traditional subcutaneous delivery systems.^707^ The application of innovative materials can effectively improve the bioavailability of peptide drugs. Through structural modifications that impart glucose-responsive capabilities, controlled release of insulin can be achieved, avoiding side effects like hypoglycemia.^708^ Unlike systems relying on endogenous stimuli such as pH and glucose levels, ultrasound-triggered systems are controlled by exogenous active signals to release drugs on demand. Unlike endogenous passive responses, ultrasound-responsive insulin release systems have the features of being active, visualizable, precise, and providing sustained release. A single injection can effectively control blood sugar for up to 3 days. Such systems have gradually become a hot research direction in subcutaneous delivery.^709,710^

#### Oral administration

Due to the ease of degradation, poor absorption, and low bioavailability of peptide drugs in the gastrointestinal tract, frequent subcutaneous injections or other invasive administration methods are required, which brings inconvenience and suffering to patients. Therefore, the development of safe, effective and convenient oral peptide drugs has always been a hot research topic in the pharmaceutical field.^711^

**Fig. 13:**
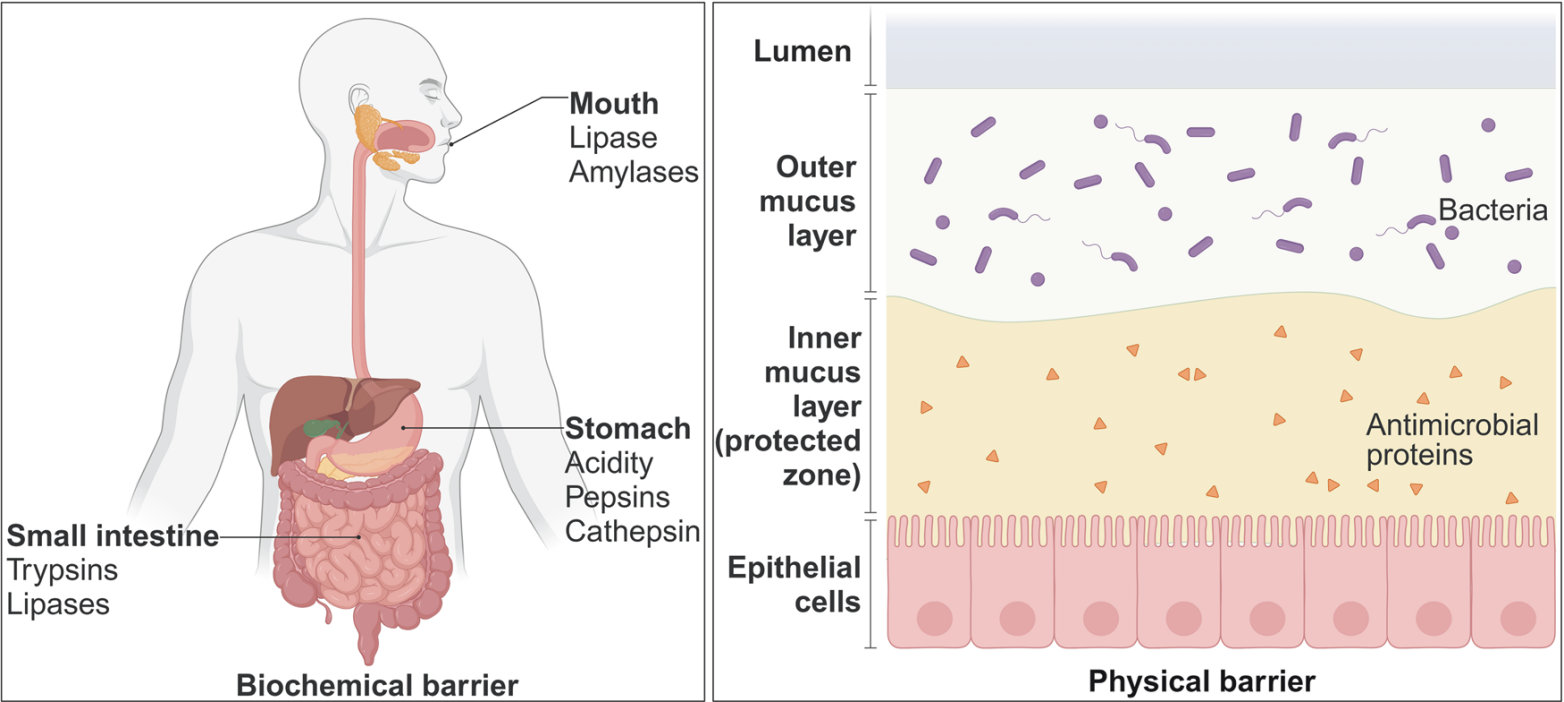
The barriers to the oral absorption of peptide drugs mainly include biochemical and physical barriers. Initially, the physiological conditions of the gastrointestinal system led to the degradation of peptide medicines, rendering them challenging to absorb. Besides, a highly complex mucus layer covering the surface of the gastrointestinal system will considerably inhibit the absorption of peptide medicines. Figure 13 was created with biorender.com

However, oral delivery of peptide drugs has always been an enormous challenge. In order to achieve oral delivery of peptide drugs, researchers have to overcome various biochemical and physical barriers in the gastrointestinal tract (Fig. 13). Firstly, peptide drugs encounter digestion by enzymes in the oral cavity, stomach and intestines, which break down the peptides into smaller molecules, resulting in loss or reduction of their activity, requiring frequent administration. Secondly, peptide drugs have to penetrate the mucus layer covering the intestinal epithelium, which is a physical barrier, as its negative charge and glycoprotein components impede drug permeation.^712^ Thirdly, peptide drugs have to pass through the tight junctions between epithelial cells, which is also a physical barrier, as they restrict paracellular or transcellular permeation of drugs. Finally, after entering intestinal cells, peptide drugs may still be metabolized by cytochrome P450 enzymes or pumped out by P-glycoproteins, thus reducing their bioavailability.^713^ To address these challenges in oral delivery of peptide drugs, protect them from destruction in the GI tract, increase their intestinal absorption and bioavailability, and prolong their in vivo half-life, the current mainstream strategies include structural modification and pharmaceutical formulation improvements.

In the field of structural modification, the methods attempted so far cover D-amino acid substitution,^714^ lipidization,^50,231^ cationization,^715,716^ PEGylation,^717^ cyclization etc.,^718,719^ which help improve the stability and permeability of peptide drugs. For example, by substituting the 8th L-arginine with D-arginine and removing the amino group from the first amino acid, an orally available desmopressin acetate (DdavpR) was successfully obtained. In addition, PEGylation and cyclization have received much attention in recent years. Conjugating PEG covalently to a drug can increase its solubility, reduce immunogenicity, prolong circulation time in vivo, thereby improving bioavailability. This method has been widely used in polypeptide and protein drugs, especially for oral delivery. For example, the PEGylated insulin analogue Tregopil developed by Indian company Biocon has completed phase III clinical trials. Recently, it was reported that surface modification of reverse micelle lipid nanocapsules (RM-LNC) with DSPE-PEG-FA (FA-RM-LNC) can increase the mucus permeability and intestinal absorption of exenatide, significantly improving its absorption efficiency in the small intestine.^720^ In addition, cyclization can improve the oral absorption and stability of peptides by adding a fatty acid chain linker to the C-terminus of the peptide chain. Among the marketed oral peptides, three are cyclic peptides – cyclosporine A, voclosporin and desmopressin. The most well-known cyclic undecapeptide cyclosporine A (CsA) capsule formulation (SandimmuneR) was approved by the FDA in 1990, with an oral bioavailability as high as 25-30%.^721^ In January 2021, the trans-isomer analog of CsA called voclosporin (LupkynisR) was also approved for marketing for the treatment of lupus nephritis. Recently, an interesting de novo design approach combining computational design and experimental characterization for membrane-permeable and orally bioavailable peptides has been reported.^722^

In terms of pharmaceutical formulations, various pharmaceutical formulation improvement strategies including enhancing drug stability, promoting mucus permeation or adhesion, increasing epithelial permeability, carrier-mediated transport, designing sustained and controlled release formulations, and developing targeted delivery systems have also made significant progress. Some technologies have been successfully applied to marketed oral products. Strategies such as enteric coating, enzyme inhibition,^723^ permeation enhancement,^724,725^ and composite mixtures of hydrogels,^726,727^ show promise for enabling oral delivery of other peptide drugs like exenatide and insulin. Some of these formulations are currently undergoing clinical trials.^728^ Among them, the most noteworthy strategy so far is co-formulating peptides with permeation enhancers to achieve oral delivery.^58,724,729^ For example, the stability and absorption of semaglutide (Rybelsus®) and somatropin through cell membranes were improved after oral co-formulation with C18 fatty acid or the chemical sodium N-[8-(2-hydroxybenzoyl)amino] caprylate (SNAC).^730^ In addition, an oral capsule formulation of octreotide containing medium chain fatty acid salts and polyvinylpyrrolidone (PVP) as suspending agents has also been achieved. It was also reported that a tricyclic peptide targeting PCSK9 (a key regulator of plasma LDL-cholesterol) attained 2.9% oral bioavailability in cynomolgus monkeys when co-administered with permeation enhancers.^731^ Besides, some other peptide drugs are also exploring the potential for oral delivery through technologies such as micro/nanotechnology, targeted delivery systems, and physical devices. For example, Nicholas J. Hunt et al. reported insulin-conjugated silver sulfide quantum dots coated with a chitosan/glucose polymer, achieving dual pH/glucose responsive oral delivery of insulin for sustained and controlled release. Results showed that the insulin distributed to the liver in animals and dose-dependently reduced blood glucose without hypoglycemia or weight gain and adverse effects in mice, rats and non-human primates.^732^ Additionally, some physical devices like microneedles, patches and iontophoresis/ultrasound have also been investigated for oral delivery of peptides, piercing or stimulating the oral cavity or intestine to increase local absorption. These devices can utilize biodegradable materials such as polylactic acid, chitosan and gelatin to avoid issues with retention and removal. The advantages of microneedles and patches are reduced systemic exposure and adverse effects of peptides, but there are also some challenges like complex manufacturing, size limitations, and ensuring stability. Iontophoresis/ultrasound physically increases intestinal permeability through electric fields or sound waves, and can be co-administered with peptide drugs to enhance their intestinal absorption. The advantage of this non-invasive method is oral delivery, but its safety and tolerance to the intestine needs to be considered.^733^

In summary, oral delivery of peptide drugs is a field with enormous potential and challenges that involves interdisciplinary crossover and integration, requiring continuous innovation and optimization. With the development of new materials, technologies and methods, more breakthroughs and progress will be made in oral peptide drug research, providing better options and solutions for treating various diseases. According to a recent report by Allied Market Research, the global oral polypeptide drugs market is projected to grow from USD 643 million in 2016 to USD 8.23 billion in 2028, displaying tremendous market demand and business value. It should also be noted that various administration routes including delivery to the lungs,^734^ placenta, targeted nose-to-brain or intranasal,^735^ transdermal diffusion,^736^ and delivery to the eyes^737^ are also being explored. We look forward to more peptide drugs making the journey from laboratory to clinic, clinic to market, and market to patients sooner rather than later, making contributions to human health and well-being.

## Conclusion and perspective

Peptides, with their unique properties, have emerged as a prominent force in innovative drug development. Since the introduction of insulin to the market in 1922, the past two decades have witnessed the approval of nearly a hundred peptide-based drugs for treating conditions such as diabetes, obesity, and cardiovascular diseases. Beyond the well-known insulin and GLP-1 RAs, peptide drugs have also found success in treating rare diseases, exemplified by the approvals of therapies for conditions like acromegaly and hypoparathyroidism. However, most peptide drugs, barring a few exceptions like cyclosporin A, are limited by inherent drawbacks such as in vivo instability and poor bioavailability, confining their administration routes to subcutaneous injection. The recent approvals of oral semaglutide and oral insulin by the Chinese regulatory authority offer renewed hope, underscoring the potential of novel delivery technologies and innovative chemical strategies to bridge the gap between laboratory and bedside.

Transcending their therapeutic applications, peptide-based drugs have also garnered significant attention in drug delivery, vaccine development, and disease diagnostics, with approved diagnostics for conditions like Alzheimer’s disease and certain cancers. Several peptide-based drug conjugates are currently in clinical trials, and various peptide-based vaccines are undergoing Phase III clinical trials, demonstrating their transformative potential. Aided by technologies such as displ ay library screening, efficient and cost-effective synthesis, and the integration of computational biology and artificial intelligence, the discovery of novel peptide drugs, including peptide-drug complexes, new vaccines, and innovative diagnostic reagents, is accelerating. Moreover, a deep understanding of disease pathology and the microenvironment is essential for the rational design of peptide-related drugs, which may enable personalized and precise treatment for individuals.

While challenges undoubtedly lie ahead, we firmly believe that peptides will become a vital branch of innovative pharmaceuticals and biomedical research in the new era. We eagerly anticipate the next generation of peptide drugs and vaccines that will profoundly impact modern medicine, driven by the collective efforts of researchers, clinicians, and industry partners.
